# Multiscale and integrative single-cell Hi-C analysis with Higashi

**DOI:** 10.1038/s41587-021-01034-y

**Published:** 2021-10-11

**Authors:** Ruochi Zhang, Tianming Zhou, Jian Ma

**Affiliations:** Computational Biology Department, School of Computer Science, Carnegie Mellon University, Pittsburgh, PA USA

**Keywords:** Machine learning, Epigenomics

## Abstract

Single-cell Hi-C (scHi-C) can identify cell-to-cell variability of three-dimensional (3D) chromatin organization, but the sparseness of measured interactions poses an analysis challenge. Here we report Higashi, an algorithm based on hypergraph representation learning that can incorporate the latent correlations among single cells to enhance overall imputation of contact maps. Higashi outperforms existing methods for embedding and imputation of scHi-C data and is able to identify multiscale 3D genome features in single cells, such as compartmentalization and TAD-like domain boundaries, allowing refined delineation of their cell-to-cell variability. Moreover, Higashi can incorporate epigenomic signals jointly profiled in the same cell into the hypergraph representation learning framework, as compared to separate analysis of two modalities, leading to improved embeddings for single-nucleus methyl-3C data. In an scHi-C dataset from human prefrontal cortex, Higashi identifies connections between 3D genome features and cell-type-specific gene regulation. Higashi can also potentially be extended to analyze single-cell multiway chromatin interactions and other multimodal single-cell omics data.

## Abstract (web summary)

Single-cell Hi-C analysis with Higashi identifies cell-to-cell variability of 3D genome organization.

## Main

The rapid development of whole-genome mapping methods such as Hi-C^1^ for probing the 3D genome organization inside the nucleus has revealed multiscale higher-order chromatin structures^2^, including A/B compartments^1^, more refined nuclear compartmentalization^3–5^, topologically associating domains (TADs)^6,7^ and chromatin loops^3^. These 3D genome features in different scales are interconnected with vital genome functions, such as gene transcription and DNA replication^8,9^, yet the variation of 3D genome structures and its functional implication in single cells remain mostly unclear^10^. The emerging scHi-C technologies have enabled genomic mapping of 3D chromatin structures in individual cells^11–16^ and, more recently, joint profiling of chromosome conformation with other epigenomic features^17,18^. These exciting scHi-C assays have the potential to comprehensively reveal fundamental genome structure and function connections at single-cell resolution in a wide range of biological contexts.

However, computational methods that can make full use of the sparse scHi-C data to analyze the cell-to-cell variability of 3D genome features are substantially lacking. To account for the sparseness of scHi-C data, methods have been developed for embedding the datasets^19,20^ and the imputation of the contact maps^21^. However, the current state-of-the-art imputation methods based on ‘random walk with restart’, such as scHiCluster^21^, have much room for improvement for a more reliable single-cell 3D genome analysis. Current imputation methods also require storage and calculation on dense matrices with the size of the contact maps in memory, which is impractical when analyzing scHi-C data at relatively high resolutions. It also remains unclear how to reliably compare TAD-like domain boundaries and A/B compartments across single cells to analyze their cell-to-cell variability and functional connections. Therefore, new algorithms are needed to fill these gaps.

Here we report Higashi, a new computational method for multiscale and integrative single-cell Hi-C analysis using hypergraph representation learning. Using the embeddings and the imputed scHi-C contact maps produced by Higashi, we identified cell-to-cell variability of A/B compartment scores and TAD-like domain boundaries that are functionally important. Application to a recent scHi-C dataset of human prefrontal cortex demonstrated the unique ability of Higashi to reveal cell-type-specific 3D genome features in complex tissues. As a new and the most systematic method to date, Higashi enables improved analysis of scHi-C data with the potential to shed new light on the dynamics of 3D genome structures and their functional implications in different biological processes.

## Results

### Overview of Higashi

**Fig. 1:**
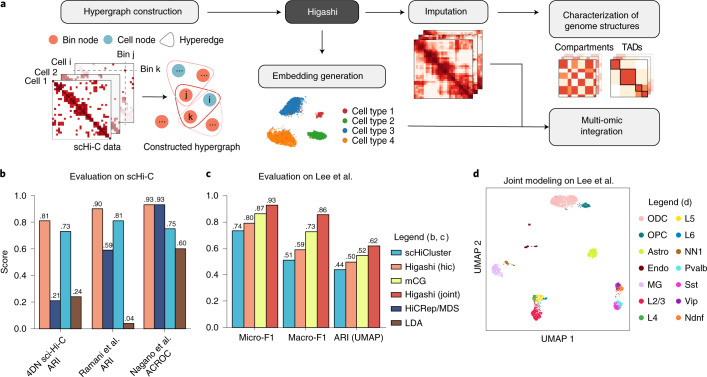
Overview of the Higashi framework for scHi-C analysis. **a**, The input scHi-C dataset is transformed into a hypergraph where each hyperedge connects one cell node and two bin nodes. A hypergraph NN is trained to capture high-order interaction patterns within the constructed hypergraph. The trained NN is able to generate embeddings for scHi-C data and impute the sparse scHi-C contact maps. The imputed contact maps and the embeddings allow detailed characterization of multiscale 3D genome features and also multi-omic integrative analysis. **b**, Quantitative evaluation of Higashi on the three public scHi-C datasets by comparing to HiCRep/MDS^19^, scHiCluster^21^ and LDA^20^. The performances are measured by Adjusted Rand Index (ARI) and also averaged circular ROC (ACROC) scores from the unsupervised cell type identification tasks (see also Supplementary Fig. 3). **c**, Quantitative evaluation of different embeddings of the sn-m3C-seq data^17^ using Micro-F1, Macro-F1 and ARI scores. The embeddings are generated through different embedding methods on scHi-C, the Higashi joint modeling of scHi-C and CG methylation profile (mCG) and the Scanorama^35^ embeddings on mCG. Dimensions of different embedding methods are kept the same for fair comparisons. scHi-C is binned to 1-Mb resolution, whereas mCG is generated at 100-Kb resolution. **d**, UMAP visualization of the Higashi embeddings of the joint modeling of both chromatin conformation and methylation of the sn-m3C-seq data^17^. Cell type abbreviations are in the legend (consistent with ref. ^17^): Astro, astrocyte; Endo, endothelial cell; L2/3, L4, L5 and L6, excitatory neuron subtypes; MG, microglia; Ndnf, Vip, Sst and Pvalb, inhibitory subtypes; NN1, non-neuronal cell; ODC, oligodendrocyte; OPC, oligodendrocyte progenitor cell.

The key algorithmic design of Higashi is to transform the scHi-C data into a hypergraph (Fig. 1a). Such transformation preserves the single-cell resolution and the 3D genome features from the scHi-C contact maps. Specifically, the process of embedding the scHi-C data is now equivalent to learning node embeddings of the hypergraph, and imputing the scHi-C contact maps becomes predicting missing hyperedges within the hypergraph. In Higashi, we use our recently developed Hyper-SAGNN architecture^22^, which is a generic hypergraph representation learning framework, with substantial new development specifically for scHi-C analysis (Methods).

Higashi has five main components. (1) We represent the scHi-C dataset as a hypergraph, where each cell and each genomic bin are represented as cell node and genomic bin node, respectively. Each non-zero entry in the single-cell contact map is modeled as a hyperedge connecting the corresponding cell and the two genomic loci of that particular chromatin interaction (Fig. 1a). This formalism integrates embedding and data imputation for scHi-C. (2) We train a hypergraph neural network (NN) based on the constructed hypergraph (Supplementary Figs. 1 and 2). (3) We extract the embedding vectors of cell nodes from the trained hypergraph NN for downstream analysis. (4) We use the trained hypergraph NN to impute single-cell Hi-C contact maps with the flexibility to incorporate the latent correlations among cells to enhance overall imputation, enabling more detailed and reliable characterization of 3D genome features. (5) With several new computational strategies, we reliably compare A/B compartment scores and TAD-like domain boundaries across individual cells to facilitate the analysis of cell-to-cell variability of these large-scale 3D genome features and its implication in gene transcription. In addition, we developed a visualization tool to allow interactive navigation of the embedding vectors and the imputed contact maps from Higashi to facilitate discovery. The details are described in the Methods.

### Higashi embeddings reflect cell types and cellular states

We sought to demonstrate that Higashi effectively captures the variability of 3D genome structures from the sparse scHi-C data with the embeddings. We first tested our method on three scHi-C datasets with multiple cell types or known cell state information at 1-Mb resolution. These datasets include the 4DN sci-Hi-C dataset^20^, the Ramani et al. dataset^14^ and the Nagano et al. dataset^15^ (see Methods for data processing and Supplementary Tables 1 and 2 for statistics of these datasets). After training, the Higashi embeddings are projected to a two-dimensional space with uniform manifold approximation and projection (UMAP)^23^ for visualization. We found that the Higashi embeddings exhibit clear patterns that correspond to the underlying cell types and cellular states (Supplementary Fig. 3a–c).

We then quantified the effectiveness of the embeddings by various evaluation settings and made direct comparisons to three existing scHi-C embedding methods: HiCRep/MDS^19^, scHiCluster^21^ and LDA^20^ (Supplementary Note A.1). The quantitative results based on unsupervised evaluation suggest that the Higashi embeddings consistently outperform other methods (Fig. 1b). Extensive evaluations under various settings show that the Higashi embeddings can consistently achieve the best performance on scHi-C datasets with either categorical cell types or continuous cell states under various evaluation settings (Supplementary Figs. 3d–f and 4). Although all results in this section are based on the embedding with dimension size 64, our sensitivity analysis on the embedding dimension shows that Higashi is more robust to the choice of dimension size (Supplementary Note A.10 and Supplementary Fig. 5a).

The emerging new technologies that jointly profile chromosome conformation and other epigenomic features have provided unique opportunities to directly analyze 3D genome structures and other modalities at single-cell resolution^17,18^. Higashi has the versatility to incorporate the co-assayed signals into the hypergraph representation learning framework as compared to separate analysis of two modalities, thereby taking full advantage of the co-assayed data (Methods). We applied Higashi to a recently generated co-assayed dataset called single-nucleus methyl-3C sequencing (sn-m3C-seq) that jointly profiles Hi-C and DNA methylation in individual human prefrontal cortex cells^17^. We found that the Higashi embeddings trained only on scHi-C (referred to as ‘Higashi (hic)’) can already resolve complex cell types in this dataset (Figs. 1c and 4a,b; detailed results will be discussed in a later section). When using Higashi to jointly model both signals (the embeddings referred to as ‘Higashi (joint)’), it reaches the overall best performance as compared to the embeddings based on only one modality (Fig. 1c and Supplementary Fig. 6; see Supplementary Note A.1 for details on embedding generation). Higashi (joint) shows clearer patterns in the UMAP with cells being aggregated according to their cell types (Fig. 1d). Note that, here, the co-assayed methylation profiles are not part of the input to the NN but serve as the targets to approximate (Methods).

Taken together, these results demonstrate that the Higashi embeddings effectively capture the cell-to-cell variability of 3D genome structures based on scHi-C data to reflect the underlying cellular states. In addition, the unique capability of Higashi for the joint modeling of both scHi-C and methylation profiles further enhances the scHi-C embeddings.

### Higashi robustly imputes scHi-C contact maps

In addition to dimension reduction of scHi-C data for cell type identification, Higashi can also impute sparse scHi-C contact maps. Here, we sought to demonstrate the imputation accuracy with several evaluations. For comparisons, we included the imputed results from scHiCluster. Note that scHiCluster represents each scHi-C contact map as an individual graph, whereas Higashi represents the whole scHi-C dataset as a hypergraph, allowing imputation to be potentially coordinated across different cells. Specifically, in Higashi, when imputing the contact map of cell *i*, its *k*-nearest neighbors in the embedding space would contribute to the imputation by taking advantage of their latent correlations (Methods). To demonstrate the advantages of this design employed in Higashi, we included the imputed results from Higashi with *k* as 0 and 4 (referred to as ‘Higashi(0)’ and ‘Higashi(4)’, respectively). We performed sensitivity analysis on the hyperparameter *k* and showed that Higashi is highly robust to the choice of *k* (Supplementary Note A.10 and Supplementary Fig. 5b).

**Fig. 2:**
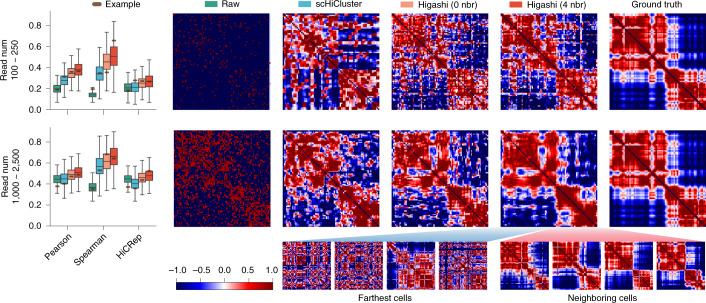
Evaluation and visualization of different imputation methods on scHi-C data simulated from multiplexed STORM 3D genome imaging data^24^. For Higashi, results by using information from four neighboring cells (4 nbr) or without using neighboring cell information (0 nbr) in the embedding space are both included. Each row corresponds to one set of simulation data with a chosen range of read numbers. The box plots illustrate the quantitative evaluation of the similarities by comparing the raw (input), the scHiCluster enhanced and the Higashi enhanced contact maps against the ground truth (inverse distance map). In the box plots, the middle line is the median; the lower and upper lines correspond to the first and third quartiles; and the upper and lower whiskers extend to values no farther than 1.5× IQR. The heat maps visualize the contact map before and after imputation as well as the ground truth. The contact maps of both the neighboring cells (in the embedding space) that contribute to the imputation and the cells that are farthest (in the embedding space) are shown. See also Supplementary Fig. 8. IQR, interquartile range.

We developed a simulation evaluation method to make use of the multiplexed 3D genome imaging data, which provides high-resolution physical views of 3D organization of genomic loci in individual cells^24^. Specifically, we turned the imaging data of a 2.5-Mb region on chr21 from 11,631 cells at 30-Kb resolution into scHi-C contact maps with various simulation coverage (Supplementary Note A.4 and Supplementary Fig. 7). We found that Higashi(0)—that is, no information sharing among different cells—can already consistently outperform scHiCluster. In addition, we found that Higashi(4) improves the imputation most significantly (30–43% improvement on the median similarities across multiple metrics on the dataset with the lowest coverage). To illustrate why using neighboring cells in the embedding space improves imputation, we show a typical example from the simulated data with contact maps before and after imputation (Fig. 2 and Supplementary Fig. 8). Consistent with the quantitative evaluation, Higashi(4) shows the clearest patterns and identifies domain boundaries across all coverage (Fig. 2 and Supplementary Fig. 8). The neighboring cells in the embedding space that contribute to the imputation indeed have similar 3D chromatin interactions compared to the selected cell, whereas the farthest cells do not. We carried out a similar set of evaluation using the more recent multiplexed imaging data of 3D genome structure^25^ (3,029 simulated contact maps of chr2 at 1-Mb resolution; see the statistics of scHi-C datasets that we used as reference for the simulation coverage in Supplementary Table 3) and reached the same conclusion of Higashi’s clear advantage (22–50% improvement on the median similarities across multiple metrics on the dataset with the lowest coverage; Supplementary Figs. 5c and 9).

We performed additional evaluation via downsampling the existing scHi-C datasets with relatively higher coverage (Supplementary Note A.4). We used the WTC-11 scHi-C dataset (personal communication with Bing Ren) of chr1 at 1-Mb resolution and downsampled the sequencing reads of each cell at different rates (Supplementary Note A.4 and Supplementary Tables 1 and 4). We again observed clear advantages of Higashi for imputation, with the strongest performance achieved by Higashi(4) (consistent advantage with up to 89% improvement on the distance stratified Spearman correlation; Supplementary Fig. 10).

We further evaluated Higashi by (1) comparing the Higashi imputations to the imputation results of 3D structure modeling under different coverage and (2) comparing the pooled single-cell contact maps imputed by Higashi to the true bulk Hi-C data (Supplementary Notes A.11 and A.12 and Supplementary Figs. 11 and 12). These results again confirmed the robustness and advantages of the Higashi imputation.

Together, these evaluations demonstrate that Higashi achieves much improved imputation of scHi-C contact maps robustly. The performance is further enhanced by the unique mechanism of sharing information among neighboring cells in the embedding space. The improved imputation enables more reliable analysis of 3D genome structural features of each individual cell with higher accuracy.

### Higashi identifies compartmentalization variability

Next, we explored how the enhanced contact maps produced by Higashi facilitate multiscale 3D genome analysis at single-cell resolution. A/B compartments reflect large-scale chromosome spatial segregation with distinct connections to genome function^1^. To date, little progress has been made for systematic A/B compartment annotation using scHi-C data, primarily because of the data sparseness. Here, we applied Higashi to impute the WTC-11 scHi-C data at 50-Kb resolution (see examples of the imputation results in Supplementary Fig. 13). We designed a method to calculate continuous compartment scores such that the scores are directly comparable across the cell population and reflect detailed cell-to-cell variation (Supplementary Note A.5).

**Fig. 3:**
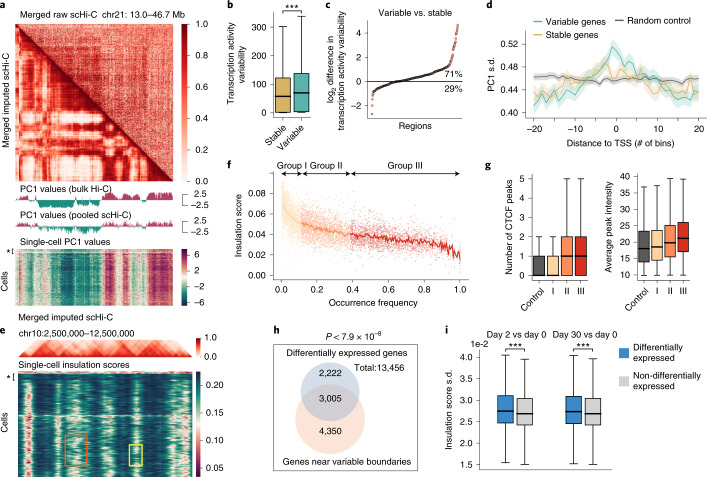
Higashi enables detailed characterization of 3D genome features and their connections to gene transcription at single-cell resolution. **a**, Compartment score annotations for WTC-11 scHi-C data at 50-Kb resolution. The merged scHi-C correlation matrix of chr21 (before and after imputation), as well as the compartment scores called from the bulk Hi-C contact map, the pooled scHi-C contact map and each single-cell contact map, are shown. The cells that are likely near the mitosis stage are marked with ‘*’ in the single-cell PC1 heat map. **b**, Global comparisons of transcriptional variability on regions with variable and stable compartment annotations (*** indicates *P* < 1 × 10^−3^). *n* = 10,146 genes used for the comparison. There are 5,071 genes that have stable single-cell compartment scores, with average transcription activity variability equal to 77.4. There are 5,075 genes that have dynamic single-cell compartment scores, with average transcription activity variability equal to 86.0. The middle line is the median; the lower and upper lines correspond to the first and third quartiles; and the upper and lower whiskers extend to values no farther than 1.5× IQR. One-sided *t*-test, *P* = 1.34 × 10^−7^. **c**, log_2_ difference of transcriptional variability of genes with variable versus stable compartment annotations within an Mb-scale window. **d**, Visualization of standard deviation of compartment scores around genes with variable or stable transcriptional level. The data are presented as mean values ± 1.96 s.e.m. (95% confidence interval). In **b**–**d**, the transcriptional variability is quantified as the CV of the imputed scRNA-seq data. **e**, TAD-like domain boundary calling for WTC-11 scHi-C at 50-Kb resolution. The merged scHi-C contact maps at chr10:2,500,000–12,500,000 and the calculated insulation scores are shown. The cells that are likely near the mitosis stage are marked with ‘*’ in the single-cell insulation score heat map. Regions that represent the present/absent dynamics of single-cell domain boundaries are marked with a yellow box. Regions that represent the sliding dynamics of single-cell domain boundaries are marked with an orange box. **f**, Scatter plot of the single-cell insulation scores versus the occurrence frequency in the cell population of shared domain boundaries. For each cell, only the insulation scores of presented shared boundaries are visualized—that is, each dot corresponds to a single-cell domain boundary. **g**, CTCF binding at domain boundaries from different occurrence frequency groups. For the left panel: *n* = 8,004 boundaries in total, including 1,577 in the control group, 2,137 in group I, 2,127 in group II and 2,163 in group III. For the right panel: *n* = 4,434 boundaries with at least one CTCF binding, including 639 in the control group, 895 in group I, 1,408 in group II and 1,592 in group III. In the box plot, the middle line is the median; the lower and upper lines correspond to the first and third quartiles; and the upper and lower whiskers extend to values no farther than 1.5× IQR. **h**,Venn diagram of the overlap between genes near the variable domain boundary in WTC-11 (light red) and DEGs during cell differentiation (light blue). Hypergeometric test (*P* ≤ 7.9 × 10^−8^). **i**, Comparison of cell-to-cell variability of insulation scores between DEGs and non-DEGs. The high variance of insulation scores of DEGs indicates that the DEGs are enriched near domain boundaries with higher variability (*** indicates *P* < 1 × 10^−3^). Day 2 versus day 0: *n* = 13,467 genes in total, including 3,205 DEGs and 10,262 non-DEGs, with mean insulation score standard deviation equal to 2.83 × 10^−2^ and 2.74 × 10^−2^, respectively. One-sided *t*-test, *P* = 2.23 × 10^−9^. Day 30 versus day 0: *n* = 13,467 genes in total, including 4,308 DEGs and 9,159 non-DEGs, with mean insulation score standard deviation equal to 2.80 × 10^−2^ and 2.74 × 10^−2^, respectively. In the box plot, the middle line is the median; the lower and upper lines correspond to the first and third quartiles; and the upper and lower whiskers extend to values no farther than 1.5× IQR. One-sided *t*-test, *P* = 4.16 × 10^−6^. IQR, interquartile range; TSS, transcription start site.

Figure 3a shows the merged correlation matrices (Pearson correlation of the merged contact maps) before and after Higashi imputation, as well as the compartment scores from the bulk Hi-C, the compartment scores from the pooled scHi-C and the single-cell compartment scores of chr21. After imputation, the merged scHi-C correlation matrix has much clearer checkerboard patterns that correspond to A/B compartments. The calculated single-cell compartment scores are overall consistent with the bulk compartment scores (Supplementary Fig. 14) while showing cell-to-cell variability. Note that we identified one cluster of cells in the heat map that has distinct patterns and is likely near the mitosis stage (marked with ‘*’ in the bottom panel of Fig. 3a).

We explored the connection between the variability of compartment scores across the cell population and the transcriptional activity in different cells. We compared the compartment scores with the single-cell RNA sequencing (scRNA-seq) from WTC-11 (ref. ^26^). For this analysis, the cells that are likely near the mitosis stage were removed. For each gene, the transcriptional variability was calculated using the coefficient of variation (CV) (Supplementary Note A.6). We quantified the compartment variability as the standard deviation of the single-cell compartment scores and further classified the expressed genes as compartment variable or stable with a cutoff of 50% based on the quantile. Compared with the transcriptional variability within these two groups (Fig. 3b), we observed that the genes in more variable compartments have higher transcriptional variability (*P* < 0.001). We then used the 50-Mb window resolution to assess if such structure–function variability correlation can also be observed at a finer scale. We used a 50-Mb sliding window with a 1-Mb step size on each chromosome and calculated the log difference of the median transcriptional variability between the variable and stable compartment regions within this window. As shown in Fig. 3c, among all windows, 71% of them follow the trend that genes in compartment variable regions have higher transcriptional variability. As a comparison, ~76% of the genomic windows exhibit that the bulk compartment A correlates with higher expression levels^1^ (Supplementary Fig. 15d). In addition, we made a step further to increase the resolution to individual genes. We classified genes as locally variable or stable by identifying the local minima/maxima of the transcriptional variability. We found that, for the genes that are locally variable in terms of transcription, their compartment variability scores also tend to be the local maximum (Fig. 3d).

To confirm the robustness of these observations, in addition to using CV to measure transcriptional variability, we used another metric based on a variance stabilizing algorithm (Supplementary Note A.6) and reached similar conclusions (Supplementary Fig. 15a–c). These results further demonstrate the reliability of Higashi imputations, identifying cell-to-cell variability of compartment scores that are also functionally correlated.

### Higashi unveils single-cell TAD-like domain boundaries

Recent work based on multiplexed STORM imaging of chromatin conformation demonstrated the existence and cell-to-cell variability of TAD-like structures in single cells^24^. However, the identification of TAD-like domains remains extremely challenging for sparse scHi-C data. We developed an approach to identify TAD-like domain boundary variability from single cells based on the Higashi imputations (Supplementary Notes A.7 and A.8 and Supplementary Fig. 16). The analysis was conducted on the WTC-11 scHi-C dataset at 50-Kb resolution.

We calculated single-cell insulation scores in which the local minima correspond to TAD-like domain boundaries^27^ (Fig. 3e). As compared to the single-cell insulation scores calculated from the raw scHi-C, the single-cell insulation scores based on the imputed contact maps show more consistent patterns with the TAD boundaries identified at the population level and allow more reliable TAD-like domain boundary calling at single-cell resolution (Supplementary Fig. 17). We again observed a cluster of cells likely near the mitosis stage showing unidentifiable domain boundaries (marked with ‘*’ in the bottom panel of Fig. 3e). We also observed that the local minima of the single-cell insulation scores often center around the domain boundaries observed in the merged imputed scHi-C, whereas the exact locations of the single-cell boundaries vary across the cell population (Fig. 3e). The dynamics of the single-cell domain boundaries have two main patterns: (1) present/absent across the population (marked with a yellow box in Fig. 3e) and (2) sliding along the genome (marked with an orange box in Fig. 3e). The first pattern reflects that a domain boundary does not occur in all cells. The second pattern manifests the shift of domain boundary along the genome, suggesting more gradual cell-to-cell variability. Comparison with scRNA-seq following the same approach used for single-cell compartment scores reached similar conclusions, that domain boundary variability is strongly correlated with transcriptional variability at different scales (Supplementary Fig. 15e–j).

Next, we made direct comparisons of TAD-like domain boundaries (Supplementary Note A.8). As shown in Fig. 3f, where each dot corresponds to a single-cell domain boundary, we observed a negative correlation between the occurrence frequency of a domain boundary with its median single-cell insulation scores. This suggests that the more stable domain boundaries (that is, higher occurrence frequency) from the cell population tend to be ‘stronger’ boundaries in single cells associated with lower insulation scores. We also found positive correlation between the occurrence frequencies of domain boundaries and the number of CTCF binding peaks as well as the average CTCF peak intensity in the boundaries (Fig. 3g, Supplementary Fig. 18 and Supplementary Note A.13). This result is consistent with the observation based on multiplexed STORM imaging^24^.

As an induced pluripotent stem cell (iPSC) type, WTC-11 can undergo cell differentiation. We identified differentially expressed genes (DEGs) from an scRNA-seq dataset of WTC-11 cells at five differentiation stages^26^ (Supplementary Note A.9). Using hypergeometric test, we found that DEGs are over-represented in genes located near more variable domain boundaries in WTC-11 (top 50% of the insulation score standard deviation, *P* ≤ 7.9 × 10^−8^) (Fig. 3h). In addition, we compared the variability of insulation scores between DEGs and non-DEGs and found that DEGs have markedly higher standard deviation (one-sided *t*-test, *P* < 0.001) (Fig. 3i). This suggests that the cell-to-cell variability of domain boundaries in WTC-11 might indicate functional implications in cell differentiation.

Taken together, by analyzing the TAD-like domain boundaries across single cells enabled by Higashi, we identified a correlation between domain boundary variability and gene regulation at single-cell resolution.

### Single-cell 3D genome features in human prefrontal cortex

To demonstrate Higashi’s ability to analyze single-cell 3D genome structures for complex tissues, we applied it to the aforementioned sn-m3C-seq data from human prefrontal cortex^17^. In this section, we present results from the Higashi framework trained only by the chromatin conformation information in sn-m3C-seq at 100-kb resolution to evaluate its unique strength in analyzing scHi-C data.

**Fig. 4:**
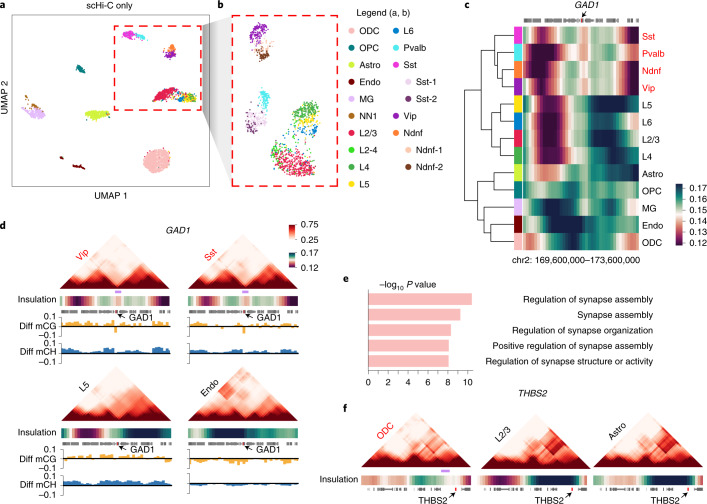
Higashi identifies complex cell types and cell-type-specific TAD-like domain boundaries using scHi-C data from human prefrontal cortex. **a**, UMAP visualization of the Higashi embeddings using scHi-C only. **b**, UMAP visualization of the Higashi embeddings of the neuron subtypes in **a**. Cell type information is from ref. ^28^. Subtypes L2–4, Sst1/2 and Ndnf1/2 are only used in this subfigure. **c**, Hierarchical clustering based on the average single-cell insulation scores of the flanking regions (± 2 Mbp) of the marker gene GAD1 for inhibitory neuron subtypes Sst, Pvalb, Ndnf and Vip. Note that the single-cell insulation scores are calculated based on the Higashi imputed contact maps trained using only scHi-C data. **d**, Pooled imputed contact maps, average single-cell insulation scores and methylation profiles of the same region in **c** for selected cell types. The methylation profile is calculated as the average CG/non-CG methylation percentage of a specific cell type minus the average CG/non-CG methylation percentage of the whole population. The light purple bar shows a TAD-like domain boundary specific to inhibitory neuron subtypes. **e**, Top five enriched GO terms near ODC-specific TAD-like domain boundaries. The enrichment analysis and the corresponding *P* values are from GREAT, which uses bionomial tests. **f**﻿, Pooled imputed contact maps, insulation scores and methylation profiles near the gene THBS2, which is in four of the top five most enriched GO terms with ODC-specific high expression. The light purple bar shows an ODC-specific TAD-like domain boundary. Cell type abbreviations are in the legend (consistent with ref. ^17^): Astro, astrocyte; Endo, endothelial cell; L2/3, L4, L5 and L6, excitatory neuron subtypes; MG, microglia; Ndnf, Vip, Sst and Pvalb, inhibitory subtypes; NN1, non-neuronal cell type 1; ODC, oligodendrocyte; OPC, oligodendrocyte progenitor cell.

We found that the Higashi embeddings (with scHi-C only) are able to resolve the differences among the neuron subtypes (separating Pvalb, Sst, Vip, Ndnf, L2/3 and L4–6) while maintaining clear separation with non-neuron cell types (Fig. 4a; embedding dimension = 128). This suggests that, analyzed with Higashi, scHi-C alone has sufficient information to distinguish complex neuron subtypes. In contrast, scHiCluster cannot clearly distinguish these neuron subtypes using scHi-C (Fig. 5c in ref. ^17^). We further obtained refined cell subtype information from ref. ^28^, where the methylation profiles of the sn-m3c-seq dataset are jointly embedded with single-cell methylation profiles from snmC-seq, snmCT-seq and snmC2T-seq on human prefrontal cortex to annotate cell types, resulting in more detailed cell type labels on the sn-m3c-seq dataset. When visualizing only the neuron cells with UMAP and the refined cell type labels based on ref. ^28^ (Fig. 4b), we observed clearer separation among neuron subtypes, especially for L2/3, L4, L5 and L6. We also observed smaller clusters of Sst and Ndnf subtypes (denoted as Sst-1/2 and Ndnf-1/2 in Fig. 4b). In addition, a recent approach has been proposed to separate neuron subtypes on a dataset based on Dip-C with much higher coverage per cell^29^. However, we found that, for the sn-m3c-seq dataset, the method developed in ref. ^29^ cannot distinguish neuron subtypes (Supplementary Fig. 19 and Supplementary Note A.14), further confirming the advantages of Higashi.

Next, we sought to identify cell-type-specific 3D genome structures with the Higashi imputed contact maps. Here, the Higashi model was trained with the hyperparameter *k* = 4. During imputation, we also used the batch effects removal mechanism in Higashi because one of the three batches in the sn-m3c-seq dataset has smaller sequencing depths that could cause potential bias for the downstream analysis (Methods). When analyzing cell-type-specific 3D genome features, we used the original cell type labels from ref. ^17^ to make sure that each cluster has enough cells to reveal consistent 3D genome patterns. Our analysis identifies global connections among multiscale cell-type-specific genome structures (that is, single-cell A/B compartments and single-cell TAD-like domain boundaries) with the transcriptional activity of marker genes (Supplementary Note A.15 and Supplementary Figs. 20 and 21), further suggesting Higashi’s potential for annotating cell types from complex tissues based on scHi-C. We then specifically investigated the connection between TAD-like domain boundaries and individual marker genes. For instance, the single-cell insulation scores of the region surrounding the transcription start site of the marker gene GAD1 in inhibitory neuron subtypes reflect much stronger TAD-like domain boundaries (Fig. 4c). Note that such cell-type-specific patterns are obscured in the pooled population contact maps (Supplementary Fig. 22a, top). Although aggregating raw single-cell contact maps and the corresponding insulation scores by cell types can reveal similar patterns at the population level (Supplementary Fig. 23), our analysis shows that the single-cell insulation scores calculated based on Higashi imputed contact maps (with *k* = 0 or 4) have the power to separate complex cell types, whereas the single-cell insulation scores based on raw contact maps cannot distinguish cell types robustly (Supplementary Fig. 24). The cell-type-specific domain boundary pattern is further manifested by comparison to the contact maps and methylation profiles (Fig. 4d and Supplementary Fig. 25; light purple bars indicate cell-type-specific domain boundaries). In addition, we found that SULF1, which is a marker gene to distinguish subtypes L6 from the rest excitatory neuron subtypes (L2/3, L4 and L5), has a strong correlation with the surrounding cell-type-specific TAD-like domain boundaries and methylation profiles (Supplementary Figs. 22b and 26). Specifically, the TAD-like domain boundary is present in 93.2% of L6 cells but in only 65.3% of the rest of excitatory neuron subtypes. These results provide new insights into the marker gene regulation of human prefrontal cortex cell types and the connection between 3D genome structure and function.

We next asked whether the genes near cell-type-specific TAD-like domain boundaries identified by Higashi have distinct functional roles. We found that genes close to the oligodendrocyte (ODC)-specific domain boundaries (784 in total) are strongly enriched with synapse-related Gene Ontology (GO) terms as top hits (Fig. 4e; using the Genomic Regions Enrichment of Annotations Tool (GREAT)^30^), suggesting the functional role of ODC-specific domain boundaries in regulating synaptic functions^31^. To further analyze the connection between the ODC-specific domain boundaries and the regulation of the nearby genes, we investigated the gene THBS2, which appears in four of the top five GO term categories that we identified. THBS2 is known to be expressed in glial cells and is key to the regulation of synaptic functions^32^. The visualization of the pooled contact maps of the 4-Mb region surrounding THBS2 shows that ODCs have a TAD-like domain boundary upstream of the transcription start site of THBS2 (Fig. 4f and Supplementary Fig. 27), which can be elucidated by single-cell insulation scores of this region (Supplementary Fig. 22c, top). Notably, the TAD-like domain boundary near THBS2 is obscured in the insulation score calculated from the population contact map (Supplementary Fig. 22c). Note that THBS2 has cell-type-specific high expression in ODC (fold change of 8.6 compared to the population average)^33^. Therefore, the ODC-specific TAD-like domain boundaries might offer new perspectives for understanding the cell-type-specific gene regulation of THBS2.

Taken together, these results demonstrate the distinct ability and advantages of Higashi to effectively identify cell types and cell-type-specific 3D genome features in complex tissues using scHi-C data. This analysis shows the strong potential of Higashi in revealing cell-type-specific TAD-like domain boundaries, greatly facilitating the analysis of the roles of 3D genome structure in regulating cell-type-specific gene function.

## Discussion

In this work, we developed Higashi for multiscale and integrative scHi-C analysis. Our extensive evaluation demonstrated the advantages of Higashi over existing methods for both embedding and imputation. Additionally, enabled by the improved data enhancement of scHi-C contact maps, we developed methods in Higashi to systematically analyze variable multiscale 3D genome features (A/B compartment scores and TAD-like domain boundaries), revealing their implications in gene transcription. By applying to an scHi-C dataset from human prefrontal cortex, Higashi is able to identify complex cell types and reveal cell-type-specific TAD-like domain boundaries that have strong connections to cell-type-specific gene regulation.

The key algorithmic innovation of Higashi is the transformation of scHi-C data into a hypergraph, which has unique advantages compared to existing methods. First, this transformation preserves the single-cell precision and 3D genome features from scHi-C. Second, modeling the whole scHi-C datasets as a hypergraph instead of modeling each contact map as individual graphs allows information to be coordinated across cells to improve both embedding and imputation by taking advantage of the latent correlations among cells. Third, although we mainly focused on scHi-C data, the hypergraph representation in Higashi is highly generalizable to other single-cell data types. As a proof of principle, we showed that Higashi can be extended to analyze co-assayed scHi-C data with methylation in an integrated manner, showing markedly improved performance compared to separate analysis of the two modalities.

There are several directions that Higashi can be further enhanced. As a data-driven method, despite the unique ability of using information from neighboring cells in the embedding space, Higashi requires at least a moderate-size scHi-C dataset to achieve high performance. Moreover, even though Higashi has clear advantages in imputing the scHi-C contact maps using hypergraph representation learning compared to existing methods, there is still much room for improvement regarding the imputation of long-range interactions (≥10 Mb) due to their highly diverse nature in single-cell 3D genome structures. Methods that can robustly impute these long-range interactions or even inter-chromosomal interactions are expected to further advance the understanding of single-cell 3D genome organization and its functional implication. In addition, to achieve more comprehensive delineation of 3D genome organization at single-cell resolution, Higashi can be potentially extended to analyze single-cell assays of higher-order chromatin structures—for example, the recently developed scSPRITE^34^ that probes multiway chromatin interactions.

## Methods

### scHi-C data and other genomic data processing

In this work, we used several publicly available single-cell Hi-C datasets. We refer to them as Ramani et al.^14^ (Gene Expression Omnibus (GEO): GSE84920), Nagano et al.^15^ (GEO: GSE94489) and 4DN sci-Hi-C^20^ (4DN Data Portal: 4DNES4D5MWEZ, 4DNESUE2NSGS, 4DNESIKGI39T, 4DNES1BK1RMQ and 4DNESTVIP977). We also used a new scHi-C dataset generated from the WTC-11 iPSC line (4DN Data Portal: 4DNESF829JOW and 4DNESJQ4RXY5).

For all scHi-C datasets, we kept only the cells with more than 2,000 read pairs that have genomic span greater than 500 Kb. At a given resolution, we define the number of contacts per cell as the number of interaction pairs (read count) assigned to the non-diagonal entries of the intra-chromosomal contact maps. The Ramani et al. dataset and the 4DN sci-Hi-C dataset used single-cell combinatorial indexed Hi-C (sci-Hi-C).

After filtering, the Ramani et al. dataset contains 620 cells of four human cell types (GM12878, HAP1, HeLa and K562) with 7,800 median contacts per cell, whereas the 4DN sci-Hi-C dataset contains 6,388 cells of five human cell types (GM12878, H1ESC, HAP1, HFFc6 and IMR90) with 3,800 median contacts per cell. The Nagano et al. dataset used a different protocol with 1,171 cells and 56,800 median contacts per cell. The WTC-11 scHi-C dataset (188 cells in total) was generated using single-nucleus Hi-C with 144,800 median contacts per cell. The interaction pairs from the Nagano et al. and Ramani et al. datasets were downloaded from the corresponding GEO repository. The interaction pairs for WTC-11 were obtained through personal communication with Bing Ren. For 4DN sci-Hi-C, we downloaded the FASTQ files and processed them with the recommended processing pipeline (https://github.com/VRam142/combinatorialHiC). The interaction pairs can be directly used as input for Higashi.

The co-assayed single-cell methylation and Hi-C dataset (sn-m3C-seq) was from ref. ^17^. We followed the same processing pipeline as sn-m3C-seq for processing the methylation signals. We obtained the 10-kb processed contact maps from ref. ^17^ and used them as input for Higashi. The corresponding cell type information was obtained from ref. ^17^ as well. The refined cell type information for the sn-m3c-seq dataset was from ref. ^28^, where the methylation profiles of the sn-m3c-seq dataset are jointly embedded with single-cell methylation profiles from snmC-seq, snmCT-seq and snmC2T-seq on human prefrontal cortex to annotate cell types. We then merged the small clusters with fewer than 30 cells in the sn-m3c-seq dataset for better visualization and more robust analysis. For all datasets, only intra-chromosomal contacts were used to make fair comparisons with other methods. In principle, Higashi can include inter-chromosomal interactions as well by adding the corresponding hyperedges to the model. However, the amount of inter-chromosomal contacts in scHi-C data is generally not sufficient for reliable imputation and analysis.

We also used other publicly accessible genomic datasets in this work. The bulk Hi-C of WTC-11 was obtained from the 4DN Data Portal (4DNESPDEZNWX and 4DNESJ7S5NDJ; two clones were merged before calculating bulk compartment scores). The scRNA-seq of WTC-11 was from ref. ^26^. The details on calculating transcriptional variability based on scRNA-seq can be found in Supplementary Note A.6. We also analyzed the CTCF binding near the identified single-cell TAD-like domain boundaries in WTC-11 cells. We used the WTC-11 CTCF ChIA-PET data (4DN Data Portal: 4DNES8MZ76GP) and called peaks based on the singleton reads from the dataset following the ENCODE ChIP-seq peak calling pipeline^36^. Specifically, peaks were generated for individual replicates and merged by keeping only the reproducible peaks. The scRNA-seq of multiple cortical areas of the human brain was obtained from the Allen Brain map^33,37^.

### Hypergraph NN architecture in Higashi

A *hypergraph* *G* is a generalization of a graph and can be formally defined as a pair of sets *G* = (*V*, *E*), where *V* = {*v*_*i*_} represents the set of nodes in the graph, and $E=\{{e}_{i}=({v}_{1}^{(i)},...,{v}_{k}^{(i)})\}$ represents the set of hyperedges. For any hyperedge *e* ∈ *E*, it connects two or more nodes (∣*e*∣≥2). Both nodes or hyperedges can have attributes reflecting the associated properties, such as node type or the strength of a hyperedge. The *hyperedge prediction problem* aims to learn a function *f* that can predict the probability of a group of nodes (*v*_1_, *v*_2_, . . . , *v*_*k*_) forming a hyperedge or the attributes associated with the hyperedge. For simplicity, we refer to both cases as predicting the probabilities of forming a hyperedge.

The core part of Higashi is a hypergraph representation learning framework, extending our recently developed Hyper-SAGNN^22^ that models higher-order interaction patterns from the hypergraph constructed from the scHi-C data. The model aims to predict the value of an entry (that is, contact frequency) in an scHi-C contact map using the rest of the contact map as input. The model also has the option to use the contact maps from cells that share similar 3D genome structures (that is, close to each other in the embedding space) as auxiliary information for the prediction as well. This setting shares similarity with the self-supervised learning on graphs^38^ where a proportion of the graph is masked randomly, and the NN is trained to recover the masked part with the rest of the graphs. The overall structure of the hypergraph NN is illustrated in Supplementary Fig. 1. We use *x*_*i*_ to represent the attributes of node *v*_*i*_. The input to the model is a triplet—that is, (*x*_1_, *x*_2_, *x*_3_)—consisting of attributes of one cell node and two genomic bin nodes. For simplicity, we do not differentiate between these two types of nodes in this section. Each node within a triplet passes through an NN, respectively, to produce (*s*_1_, *s*_2_, *s*_3_), where *s*_*i*_ = NN_1_(*x*_*i*_). The structure of NN_1_ used in this work is a position-wise feed-forward NN with one fully connected layer. By definition, each *s*_*i*_ remains the same for node *v*_*i*_ independent to the given triplet and is, thus, called the ‘static embedding’, reflecting the general topological properties of a node in the given hypergraph. In addition, the triplet as a whole also passes through another transformation, leading to a new set of vectors (*d*_1_, *d*_2_, *d*_3_), where *d*_*i*_ = NN_2_(*x*_*i*_∣(*x*_1_, *x*_2_, *x*_3_)). The structure of NN_2_ will be discussed later. The definition of *d*_*i*_ depends on all the node features within this triplet that reflect the specific properties of a node *v*_*i*_ in a particular hyperedge and is, thus, called the ‘dynamic embedding’.

Next, the model uses the difference between the static and dynamic embeddings to produce ${\hat{y}}_{i}$ by passing the Hadamard power of *d*_*i*_ − *s*_*i*_ to a fully connected layer. Additional features, including the genomic distance between the bin pair, one hot encoded chromosome ID, batch ID when applicable and also the total read number per cell, are concatenated and sent to a multi-layer perceptron with output ${\hat{y}}_{{{\mathrm{ext}}}}$. All the output ${\hat{y}}_{i}$ and ${\hat{y}}_{{{\mathrm{ext}}}}$ are further aggregated to produce the final result $\hat{y}$—that is, the predicted probability for this triplet to be a hyperedge:

1 $$\hat{y}={\hat{y}}_{{{\mathrm{ext}}}}+\mathop{\sum }\limits_{i=1}^{3}{\hat{y}}_{i}={\hat{y}}_{{{\mathrm{ext}}}}+\mathop{\sum }\limits_{i=1}^{3}{{\mbox{FC}}}\,\left[{({d}_{i}-{s}_{i})}^{\circ 2}\right]$$

where FC is the fully connected layer.

In the following sections, we describe how the node attributes are generated, the structure of NN_2_, the model training and how we incorporate co-assayed signals into Higashi.

### Node attribute generation in Higashi

As mentioned, the input to the hypergraph NN model is a triplet consisting of attributes of one cell node and two genomic bin nodes. For the bin nodes, we use the corresponding rows of the merged scHi-C contact maps as the attributes. For the cell nodes, we calculate a feature vector based on its scHi-C contact maps as its attributes. This process is as follows:

1. Each contact map is normalized based on the total read count.
2. Contact maps are flattened into one-dimensional vectors and concatenated across the cell population.
3. (optional) Singular value decomposition is used to reduce dimensions for computational efficiency.
4. The corresponding row in the feature matrix is used as the attributes for the corresponding cell.

For computational efficiency, we calculate the feature vectors for cell nodes in low-resolution scHi-C contact maps (such as 1 Mb or 500 Kb) when training Higashi for high-resolution imputation.

### Cell-dependent graph NN for dynamic embeddings

Here, we introduce NN_2_ (mentioned above) that transforms the attributes of a node given a node triplet to the corresponding dynamic embeddings. In the original Hyper-SAGNN, this was accomplished by a modified multi-head self-attention layer^39^. This self-attention layer functions as follows. Given a group of nodes (*x*_1_, *x*_2_, *x*_3_) and weight matrices *W*_*Q*_, *W*_*K*_, *W*_*V*_ to be trained, the model first computes the attention coefficients that reflect the pairwise importance of nodes:

2 $${e}_{ij}={\left({W}_{Q}^{T}{x}_{i}\right)}^{T}\left({W}_{K}^{T}{x}_{j}\right),\forall 1\le i,j\le 3,i\ne j$$

These coefficients then normalize *e*_*ij*_ by all possible *j* within the tuple through the softmax function. Finally, a weighted sum of the transformed features with an activation function is calculated:

3 $${\alpha }_{ij}=\frac{\exp ({e}_{ij})}{{\sum }_{1\le l\le k,l\ne i}\exp ({e}_{il})}$$

4 $${d}_{i}=\tanh \left(\mathop{\sum}\limits_{1\le j\le k,j\ne i}{\alpha }_{ij}{W}_{V}^{T}{x}_{j}\right)$$

However, the representation capacity of using self-attention layers to calculate dynamic embeddings is constrained by the embedding dimensions and the depth of self-attention layers, which would lead to high computational cost and increased training difficulty.

To increase the expressiveness of this NN for generating dynamic embeddings while maintaining small embedding dimensions and fewer layers, we developed a cell-dependent graph neural network (GNN)^40^ that transforms the attributes of bin nodes before passing to the self-attention layer. For a node triplet (*c*_*i*_, *b*_*j*_, *b*_*k*_), where *c*_*i*_ corresponds to a cell node and *b*_*j*_, *b*_*k*_ are bin nodes, a graph *G*(*c*_*i*_) (where both *b*_*j*_, *b*_*k*_ are nodes in it) is constructed by taking *c*_*i*_ as input. Details on the construction of *G*(*c*_*i*_), which is shared for all triplets that contain the cell node *c*_*i*_, is discussed in the next section. For each layer in the GNN, to generate the output vector for bin node *b*_*j*_, the information of its neighbors in the graph ${{{{\mathcal{N}}}}}_{G({c}_{i})}({b}_{j})$ is aggregated:

5 $${H}_{{{{{\mathcal{N}}}}}_{G({c}_{i})}({b}_{j})}^{(n)}=\,{{\mbox{Average}}}\,\left(\{{H}_{u}^{(n-1)}e(u,{b}_{j}| {c}_{i}),u \sim {{{{\mathcal{N}}}}}_{G({c}_{i})}({b}_{j}),u\ne {b}_{k}\}\right)$$

6 $${H}_{{b}_{j}}^{(n)}=\sigma \left\{{W}_{\,{{\mbox{GNN}}}}^{(n)}\cdot {{\mbox{Concat}}}\,\left[{H}_{{b}_{j}}^{(n-1)},{H}_{{{{{\mathcal{N}}}}}_{G({c}_{i})}({b}_{j})}^{(n)}\right]\right\}$$

where ${H}_{{b}_{j}}^{(n)}$ is the output vector of the node *b*_*j*_ at the *n*th layer of the GNN, and ${H}_{{b}_{j}}^{(0)}$ represents the attributes of the node *b*_*j*_ before passing to the GNN. *e*(*u*, *b*_*j*_∣*c*_*i*_) is the edge weight between node *u* and *b*_*j*_ in *G*(*c*_*i*_). ${W}_{\,{{\mathrm{GNN}}}\,}^{(n)}$ represents the weight matrix to be optimized at the *n*th layer, and *σ* is the non-linear activation function. Optionally, to take the similarity of adjacent bins in the genome into account, *b*_*j*_ can also aggregate the information from the neighbors of its adjacent bins *b*_*j*_ ± 1. We call this GNN cell-dependent because the structure of the graph depends on the cell, although the weight matrix ${W}_{\,{{\mathrm{GNN}}}\,}^{(n)}$ is shared across all cells. This cell-dependent GNN can improve the expressiveness of the NN by incorporating a large amount of single-cell information (contact maps) into the structure of the model instead of entirely relying on the embeddings of the cell nodes. The GNN is trained to reconstruct the interaction between a pair of bin nodes by using only information of themselves and their neighborhood (but not including each other). The attributes of both *b*_*j*_ and *b*_*k*_ are transformed by this cell-dependent GNN into ${\hat{b}}_{j}$ and ${\hat{b}}_{k}$, respectively, and the triplet of $({c}_{i},{\hat{b}}_{j},{\hat{b}}_{k})$ passes through the aforementioned self-attention layer to generate the final dynamic embeddings.

### Information-sharing among cells

Higashi has a unique capability for cells to share information with each other in the embedding space to enhance imputation by taking advantage of the latent correlations among cells. Specifically, we first train Higashi until convergence without the cell-dependent GNN to allow the self-attention layer to capture cell-specific information and reflect in the embeddings through back-propagation. We then calculate the pairwise distances of cell embeddings that indicate the similarities among cells. Given a hyperparameter *k*, we construct a graph *G*(*c*_*i*_) based on the contact maps of *c*_*i*_ and its *k*-nearest neighbors in the embedding space. It is crucial to clarify that, when we mention the neighbor of a cell ${{{\mathcal{N}}}}({c}_{i})$, we are referring to other cells that have small pairwise distances of embedding vectors instead of other nodes that have connections to the cell in the hypergraph. We name the contact maps of *c*_*i*_ as *M*(*c*_*i*_). The new *G*(*c*_*i*_) is constructed as the weighted sum of $M(u),u\in \{{c}_{i}\}\cup {{{\mathcal{N}}}}({c}_{i})$, where the weight is calculated based on the pairwise distance *d*(*u*, *c*_*i*_) in the embedding space—that is,

7 $$G({c}_{i}) \sim \mathop{\sum}\limits_{u}w(u,{c}_{i})M(u),\,\,u\in \{{c}_{i}\}\cup {{{\mathcal{N}}}}({c}_{i})$$

8 $$w(u,{c}_{i})\propto \,{{\mbox{exp}}}\,\left[-d(u,{c}_{i})\right]$$

Each embedding is normalized by the maximum *ℓ*^2^ norm. Note that, although contact maps of different cells are mixed in this step, we do not mix the prediction results from different cells or directly use the mixed contact maps as output. This differentiates our method from the *k*-NN-based smoothing methods fundamentally. The Higashi model is trained with only the observed interactions in each single cell, together with the interactions in cells that share overall similar structures serving as auxiliary information for synergistic prediction in a cell population.

### Loss function and training details of Higashi

The hypergraph NN in Higashi produces a score $\hat{y}$ for any triplet (*c*_*i*_, *b*_*j*_, *b*_*k*_). The NN is trained to minimize the difference between the predicted score $\hat{y}$ and the target score *y* (that is, the observations in the dataset), indicating the probability of the pairwise interaction between bin nodes *b*_*j*_ and *b*_*k*_ in cell *c*_*i*_. In Higashi, we offer several choices of loss function for scHi-C datasets with different coverage. For scHi-C datasets with relatively low sequencing depths, or the analysis resolution is high (hence, fewer reads in each genomic bin), the model is trained with a binary classification loss (cross-entropy) where the triplets corresponding to all non-zero entries in the single-cell contact maps are treated as positive samples, and the rest are considered as the negative samples (that is, *y*(*c*_*i*_, *b*_*j*_, *b*_*k*_) ∈ {0, 1}). The classification loss is:

9 $$\begin{array}{l}{{{\mbox{Loss}}}}_{{{\mathrm{class}}}}=-\mathop{\sum}\limits_{i,j,k}y({c}_{i},{b}_{j},{b}_{k}){{\mathrm{log}}}\,\hat{y}({c}_{i},{b}_{j},{b}_{k})\\\qquad\quad\qquad+\left[1-y({c}_{i},{b}_{j},{b}_{k})\right]{{\mathrm{log}}}\,\left[1-\hat{y}({c}_{i},{b}_{j},{b}_{k})\right]\end{array}$$

For datasets with relatively high sequencing depths or when the analysis resolution is low (hence, more reads in each genomic bin), we further differentiate among the non-zero values by training the model with a ranking loss, which maintains consistent ranking of predicted scores versus the continuous target scores (that is, $y({c}_{i},{b}_{j},{b}_{k})\in {\mathbb{R}}$). The ranking loss can be described as a binary classification problem aiming to identify the triplet with the larger target score in a pair of selected triplets. For simplicity, we denote two triplets as *t*_*i*_, *t*_*j*_ and the corresponding target scores as *y*(*t*_*i*_), *y*(*t*_*j*_). The ranking loss is:

10 $${l}_{ij}={\mathbb{I}}\left[y({t}_{i}) > y({t}_{j})\right]$$

11 $${p}_{ij}=\,{{\mbox{Sigmoid}}}\,\left[\hat{y}({t}_{i})-\hat{y}({t}_{j})\right]$$

12 $${{{\mbox{Loss}}}}_{{{\mathrm{rank}}}}=-\mathop{\sum}\limits_{| y({t}_{i})-y({t}_{j})| \ge \alpha }{l}_{ij}{{\mathrm{log}}}\,{p}_{ij}+(1-{l}_{ij}){{\mathrm{log}}}\,\left(1-{p}_{ij}\right)$$

where *α* defines whether the order of *y*(*t*_*i*_), *y*(*t*_*j*_) can be reliably called and is set to 2 in this work. Note that *l*_*ij*_, *p*_*ij*_ are intermediate variables used only in this definition.

Moreover, the structure of Higashi can be easily adapted to estimate a distribution for *y*(*t*_*i*_). Zero-inflated negative binomial (ZINB) distribution and its variants have been widely used in the modeling of single-cell sequencing datasets^41^. Specifically, the distribution of the read count for an entry in an scHi-C contact map can be characterized by three parameters: the mean parameter *μ*(*t*_*i*_), the dispersion parameter *θ*(*t*_*i*_) and the dropout rate *π*(*t*_*i*_). To incorporate this loss function into the Higashi framework, we change the output size of the last layer of the NN from 1 to 2. We also constrain that the dropout rate *π*(*t*_*i*_) is approximated by batch effects, total read counts in a cell and genomic distance, which are the additional features *a*(*t*_*i*_) in Higashi. The loss function for the ZINB regression can, thus, be described as:

13 $$\hat{y}({t}_{i})={[\mu ({t}_{i}),\theta ({t}_{i}),]}^{T}$$

14 $$\pi ({t}_{i})=\,{{\mbox{FC}}}\,\left[a({t}_{i})\right]$$

15 $${{{\mbox{Loss}}}}_{{{\mathrm{ZINB}}}}=-\mathop{\sum}\limits_{{t}_{i}}{{\mathrm{log}}}\,{P}_{{{\mathrm{ZINB}}}}\left[y({t}_{i})| \mu ({t}_{i}),\theta ({t}_{i}),\pi ({t}_{i})\right]$$

If the model is trained with the ZINB loss, *μ*(*t*_*i*_) is used as the imputed read count for the specific entry in the contact map. In this work, the Higashi model for sn-m3c-seq data is trained with the ZINB loss, whereas the Higashi models for the other datasets are trained with the ranking loss.

Using any of the above loss functions requires negative samples (samples with zero read count in the original datasets) in the training data. We designed an effective negative sampling approach. Specifically, at each epoch, we randomly sample a batch of triplets and make sure that these triplets do not overlap with the positive samples. To reflect the similarity of 3D genome structures of flanking genomic bins, we also exclude triplet (*c*_*i*_, *b*_*j*_, *b*_*k*_) from the negative samples if triplets such as (*c*_*i*_, *b*_*j*_ + 1, *b*_*k*_) belong to the positive samples. The number of negative samples generated for each batch is guided by the sparsity of the input data. When studying an scHi-C dataset where *s*% of the contact map entries are zeros, for a batch of *n* positive triplets, $\min \left[s/(100-s),5\right]n$ negative samples will be generated. For computational efficiency, the number of negative samples is no more than five times the number of positive samples. The model is optimized by the Adam algorithm^42^ with the learning rate of 1 × 10^−3^. The batch size is set as 192. For a dataset with multiple chromosomes, only one Higashi model is trained for all chromosomes. For different resolutions on the same dataset, separate Higashi models are trained.

### Incorporating co-assayed signals in Higashi

The unique design of Higashi allows joint modeling of co-assayed scHi-C and the corresponding one-dimensional signals (for example, from sn-m3C-seq^17^). We add an auxiliary task for Higashi by using the learned embeddings for cell nodes *c*_*i*_ to accurately reconstruct the co-assayed signals *m*_*i*_ through a multi-layer perceptron. The auxiliary loss term is added to the main loss function and optimized jointly. The model, thus, builds an integrated connection between chromatin conformation and the co-assayed signals, guiding the embedding of the scHi-C data—that is,

16 $${{{\mbox{Loss}}}}_{{{\mbox{aux}}}}={{\mbox{MSE}}}\,\left[{m}_{i},\,{{\mbox{MLP}}}\,({c}_{i})\right]$$

where MSE refers to the mean squared error between the co-assayed signals and the estimate.

### Batch effects removal during imputation

The core structure of Higashi can already implicitly remove batch effects to a certain extent during imputation. As described in Eq. (1), the final predicted probability of a triplet includes the values ${\hat{y}}_{{{\mbox{ext}}}}$ produced by feeding extra features that include features related to batch effects, such as the batch ID and the total read counts per cell. During imputation, these factors are set as constant for all cells in order to remove batch effects. The motivation for this design is to use the batch ID and total read counts to regress out the batch effects.

However, one problem that might arise is the use of contact maps with potential batch effects to construct the cell-dependent graph *G*(*c*_*i*_). This is because, when imputing cell *c*_*i*_, the *k-*nearest neighboring cells in the embedding space that contribute to its imputation are more likely from the same batch of *c*_*i*_. As a result, the batch effects in the constructed cell-dependent graph *G*(*c*_*i*_) are expected to lead to unreliable batch-correlated imputation results. To address this, we developed the following framework to explicitly remove batch effects during imputation. As described in the above section, the *k-*nearest neighboring cells in the embedding space could contribute to the imputation by using the weighted average of the corresponding contact maps to construct the cell-dependent graph *G*(*c*_*i*_). Motivated by the mutual nearest neighbor method that is widely adopted in scRNA-seq analysis for batch effect removal^43^, we add constraints for the selection of neighboring cells that will involve in the imputation. When imputing a cell *i* from an scHi-C dataset with *N* batches, we require that the *k-*nearest neighbors contributing to the imputation process must be evenly distributed across *N* batches. In cases where there is no exact division ⌈*k/N*⌉ cells will be sampled from each batch based on their distance to cell *i* in the embedding space. Next, *k* cells will be randomly selected and serve as the final set of neighboring cells to contribute to imputation. Note that this new neighborhood construction mechanism will be carried out dynamically after every epoch of the training process of Higashi to improve the robustness of the imputation and the random sampling process. By incorporating this mechanism into Higashi, *G*(*c*_*i*_) will have similar distribution across different batches. The Higashi model is now able to regress out the batch effects with the batch ID and read count information. During imputation, the batch-effects-related features will be set as constant from the input to recover batch-effect-corrected contact maps.

### Variability of compartmentalization and TAD-like boundaries

In Higashi, we developed strategies for reliable analysis of 3D genome features in different scales across the cell population. We developed a method to calculate continuous compartment scores for the imputed single-cell contact maps such that these scores are directly comparable across different cells in the population to assess variability (Supplementary Note A.5). For single-cell TAD-like domain boundary analysis, we developed a calibration method using an optimization scheme based on insulation scores to achieve comparative analysis of domain boundary variability from single cells (Supplementary Notes A.7 and A.8). These algorithms greatly enhance the analysis of variable multiscale 3D genome structures at single-cell resolution.

### Visualization tool for integrative scHi-C analysis

In Higashi, we developed a visualization tool that allows interactive navigation of the scHi-C analysis results. Our tool enables the navigation of the embedding vectors and the imputed contact maps from Higashi in a user-friendly interface. Users can select individual cells or a group of cells of interest in the embedding space and explore the corresponding single-cell or pooled contact maps. Supplementary Fig. 28 shows a screenshot of the visualization tool. See the GitHub repository of Higashi for detailed documentation of this visualization tool: https://github.com/ma-compbio/Higashi.

### Reporting Summary

Further information on research design is available in the Nature Research Reporting Summary linked to this article.

## Online content

Any methods, additional references, Nature Research reporting summaries, source data, extended data, supplementary information, acknowledgements, peer review information; details of author contributions and competing interests; and statements of data and code availability are available at 10.1038/s41587-021-01034-y.

## Supplementary information

Supplementary Information Supplementary Notes, Supplementary Tables 1–5 and Supplementary Figs. 1–28

Reporting Summary

## Data Availability

We used the following publicly available datasets: sci-Hi-C of four cell lines from Ramani et al.^14^ (GEO: GSE84920); scHi-C of mouse embryonic stem cells from Nagano et al.^15^ (GEO: GSE94489); sci-Hi-C of five cell lines from Kim et al.^20^ (4DN Data Portal: 4DNES4D5MWEZ, 4DNESUE2NSGS, 4DNESIKGI39T, 4DNES1BK1RMQ and 4DNESTVIP977); scHi-C of WTC-11 iPSC cell line (4DN Data Portal: 4DNESF829JOW and 4DNESJQ4RXY5); sn-m3c-seq of human prefrontal cortex cells from Lee et al.^17^ (GEO: GSE130711); Bulk Hi-C of WTC-11 (4DN Data Portal: 4DNESPDEZNWX and 4DNESJ7S5NDJ); scRNA-seq of WTC-11 from Friedman et al.^26^ (EMBL-EBI: E-MTAB-6268); CTCF ChIA-PET of WTC-11 (4DN Data Portal: 4DNES8MZ76GP); and scRNA-seq of multiple cortical areas of the human brain from the Allen Brain map^37^: https://portal.brain-map.org/atlases-and-data/rnaseq/human-multiple-cortical-areas-smart-seq.

## Code availability

The source code of Higashi can be accessed at https://github.com/ma-compbio/Higashi. The detailed code dependency list of Higashi can be found at the GitHub page, which includes Python (3.7.9), numpy (1.19.2), pytorch (1.4.0) and scikit-learn (0.23.2).
