# Author Correction: Multiscale and integrative single-cell Hi-C analysis with Higashi

**DOI:** 10.1038/s41587-022-01263-9

**Published:** 2022-03-03

**Authors:** Ruochi Zhang, Tianming Zhou, Jian Ma

**Affiliations:** Computational Biology Department, School of Computer Science, Carnegie Mellon University, Pittsburgh, PA USA

**Keywords:** Machine learning, Epigenomics

Correction to: *Nature Biotechnology* 10.1038/s41587-021-01034-y, published online 11 October 2021.

In the version of this article initially published, there were composition errors in the captions for Fig. 3b and Supplementary Fig. 15a,e,h. In each lettered caption, there were two sentences describing the number of genes having stable and dynamic single-cell compartment scores and their average transcription activity variability, respectively. The values in each caption were initially reversed for stable and dynamic compartment scores and variability. In Fig. 3b, in the corrected text now reading “There are 5,071 genes that have stable single-cell compartment scores, with average transcription activity variability equal to 77.4. There are 5,075 genes that have dynamic single-cell compartment scores, with average transcription activity variability equal to 86.0,” the values 5,071, 77.4 and 5,075, 86.0 replaced the original order of 5.075, 86.0 and 5,071, 77.4. The changes have been made to the online version of the article and the Supplementary information has been updated.

