# Supplementary material for: Multiscale and integrative single-cell Hi-C analysis with Higashi
Source: Nat Biotechnol. 2021 Oct 11;40(2):254–61. doi: 10.1038/s41587-021-01034-y (PMC8843812; doi:10.1038/s41587-021-01034-y)
Supplement: Supplementary file 1 — Supplementary Notes, Supplementary Tables 1–5 and Supplementary Figs. 1–28 [file 41587_2021_1034_MOESM1_ESM.pdf]

nature portfolio

https://doi.org/10.1038/s41587-021-01034-y

Supplementary information

Multiscale and integrative single-cell Hi-C analysis with Higashi

In the format provided by the authors and unedited

Multiscale and integrative single-cell Hi-C analysis with Higashi

(SUPPLEMENTARY INFORMATION)

A Supplementary Notes

A.1 Evaluation procedure of the embedding vectors

We quantified the effectiveness of the embeddings from different methods (Higashi, HiCRep/MDS [1], scHiCluster [2] and LDA [3]) with various supervised and unsupervised evaluation tasks. We kept the embedding dimensions and the resolution of the input scHi-C contact maps the same for all methods to make fair comparisons. The rest hyperparameters were kept as the default or the ones that were used in the original study for a specific dataset. For instance, for LDA, we set the maximum distance between a locus pair as 10Mb for the Ramani et al. and 4DN-sci-Hi-C datasets, and 25Mb for the Nagano et al. dataset, which was the same parameter setting in [3]. For sn-m3c-seq dataset [4], we also included the embeddings generated from the CG methylation (mCG) profile using Scanorama [5] for comparisons. The embedding dimension is kept the same as the other embeddings based on scHi-C for direct comparisons. Note that the dimension of mCG embeddings is different from what is used in [4] to annotate the cell types. For the supervised setting, we trained a logistic regression classifier with 10% of the cells as training data and the remaining 90% cells as testing. The supervised setting is designed to mimic the common practice of confidently annotating a small fraction of cells with high coverage based on prior knowledge (e.g., marker genes). Because the value range of embeddings produced by different methods vary, which would affect the convergence as well as the numerical stability, we applied Z-score normalization before training the logistic regression classifier. Note that linear transformation on the embeddings would not change the solution of a linear classifier such as logistic regression. It only affects the convergence rate and the numerical stability of the algorithm for solving the optimal weights of the classifier. The performance for this multi-class classification task was measured by Micro-F1 and Macro-F1 scores. For the unsupervised setting, we applied K-means clustering on the datasets with discrete cell states (such as the 4DN sci-Hi-C and Ramani et al. datasets used in this work) and calculated the Adjusted Rand Index (ARI) of the predicted labels against the cell types. Because Z-score normalization would change the weight of each dimension when calculating euclidean distances in K-means clustering, we did not apply Z-score normalization to each dimension separately but rather scaled the embeddings across different dimensions altogether with Z-score. For datasets with continuous cell states (the Nagano et al. dataset used in this work that represents states of the continuous cell cycle), we used a metric called ACROC (Average Circular ROC) proposed in [1] to evaluate the consistency of embedding vectors with circulatory states. All metrics used in this evaluation range from 0 to 1 with a larger number indicating better performance. For comparison, all methods in our evaluation generated 64-dimensional embeddings for all evaluation metrics except ACROC since it is defined in the two-

dimensional space. When calculating the ACROC score, both scHiCluster and Higashi embeddings were projected to the two-dimensional space with PCA, whereas HiCRep/MDS uses MDS as a dimension reduction method inherently. The embeddings produced by LDA were projected to the two-dimensional space with UMAP as it is the preferred dimension reduction method suggested in [3].

A.2 Discussion on the architecture of the hypergraph neural network

One of the unique designs of the hypergraph neural network in Higashi is the static and dynamic embedding pairs. Here, we discuss the rationale of the model design (see the description of the neural network architecture in Methods). As described in the main text, the "static embedding" s_i remains the same for node v_i independent to the given triplet, reflecting the general topological properties of a node in the given hypergraph. The static embedding can be used for tasks such as clustering and classification on the node level. The "dynamic embedding" d_i depends on all the node features within this triplet and reflects the specific properties of a node v_i in a particular hyperedge. With this design, the network aims to establish the correlation of the average "distance" of the static/dynamic embedding pairs with the probability of a group of nodes forming a hyperedge. The assumption is that for a node v_i, the more similar its specific properties in a node tuple (dynamic embeddings) is to its general topological properties in the given hypergraph (static embeddings), the more likely v_i can form a hyperedge with the rest of nodes in that node tuple. In addition, the self-attention mechanism used in the hypergraph neural network here is different from the standard self-attention introduced in [6]. Specifically, the self-similarity term α_{ii} is excluded. As a result, the dynamic embeddings for a node v_i given a node tuple is the weighted average of features from other nodes in this node tuple (with non-linear transformation). Thus, the distance between static and dynamic embeddings reflects how well the static embedding of each node can be approximated by the features of other nodes within that tuple.

In [7], we performed analysis on the trained hypergraph neural network and observed that the dynamic embedding of a node given a hyperedge can indeed approximate its static embedding better than its dynamic embedding when given a random node tuple. Ablation studies in [7] also showed that this static/dynamic embedding setting can achieve better and more stable performance over multiple tasks on several benchmark datasets than models with other structures (e.g., simply using dynamic embeddings to predict the probability of a node tuple forming a hyperedge).

A.3 Implementation details and runtime of Higashi

The implementation of Higashi is optimized in several aspects. Here, we describe the implementation details of Higashi and its runtime.

Runtime optimization for getting node features via graph neural network. A cell-dependent graph neural network (cd-GNN) is utilized in Higashi to enhance the expressiveness of the model. Specifically, for each single cell, a graph is constructed based on the original sparse contact maps of itself and optionally its k nearest neighbors in the embedding space. In practice, there are two ways of implementing a graph neural network. The first one is the Graph-SAGE [8]-like models where the operation unit is a node with its neighbors. The second one is the graph convolutional network [9]-like models where the operation unit is a whole graph. The Graph-SAGE-like models are typically used for large graphs where only a part of the nodes will appear in one training batch, which would be computationally more efficient to only propagating information among the nodes used in the training batch and their neighbors. In the Methods section, when introducing our cell-dependent graph neural network, we used the notation of the

Graph-SAGE-like models. On the other hand, the graph convolutional network-like models are typically used for one small graph or a collection of small graphs, and thus passing information across all nodes in a graph would be possible. Specifically, the process can be described as:

$$H_B^{(n)} = \sigma \left\{ W_{\text{GNN}}^{(n)} \cdot \text{Concat} \left[E_{G(c_i)} H_B^{(n-1)}, H_B^{(n-1)} \right] \right\}$$

where $H_B^{(n)}$ is the output vectors for all bin nodes at the n-th layer of the GNN, $E_{G(c_i)}$ is the adjacency matrix of graph $G(c_i)$, $W_{\text{GNN}}^{(n)}$ represents the weight matrix to be optimized, and σ is the non-linear activation function. In Higashi, during training, random hyperedges that correspond to scHi-C contacts from different cells are used as positives. In cd-GNN, these hyperedges correspond to nodes of different graphs. Thus, propagating information on all cell-dependent graphs would be inefficient and we use the Graph-SAGE-like method in the training process. However, during imputation, we control that the prediction for hyperedges is done for one chromosome from one cell at a time such that all node triplets in a batch come from the same cell and share the same cell-dependent graph. Moreover, the same node typically occurs many times in the test batches because the samples to be predicted are all possible combinations of one cell node and two bin nodes. Therefore, before imputing a specific cell c_i, we use the trained cd-GNN to propagate information across the graph $G(c_i)$ in a graph convolutional network-like manner and store the results. When making predictions on the node triplets, the embeddings for bin nodes directly come from the stored results instead of passing through the cd-GNN again for better computational efficiency.

Memory consumption optimization for constructing cell-dependent graph. Another optimization we implemented on the cd-GNN relates to how we calculate the cell-dependent graph using neighboring cell information. We provide two options in Higashi: the fast mode and the memory saving mode. In the fast mode, all cell-dependent graphs are calculated once and stored in the memory. However, it would require storing denser matrices (in the worst case, k times denser than the original sparse contact matrices) in the memory. To address this, the memory saving mode is provided, where the corresponding rows to be used in a training batch are dynamically calculated. This drastically reduces the burden of memory at the cost of longer training time.

Runtime optimization through parallelization. Additionally, the training and imputation stages of Higashi is also highly parallelizable. During the training stage, the most time-consuming part is generating negative samples (zero entries in the sparse contact maps) and fetching the node and its neighbors in the cell-dependent graph. However, both operations can be distributed to multiple CPU cores. Specifically, hyperedges are sampled and grouped into batches. Each batch will be sent to different CPU cores to generate the negative samples and fetch neighbors in the cell dependent graph. The batches that are processed first will proceed to the training of Higashi model. During imputation, multiple copies of Higashi model can be launched and distributed to multiple GPU cards to impute different cells and then the prediction results will be merged.

All analyses in this work were carried out on a Linux machine with 8 NVIDIA RTX 2080 Ti GPU cards, a 16-core Intel Xeon Silver 4110 CPU, and 252GB memory. As mentioned in the Methods section, the batch size is set as 192. Since the number of hyperedges varies across different datasets, we use the operation time per 1000 batches as the unit for measuring the runtime. For simplicity, we refer to 1000 batches as one epoch, which is different from the conventional definition where one iteration over the whole training dataset is one epoch. For the reported runtime, the Higashi program is set to use all available CPU cores and one GPU card during training. It is also set to not use parallel imputation

although for smaller datasets one GPU card could fit multiple Higashi models. The runtime of the core operations of Higashi is reported in Table S5.

A.4 Simulation data generation for the evaluation of scHi-C imputation

We describe how we generated the simulated scHi-C data for the evaluation of the contact map imputation. For 11,631 imaged chromosome regions (chr21:34.6Mb-37.1Mb) in [10], we first turned the 3D coordinates of sequential 30Kb bins into a spatial distance map of size 83×83 for each cell. As shown in [10], the inverse spatial distance between a pair of genomic loci and the number of reads in the corresponding entry of the Hi-C contact map have a high linear correlation. We thus used the inverse spatial distance map as the ground truth (referred to as the probability map) and randomly sampled reads with replacement in proportion to the value of each entry in the probability map. Because the diagonal entries of the distance map are all zeros, which leads to error when trying to calculate the inverse, we directly set all diagonal entries of the probability matrix to zero and excluded the first diagonal for all similarity measurements.

By the law of large numbers, the contact map constructed based on the sampled reads (referred to as sampled contact frequency map) would converge almost surely to the ground truth when the number of sampled reads n goes to infinity. We first evaluated the similarity between the sampled contact frequency map and the ground truth across different read numbers (Fig. S7). As expected, all similarity measurements increase when the read number increases. Moreover, when calculating the distance stratified correlation score [11], the correlation scores for long-range interactions are always lower. We observed that, only when n is greater than 50,000, the median of both the Pearson and Spearman correlation scores between the simulated contact frequency maps and the ground truth are higher than 0.7, which highlights the significance of contact map imputation. To show the robustness of the method over the sampled read number, we simulated contact maps with 4 groups of read numbers, including 100 to 250, 250 to 500, 500 to 1,000, and 1,000 to 2,500.

We also generated simulated scHi-C datasets based on a more recent 3D genome imaging dataset (3,029 chromosomes, chr2) from [12]. Note that this imaging dataset was obtained by labeling and imaging 50Kb segments at intervals of 250Kb. We first approximated the spatial coordinate of a 1Mb segment by averaging the coordinates of the four 50Kb segments within it and then followed the same procedure as above to obtain a spatial distance map of size 243×243. The probability map was again obtained by calculating the inverse spatial distance map. To reflect the proximity ligation procedure in scHi-C experiments, we set all the entries in the probability map with a distance greater than 2,000 nm in the distance map to zero. The sampled contact frequency maps were then generated based on the probability map. To reflect various coverage of different scHi-C technologies, we calculated the contacts per cell for various scHi-C datasets on chr2 at 1Mb resolution (Table S3) and then chose the read numbers for the simulation data as 250 to 500, 500 to 1,000, 1,000 to 2,500, and 2,500 to 5,000.

In addition, we generated simulated scHi-C datasets by downsampling the WTC-11 scHi-C on chr1 at 1Mb resolution. At a given downsampling rate $1/s$, we randomly selected $1/s$ of all the sequencing reads assigned to a specific cell and generated a sampled contact frequency map. This process was repeated for s times for each cell in the original dataset. We chose the downsampling rate based on the contacts per cell in the existing scHi-C datasets on chr1 at 1Mb resolution (Table S4). The downsampling rates used in this study include 1/3, 1/5, 1/10, and 1/15.

For a fair comparison and also for the visualization purpose, all contact maps were quantile-normalized

such that the ground truth and the imputation results from different methods would have similar value range and distribution. For the imaging-data based simulation, all the imputation results were evaluated with similarity metrics between the imputed contact map and the ground truth derived from the imaging data. The similarity metrics used include the Pearson correlation score, the Spearman correlation score, and also HiCRep [11], which is a stratum-adjusted correlation coefficient score for measuring the similarity of two Hi-C matrices. For downsampling based scHi-C simulation, besides the global Pearson and Spearman correlation scores, we also visualized the distance stratified correlation scores (measured by the Pearson and Spearman for each stratum). Since the full-coverage contact maps (used as the ground truth) are still sparse and noisy, it is sometimes numerically unstable to calculate the correlation score for long-distance interactions. We therefore only included the distance stratified correlation score for the first 15 strata as the full-coverage scHi-C contact maps are still relatively dense in these regions. For the rest of the regions in the contact maps, we calculated the AUPR score with the existence of long-range interactions in the full-coverage contact maps as labels and the values of the corresponding entries in the enhanced contact map as the predicted probability scores.

A.5 A/B compartment score annotation for scHi-C dataset

We designed an approach for A/B compartment score annotation based on the widely-used method proposed in [13]. In the original compartment calling method, the Hi-C contact map is normalized and transformed into a Pearson correlation matrix. The sign of the first principal component (referred to as PC1 for simplicity) of this correlation matrix is then used to define A/B compartments. To generate A/B compartment scores for a scHi-C dataset, the most straightforward way would be to apply the original algorithm to each contact map. However, to compare the A/B compartment annotations across the cell population, this method has several major limitations. First, the original method uses zero as cut-off to binarize the PC1 to A/B compartment label. However, when making comparisons across the cell population, especially within the same cell type, binary labels are not sufficiently expressive to characterize detailed variations. Indeed, subtle changes of compartmentalization that reflect nuclear spatial positioning could lead to important genome function differences [14–17]. Second, the continuous PC1 scores obtained from individual cells are not directly comparable by definition because the PC1s of different cells are in different lower-dimensional spaces.

To address this, we developed a new method where the first two steps, i.e., normalization and transformation into Pearson correlation matrices, remain the same for each single cell. However, instead of performing PCA on each individual Pearson correlation matrix, we apply PCA once on the Pearson correlation matrix from the pooled scHi-C $R_{pool} \in \mathbb{R}^{N\times N}$ and save the PCA projection matrix. We then use this bulk projection matrix to transform the single-cell Pearson correlation matrices R_i into continuous one dimensional vectors c_i.

$$\hat{R}_{pool} = R_{pool} - \bar{R}_{pool} \quad \text{(Centering by columns)} \tag{1}$$

$$\hat{R}_i = R_i - \bar{R}_i \quad \text{(Centering by columns)} \tag{2}$$

$$\hat{R}_{pool} = USV^T \quad \text{(SVD decomposition)} \tag{3}$$

$$c_i = \hat{R}_i V^{(1)} \quad \text{(Projection)} \tag{4}$$

where $V^{(1)}$ stands for the first column of the projection matrix V. Although technically these one dimensional vectors are not the first principal components of each single-cell contact map, for simplicity,

we still refer to them as PC1s for each cell. These PC1s in individual cells are continuous and sensitive to reflect the subtle variability of compartment shifts, enabling direct comparison of compartment scores across individual cells.

A.6 Calculation of transcriptional variability

In scRNA-seq data, there is an inherent connection between the mean and standard deviation of read count [18]. Therefore, the metrics such as variance or standard deviation would lead to the observation that the highly expressed genes also appear to be the more variable ones. To mitigate this, we quantified the transcriptional variability with two approaches. In the first approach, we use a commonly used metric, coefficient of variation (CV), to balance the correlation between mean and standard deviation [19]. Due to the dropout events of the scRNA-seq data, direct calculation of mean and standard deviation will also be less stable on the sparse scRNA-seq data. We thus utilized MAGIC [20] to impute the scRNA-seq data after normalization to address the sparsity issue. The CV is calculated as the ratio between the standard deviation and the mean for each gene on the imputed scRNA-seq data. In the second approach, we used a recently developed method for variance stabilization of scRNA-seq data [18], which takes into account both the dropout events and the correlation between mean and standard deviation. We applied the method to the scRNA-seq data before imputation. The residual variance is then used as the measurement for the transcriptional variability.

A.7 TAD-like domain boundary identification for scHi-C dataset

We used the insulation score based method for calling TAD-like boundaries [21]. We denote the contact map as M and the window size as w. The insulation score s at position x is calculated as:

$$s(x) = \text{inter}(x)/\text{intra}(x) \tag{5}$$

$$\text{inter}(x) = \sum_{x-w\leq i\leq x, x\leq j\leq x+w} M_{ij} \tag{6}$$

$$\text{intra}(x) = \sum_{x-w\leq i\leq x+w, x-w\leq j\leq x+w} M_{ij} \tag{7}$$

A high insulation score indicates that the flanking regions across this genomic locus highly interact with each other, suggesting that this locus is in the middle of a TAD-like domain. On the contrary, a low insulation score indicates that the neighboring loci are well separated and this position is likely a domain boundary. Therefore, the domain boundaries are identified as the local minima of the insulation scores. We used a window size of 2Mb for calculating the insulation scores and a window size of 500Kb for identifying the local minima in this work.

A.8 TAD-like domain boundary calibration for scHi-C dataset

To quantify the cell-to-cell variability of TAD-like domain boundaries in the cell population, it is crucial to have a reliable method for domain boundary comparisons in single cells. Although methods have been developed for comparing a pair or a small number of TAD sets (e.g., [22, 23]), it remains computational impractical to directly apply them to scHi-C datasets with hundreds or even thousands of cells due to their poor scalability. We developed a new computational strategy for calibrating domain boundaries called from the scHi-C. Specifically, we first identify a set of domain boundaries shared across the cell

population. The size of this shared boundaries is predefined as K, which is chosen as 2 times the number of domain boundaries identified in the pooled scHi-C contact maps in this work. During optimization (see below), shared boundaries that are too close to each other (≤ 1 bin) will be automatically merged. Each single-cell domain boundary will then be assigned to one of these shared boundaries. In other words, the domain boundaries for each cell will be represented as a subset of this shared boundary set. The assignment and the shared boundary set are optimized such that the calibrated ones are similar to the original ones. Together, this can be defined as:

$$\begin{aligned} \text{minimize}_{B_i^*} \quad & \sum_{i,j} \text{dis}\left(b_{i,j}^*, b_{i,j}\right) \\ \text{s.t.} \quad & b_{i,j} \in B_i \\ & b_{i,j}^* \in B_i^* \\ & B_i^* \subseteq B^* \\ & |B^*| = K, \end{aligned} \tag{8}$$

where B_i and B_i^* represent the single-cell domain boundaries before and after calibration for cell i and $\text{dis}()$ represents the distance measurement of two boundaries. Each shared boundary thus acts as an anchor point such that the variable single-cell domain boundaries associated with it can be analyzed systematically across the cell population. Fig. S16a shows examples of the single-cell domain boundaries before and after calibration. We did not use the domain boundaries called from the pooled scHi-C to specifically allow the algorithm to identify boundaries that are enriched in a group of cells but not strong enough to be captured in the pooled scHi-C. On the other hand, using pooled domain boundaries as initialization for B^* would accelerate convergence. As mentioned, we chose K as 2 times the number of boundaries called from the pooled scHi-C in this work. When initializing B^*, half of them are initialized as the pooled domain boundaries while the rest are randomly spaced across the genome.

This problem setting shares similarities with the well-known K-means clustering. If we define the $\text{dis}()$ function as the Euclidean distance, it would be equivalent to K-means clustering. However, the Euclidean distance based on the genomic coordinate is not appropriate to quantify how well one domain boundary approximates another one (see example in Fig. S16b). When using genomic distance as the evaluation metric, both shared domain boundaries have exactly the same distance to the single-cell domain boundary before calibration. However, the left one is preferred as it has a relatively smaller change in the insulation score. For the similar reason, simply using the distance between insulation scores at the domain boundaries is also not desired.

We therefore developed a new metric called "cumulative insulation score" to address this. As illustrated in Fig. S16b, this metric is calculated as the area between the insulation score curve and the horizontal line at the minimum insulation score within the given region. This new metric penalizes the assignment if the assigned domain boundary either has a large genomic distance or has very different insulation scores to the original one. With this approach, in both situations mentioned above, the single-cell boundary will be assigned to the correct shared boundary with a smaller cumulative insulation score.

Together, we alternate between optimizing the shared boundary set $B^* = \{b_k^*\}$ and optimizing the

assignment $c_{i,j}$ until convergence, i.e.,

$$\text{assignment step: } c_{i,j} = \arg\min_{k} \text{dis}(b_k^*, b_{i,j}) \tag{9}$$

$$\text{update step: } b_k^* = \arg\min_{b_k^*} \sum_{i,j} \mathbb{I}(c_{i,j} = k)\text{dis}(b_k^*, b_{i,j}) \tag{10}$$

where $\text{dis}(b_k^*, b_{i,j})$ is the "cumulative insulation score" between the shared boundary b_k^* and the jth boundary in cell i. Eventually those shared boundaries with less than 5 assigned single cell boundaries are removed for subsequent analysis.

A.9 Identifying differentially expressed genes during WTC-11 differentiation

To probe the connection between variable TAD-like domain boundaries and gene function, we investigated the differentially expressed genes (DEGs) within those boundaries related to differentiation based on scRNA-seq data [24]. Specifically, in [24], scRNA-seq was acquired from cells at 5 differentiation stages from WTC-11 cells, including pluripotency, germ layer specification, cardiac cells progenitor, committed cardiac cells, and definitive cardiac cells. To filter out low-quality cells, we only kept cells with 500 to 4,000 read counts and less than 10 percent of mitochondrial counts using Seurat [25, 26]. We identified DEGs between pluripotency states and each of the other 4 differentiated stages using Seurat with default parameters.

A.10 Sensitivity analysis of hyperparameters in Higashi

Here, we discuss how we evaluated the robustness of Higashi to the hyperparameters in the model. We first tested Higashi's embeddings with regard to the embedding dimensions based on the Ramani et al. dataset. We trained Higashi as well as the baseline methods, scHiCluster [2], HiCRep/MDS [1], and LDA [3], to produce embeddings with different dimensions. The details for the evaluation procedure can be found in Supplementary Notes A.1. As shown in Fig. S5a, among all four methods, Higashi achieves the highest scores and is the most robust to the embedding dimensions. Under the supervised setting, the embedding vectors produced by scHiCluster, HiCRep/MDS and LDA have a strong overfitting problem where the logistic regression classifier trained on 10% of the embeddings do not generalize well to the rest of the cells. However, the embeddings of Higashi do not encounter this problem with stable Micro-F1 and Macro-F1 scores across different embedding dimensions. Under the unsupervised setting, Higashi performs consistently well across all embedding dimensions. The performance quantified by ARI becomes even better for higher dimensional embeddings. The other three baseline methods achieve relatively stable ARI scores across different embedding dimensions as compared to their Micro-F1 and Macro-F1 scores in the supervised setting, but they either perform worse in general or are overall not as robust as Higashi. Notably, we observed that the method LDA cannot achieve reasonable clustering without the utilization of UMAP (marked with LDA (UMAP) in Fig. S5a).

The second hyperparameter that we evaluated in Higashi is the number of nearest neighbors in the embedding space that coordinate the data imputation for a specific cell. As mentioned, one unique novelty of Higashi with the hypergraph formulation is the ability to allow information to be shared across different cells, which is controlled by the hyperparameter k. This parameter can be chosen based on the sparsity of the contact map to be imputed and also the number of cells within the dataset. For instance, if the coverage of the dataset is very limited, it is appropriate to have a larger k to enhance the imputation.

Moreover, if the number of cells within the dataset is large, a higher k would also be desired because there is a greater probability that a cell in such a population shares a similar 3D genome structure with other cells. In order to evaluate if Higashi is robust to the choice of k, we repeated the simulation evaluation based on the data from [10] with $k = 0$, 2, 4, 6 and ∞ (using all other cells within the experiment), respectively. As shown in Fig. S5b, across different sampled read numbers, Higashi(2), Higashi(4), and Higashi(6) always achieve higher similarity scores with the ground truth compared with Higashi(0) and the baseline models. The difference in the performance among Higashi(2), Higashi(4), and Higashi(6) is minimal, strongly suggesting the robustness of Higashi to the hyperparameter k. We also included the extreme case where there is no limit on the number of neighboring cells involved in the imputation. We found that this will lead to a slight decrease in imputation accuracy (Fig. S5b). However, as compared to Higashi(0) and other baselines, Higashi(∞) still achieves higher or comparable performance.

We also repeated this evaluation on the simulated data based on the imaging data of the whole chr2 from [12]. Similar to what we observed on the simulation data based on [10], the advantages of Higashi(2), Higashi(4), and Higashi(6) over other methods are significant with very little variance among them. Higashi(∞) decreases the imputation accuracy under certain evaluation metrics but still performs reasonably well as compared to the baseline models (Fig. S5c).

Taken together, we demonstrated that the unique design in Higashi to allow information to be shared between neighboring cells in the embedding space consistently improves data imputation with robust performance. The choice of hyperparameter k affects the imputation results only slightly in Higashi with consistent advantages over other methods, further suggesting the robustness of the method.

A.11 Comparison with 3D structure modeling for imputing sparse contact maps

Calculating self-consistent 3D genome structures from scHi-C is potentially an alternative approach for imputing missing contacts in the original scHi-C dataset [27, 28]. Here, to further assess Higahi's advantage, we compared the imputation results from Higashi with the 3D structure modeling results from the Dip-C package [28]. We obtained the 3D modeling structures and the corresponding interaction pairs of chromosome 1 in 14 GM12878 cells with 3D structure models from [28]. The Dip-C package runs 3D modeling on haplotype resolved interaction pairs and would result in 28 single chromosome 3D structures at the 500Kb resolution. Note that the 500Kb resolution is chosen to make sure that the 3D structure modeling algorithm could produce 3D coordinates for all non-centromere genomic bins at full coverage. We found that 3D structure modeling at higher resolutions would skip bins without enough Hi-C contacts.

We turned the 3D coordinates of 500Kb genomic bins in each 3D structure into a pairwise distance map, the inverse of which would be referred to as the contact probability map. Next, we averaged two diploid contact probability maps to obtain the single-cell contact probability map, which would be regarded as the imputation results from the 3D modeling approach. Also, we used the read pairs of these 14 GM12878 cells as input for Higashi to obtain the imputed single-cell contact maps for these cells. Because there are only 14 cells in the dataset, we only included the Higashi results with $k = 0$. Before computing the similarity, all maps are quantile-normalized to [0,1] to match the value-distribution of contact maps generated through different approaches. We quantified the similarity between the imputation results from the 3D structure modeling approach and Higashi with HiCRep ($\leq$100Mb), Pearson correlation, and Spearman correlation. The similarity scores are high (Fig. S11 median $>$0.85 on all three metrics), indicating the overall consistency of the Higashi imputation and the 3D structure modeling

result for high coverage datasets.

Next, we evaluated the robustness of Higashi and the 3D structure modeling approach to the sequencing depth by downsampling the interaction pairs. We downsampled the datasets to 25%, 10%, 5%, and 1% of the original read coverage. Note that the median number of contacts per cell of both sn-m3c-seq and WTC-11 scHi-C datasets used in this work is around 25% of the original coverage of the Dip-C GM12878 dataset, while the median number of contacts per cell of a typical sci-Hi-C dataset is around 1% of the original Dip-C GM12878 coverage (Table S4). We quantified the robustness of these two different methods based on the similarity between the imputation results from the downsampled dataset and the 3D structure modeling result on the full-coverage dataset. As shown in Fig. S11, we found that when downsampling the Dip-C dataset to the coverage of a typical scHi-C dataset, 3D structure modeling becomes less reliable and fails to model the coordinates of multiple bins, leading to much lower similarity scores as compared to the imputation results of Higashi.

Taken together, this analysis demonstrates that the imputation of Higashi is consistent with the 3D structure modeling results of a self-consistent chromatin structure on a high coverage dataset. We remark that the “ground truth” used in this comparison is the 3D structure modeling at full coverage Dip-C GM12878 dataset where the model itself contains potential bias and uncertainty. Nevertheless, the comparison with 3D structure modeling at different coverage demonstrates that the imputation of Higashi is more robust to the sequencing depth of the input data. Additionally, as mentioned above, the analysis in this section was done at the resolution of 500Kb to make sure that 3D structure modeling could produce 3D coordinates for all non-centromere genomic bins. In practice, 3D structure modeling at higher resolution has a very high requirement for sequencing depth, which does not work for the vast majority of scHi-C data. As a result, in [29], even with an average coverage of 401k contacts per cell (minimum=101k), only 43% of the cells were used to generate reliable 3D structure models. Therefore, the robustness of Higashi as demonstrated in this section makes it a much more generalizable method for studying scHi-C datasets of various sequencing depths.

A.12 Comparisons between the pooled imputed contact maps and the bulk Hi-C

To further evaluate the imputation of Higashi at a population level, we made comparisons between the pooled scHi-C contact maps and the bulk Hi-C of the same cell type. We did the evaluation on three cell types in 4DN sci-Hi-C dataset with a sufficient number of cells: GM12878, HAP1, and H1ESC. We obtained the bulk Hi-C of these three cell types from 4DN Data Portal (GM12878: 4DNES3JX38V5, HAP1: 4DNESUTEOMGQ, H1ESC: 4DNESRJ8KV4Q). We imputed the scHi-C contact maps at different resolutions (1Mb, 500Kb, and 250Kb) using Higashi and scHiCluster. After imputation, we randomly selected different numbers of cells, ranging from 1 to 1,000, pooled the contact maps of the selected cells, and calculated the similarity between the pooled scHi-C contact maps and the contact map from the bulk Hi-C. We used three metrics to quantify the similarity: the HiCRep (contacts within 100Mb are considered), global Pearson correlation, and global Spearman correlation. The similarities of different chromosomes are calculated individually and then weighted averaged by the length of the chromosome, which is the same method used in HiCRep for calculating genome-wide similarity scores. We repeated this process (i.e., random selection, pooling, and calculating similarity process) for 30 times for a given cell type, and aggregated results from all three cell types for analysis and visualization.

As expected, the similarity between the pooled scHi-C contact maps and bulk Hi-C increases with the number of the cells being pooled. The similarity score also decreases when analyzing at a finer

resolution (Fig. S12). All imputation methods would improve the similarity between the pooled scHi-C contact maps and the bulk Hi-C contact maps. Such improvement is more obvious when fewer cells are pooled or when analyzing the dataset at a finer resolution (Fig. S12). Moreover, as shown in Fig. S12, the pooled imputed scHi-C contact maps by Higashi consistently achieve higher similarity than the pooled imputed scHi-C contact maps by scHiCluster. The advantage is more obvious when using HiCRep as the similarity measurement and when a smaller number of cells are used. It must be noted that the Higashi(4) results at a given number of pooled cells potentially contain more information besides the selected cells. By design, Higashi(4) aggregates information of neighboring cells in the embedding space, and thus contains more information.

Taken together, the evaluation shows that the pooled imputed scHi-C contact maps by Higashi are consistent with the bulk Hi-C contact maps of the same cell type. The advantage of Higashi on imputing the scHi-C contact maps can be observed at the population level as well, achieving higher similarity with the bulk Hi-C, especially when a smaller number of cells are used. However, it is important to emphasize that the main purpose of Higashi is to impute the scHi-C contact maps such that the multiscale 3D genome features, including compartments and TADs, and their variability can be revealed at single-cell resolution (as we demonstrate in the main text), which is different from the goal of enhancing the overall quality of the pooled scHi-C contact maps or the bulk Hi-C with low sequencing depths.

A.13 Analysis of CTCF binding near single-cell TAD-like domain boundaries

From earlier work using bulk Hi-C, it is known that TAD boundaries are typically enriched with CTCF binding [30]. We sought to reveal the connection between CTCF binding and TAD-like domain boundaries from scHi-C. Based on the occurrence frequencies of the shared TAD-like domain boundaries in the cell population, we classified the shared boundaries into three groups by quantile: (1) group I corresponds to the dynamic domain boundaries; (2) group II corresponds to the moderately dynamic domain boundaries; and (3) group III corresponds to the relatively stable ones. We analyzed the CTCF binding within these different domain boundary groups. We used the peaks called from the CTCF ChIA-PET data in WTC-11 (see Methods). We found that both the number of CTCF binding peaks and the average peak intensity are positively correlated with the domain boundary occurrence frequencies, which is consistent with the observation in [10] based on multiplexed STORM imaging data (see Fig. 3g). Specifically, the group I boundaries have less overlap with the CTCF binding. On the other hand, group II and III boundaries are more enriched with CTCF binding. Notably, group II boundaries, which appear in less than 50% of the cell population, still have similar CTCF binding compared to more stable ones. Instead of analyzing only the reproducible peaks, we repeated the analysis on each replicate and observed the same patterns (Fig. S18).

A.14 Analysis of using single-cell A/B compartment scores as embeddings

Tan et al. [29] reported that after PCA the single cell A/B compartment scores (referred to as scA/B compartment scores) can be used as embeddings to identify cell types and subtypes on a Dip-C dataset with higher coverage. In the main text, we showed that by using only the chromatin conformation information of the sn-m3c-seq dataset as input, Higashi embeddings can already separate major neuron subtypes (Fig. 4a), providing major conceptual advance compared with previous work (note that [4] reported that chromatin conformation does not have enough information to identify complex neuron subtypes). In this section, we sought to test if scA/B compartment scores can be used as embeddings

on the sn-m3c-seq dataset as well. It is worth noting that the sn-m3c-seq dataset from [4] has lower coverage as compare to the Dip-C dataset from [29].

In [29], scA/B compartment scores are approximated by the CpG frequency. The scA/B compartment scores are further rank-normalized within each cell. Besides rank-normalization, we also tested another normalization method that is less sensitive and more robust, which is the quantile transform with the maximum number of quantiles chosen as 20% of the number of bins per cell. This would be equivalent to merging similar ranks into the same value in the rank-normalized scA/B compartment scores. The scA/B compartment scores calculated based on CpG frequency and normalized by these two approaches are referred to as "scA/B (cpg) - RankTie" and "scA/B (cpg) - Quantile", respectively. These scA/B compartment scores are further transformed through PCA with the top 20 PCs kept for UMAP visualization (the same as [29]). For comparisons, we also included the scA/B compartment scores calculated using the approach proposed in this work (see Supplementary Notes A.5 for details), which are further normalized with quantile transform (referred to as "scA/B (Higashi) - Quantile"). All scA/B compartment values are in 1Mb resolution.

As shown in Fig. S19, all embeddings based on the scA/B compartment scores cannot separate neuron subtypes as reliably as the Higashi embeddings (Fig. 4a), highlighting the advantages of Higashi for generating scHi-C embeddings. We also observed that the scA/B compartment scores calculated based on CpG frequency show apparent batch effects, whereas such batch effects are mitigated with scA/B (Higashi) (Fig. S19). This strongly suggests Higashi's ability in removing batch effects when imputing the contact maps. We also found a better separation of inhibitory and excitatory neuron subtypes and overall better separation among non-neurons with the UMAP visualization of scA/B (Higashi) (Fig. S19).

Taken together, we demonstrate the clear advantage of Higashi embeddings in separating complex cell types. Additionally, our method for calculating single-cell compartment scores based on the Higashi imputed contact maps is more robust to the batch effects and can reflect the cell types more accurately.

A.15 Cell type-specific 3D genome structures correlate with marker gene transcription

For the sn-m3c-seq dataset of human PFC, we assessed the correlation between the single-cell 3D genome structure and the single-cell transcriptome. We obtained the scRNA-seq of multiple cortical areas of the human brain from the Allen Brain map [31, 32]. We then calculated the marker genes for cell types or subtypes that are shared across the annotations in the scRNA-seq and sc-m3c-seq datasets, including astrocyte (Astro), oligodendrocyte (ODC), oligodendrocyte progenitor cell (OPC), endothelial cell (Endo), microglia (MG), neurons, excitatory neurons, inhibitory neurons, L2-3, L5-6, Vip, Pvalb, and Sst. The marker genes were identified using Seurat [25, 26] with the default parameters. The genes were then ranked by the log fold-change value between a specific cell type and the background and only the top 200 were kept for further analysis. We refer to the top 200 marker genes for a specific cell type as its marker gene set. For all the major cell types, the background is the remaining cells except the cell type of interest. When identifying the excitatory and inhibitory neuron marker genes, the background is the remaining neuron cells. When identifying the L2-3 and L5-6 marker genes, the background is the remaining excitatory neurons. Similarly, for Vip, Pvalb, and Sst, the background is the remaining inhibitory neurons.

We calculated the average scA/B values within the genomic regions covered by a marker gene set. Before averaging, the scA/B values were centered per bin across all cells. After averaging, the scA/B values were centered again per cell across marker gene sets. For bins covered by multiple genes within

a marker gene set, it would only be counted once. We observed that for each marker gene set, scA/B values of a cell type show a strong correlation with its marker gene's transcriptional activity. Specifically, each marker gene set has the highest average scA/B values within the corresponding cell type for all non-neuron cell types, excitatory neurons, and inhibitory neurons (Fig. S20a). Moreover, the scA/B values show a tendency in separating neuron subtypes L2-3, L5-6, Vip, Pvalb, and Sst (Fig. S21b), which is consistent with the observation based on the embeddings calculated from the scA/B values (Fig. S19b).

We then repeated the above analysis process by replacing the scA/B values with the single-cell insulation scores. We observed that the single-cell insulation scores correlate with the marker gene transcription in the neuron subtypes (Fig. S20b), showing a better capability to separate the neuron subtypes. However, while the single-cell insulation scores also show cell type-specific patterns for major non-neuron cell types, its correlation to the transcriptional activity of marker genes appears to be less consistent (Fig. S21a). For instance, the Astro-specific marker genes have overall lower insulation scores in astrocytes while the rest marker gene sets have overall higher insulation scores in the corresponding cell types.

Taken together, we demonstrate the global correlation between the cell type-specific transcriptional activity and the 3D chromatin structures at single-cell level by analyzing the average scA/B values and single-cell insulation scores of the marker genes. The results show strong potential of using the scA/B values and the single-cell insulation scores calculated based on the Higashi imputed contact maps to annotate the clusters of scHi-C data from complex tissue.

References

[1] Liu, J., Lin, D., Yardımcı, G. G. & Noble, W. S. Unsupervised embedding of single-cell Hi-C data. *Bioinformatics* **34**, i96–i104 (2018).

[2] Zhou, J. *et al.* Robust single-cell Hi-C clustering by convolution-and random-walk–based imputation. *Proceedings of the National Academy of Sciences* 201901423 (2019).

[3] Kim, H.-J. *et al.* Capturing cell type-specific chromatin compartment patterns by applying topic modeling to single-cell Hi-C data. *PLoS Computational Biology* **16**, e1008173 (2020).

[4] Lee, D.-S. *et al.* Simultaneous profiling of 3D genome structure and DNA methylation in single human cells. *Nature Methods* 1–8 (2019).

[5] Hie, B., Cho, H., DeMeo, B., Bryson, B. & Berger, B. Geometric sketching compactly summarizes the single-cell transcriptomic landscape. *Cell Systems* **8**, 483–493 (2019).

[6] Vaswani, A. *et al.* Attention is all you need. *Advances in Neural Information Processing Systems* **30**, 5998–6008 (2017).

[7] Zhang, R., Zou, Y. & Ma, J. Hyper-SAGNN: a self-attention based graph neural network for hypergraphs. In *International Conference on Learning Representations (ICLR)* (2020).

[8] Hamilton, W., Ying, Z. & Leskovec, J. Inductive representation learning on large graphs. In *Advances in Neural Information Processing Systems*, 1024–1034 (2017).

[9] Kipf, T. N. & Welling, M. Semi-supervised classification with graph convolutional networks. *arXiv preprint arXiv:1609.02907* (2016).

[10] Bintu, B. *et al.* Super-resolution chromatin tracing reveals domains and cooperative interactions in single cells. *Science* **362** (2018).

[11] Yang, T. *et al.* HiCRep: assessing the reproducibility of Hi-C data using a stratum-adjusted correlation coefficient. *Genome Research* **27**, 1939–1949 (2017).

[12] Su, J.-H., Zheng, P., Kinrot, S. S., Bintu, B. & Zhuang, X. Genome-scale imaging of the 3D organization and transcriptional activity of chromatin. *Cell* (2020).

[13] Lieberman-Aiden, E. *et al.* Comprehensive mapping of long-range interactions reveals folding principles of the human genome. *Science* **326**, 289–293 (2009).

[14] Leemans, C. *et al.* Promoter-intrinsic and local chromatin features determine gene repression in LADs. *Cell* **177**, 852–864 (2019).

[15] Chen, Y. *et al.* Mapping 3D genome organization relative to nuclear compartments using TSA-seq as a cytological ruler. *Journal of Cell Biology* **217**, 4025–4048 (2018).

[16] Zhang, L. *et al.* Tsa-seq reveals a largely "hardwired" genome organization relative to nuclear speckles with small position changes tightly correlated with gene expression changes. *bioRxiv* 824433 (2020).

[17] Wang, Y. *et al.* SPIN reveals genome-wide landscape of nuclear compartmentalization. *Genome Biology* **22**, 1–23 (2021).

[18] Hafemeister, C. & Satija, R. Normalization and variance stabilization of single-cell rna-seq data using regularized negative binomial regression. *Genome Biology* **20**, 1–15 (2019).

[19] Geiler-Samerotte, K. *et al.* The details in the distributions: why and how to study phenotypic variability. *Current Opinion in Biotechnology* **24**, 752–759 (2013).

[20] Van Dijk, D. *et al.* Recovering gene interactions from single-cell data using data diffusion. *Cell* **174**, 716–729 (2018).

[21] Crane, E. *et al.* Condensin-driven remodelling of X chromosome topology during dosage compensation. *Nature* **523**, 240–244 (2015).

[22] Cresswell, K. G. & Dozmorov, M. G. TADCompare: An R package for differential and temporal analysis of topologically associated domains. *Frontiers in Genetics* **11**, 158 (2020).

[23] Sauerwald, N. & Kingsford, C. Quantifying the similarity of topological domains across normal and cancer human cell types. *Bioinformatics* **34**, i475–i483 (2018).

[24] Friedman, C. E. *et al.* Single-cell transcriptomic analysis of cardiac differentiation from human PSCs reveals HOPX-dependent cardiomyocyte maturation. *Cell Stem Cell* **23**, 586–598 (2018).

[25] Butler, A., Hoffman, P., Smibert, P., Papalexi, E. & Satija, R. Integrating single-cell transcriptomic data across different conditions, technologies, and species. *Nature Biotechnology* **36**, 411–420 (2018).

[26] Stuart, T. *et al.* Comprehensive integration of single-cell data. *Cell* **177**, 1888–1902 (2019).

[27] Stevens, T. J. *et al.* 3D structures of individual mammalian genomes studied by single-cell Hi-C. *Nature* **544**, 59–64 (2017).

[28] Tan, L., Xing, D., Chang, C.-H., Li, H. & Xie, X. S. Three-dimensional genome structures of single diploid human cells. *Science* **361**, 924–928 (2018).

[29] Tan, L. *et al.* Changes in genome architecture and transcriptional dynamics progress independently of sensory experience during post-natal brain development. *Cell* (2021).

[30] Dixon, J. R. *et al.* Topological domains in mammalian genomes identified by analysis of chromatin interactions. *Nature* **485**, 376 (2012).

[31] Hawrylycz, M. J. *et al.* An anatomically comprehensive atlas of the adult human brain transcriptome. *Nature* **489**, 391–399 (2012).

[32] Hodge, R. D. *et al.* Conserved cell types with divergent features in human versus mouse cortex. *Nature* **573**, 61–68 (2019).

[33] Ramani, V. *et al.* Massively multiplex single-cell Hi-C. *Nature Methods* **14**, 263 (2017).

[34] Nagano, T. *et al.* Cell-cycle dynamics of chromosomal organization at single-cell resolution. *Nature* **547**, 61 (2017).

B Supplementary Tables

Datasets	Cell type / Cell State	Count
Ramani et al. [33]	GM12878	33
	HAP1	233
	HeLa	267
	K562	87
	**Total**	620
4DN sci-Hi-C [3]	GM12878	2048
	H1ESC	2395
	HAP1	1338
	HFFc6	578
	IMR90	29
	**Total**	6388
Nagano et al. [34]	G1	280
	early-S	303
	late-S/G2	326
	mid-S	262
	**Total**	1171
WTC-11	**Total**	188
sn-m3c-seq. [4]	ODC	1245
	OPC	203
	Astro	449
	Endo	205
	MG	422
	NN1	100
	L2/3	551
	L4	131
	L5	180
	L6	86
	Pvalb	134
	Sst	217
	Ndnf	144
	Vip	171
	**Total**	4238

Table S1: The number of cells in all scHi-C datasets used in this work.

Datasets	Contacts per cell				Non-zero non-diagonal per cell				Total Possible
	Mean	Min	Median	Max	Mean	Min	Median	Max	
Ramani et al.	10.5k	2.1k	7.8k	109.6k	4.3k	1.1k	3.7k	25.5k	237.1k
4DN sci-Hi-C	5.6k	1.9k	3.8k	752.7k	4.1k	1.8k	3.2k	107.1k	223.4k
Nagano et al.	56.9k	3.1k	56.8k	267.1k	14.7k	2.5k	14.1k	46.5k	184.5k
sn-m3c-seq	136.6k	13.5k	151.3k	412.9k	28.0k	6.0k	2.9k	64.1k	225.0k
WTC-11	145.1k	22.5k	144.8k	278.3k	30.6k	11.6k	30.5k	51.5k	237.1k

Table S2: Statistics of the contacts/non-zero non-diagonal entries per cell at 1Mb resolution for the scHi-C datasets used in this work. The Nagano et al. data were mapped to the mm9 assembly. The Ramani et al. and sn-m3c-seq data were mapped to the hg19 assembly. Others were mapped to the hg38 assembly. Same as [4], when studying the sn-m3c-seq, only autosomal chromosomes were used.

Datasets	Contacts per cell				Total Possible
	Mean	Min	Median	Max	
Nagano et al.	4.4k	0.2k	4.4k	19.4k	16.5k
WTC-11	14.5k	2.2k	14.7k	25.3k	29.4k
Ramani et al.	0.9k	0.0k	0.7k	9.5k	29.6k
4DN sci-Hi-C	0.6k	0.0k	0.4k	77.3k	29.4k
sn-m3c-seq	14.0k	0.4k	15.5k	37.9k	29.6k

Table S3: Statistics of the contacts per cell at 1Mb resolution of chr2 for different datrasets. The Nagano et al. data were mapped to the mm9 assembly. The Ramani et al. and sn-m3c-seq data were mapped to the hg19 assembly. Others were mapped to the hg38 assembly.

Datasets	Contacts per cell				Non-zero non-diagonal per cell				Total Possible
	Mean	Min	Median	Max	Mean	Min	Median	Max	
Original WTC-11	12.1k	1.2k	12.1k	22.9k	2.9k	0.9k	2.9k	4.4k	30.9k
WTC-11 (1/3)	4.0k	0.4k	4.0k	7.7k	1.6k	0.3k	1.6k	2.8k	30.9k
WTC-11 (1/5)	2.4k	0.2k	2.4k	4.6k	1.2k	0.2k	1.2k	2.1k	30.9k
WTC-11 (1/10)	1.2k	0.1k	1.2k	2.4k	0.8k	0.1k	0.8k	1.4k	30.9k
WTC-11 (1/15)	0.8k	0.1k	0.8k	1.6k	0.6k	0.1k	0.6k	1.1k	30.9k
Nagano et al.	4.5k	0.2k	4.6k	20.4k	1.2k	0.2k	1.2k	3.8k	19.5k
Ramani et al.	1.0k	0.1k	0.7k	10.8k	0.4k	0.1k	0.3k	2.7k	31.1k
4DN sci-Hi-C	0.5k	0.1k	0.3k	66.7k	0.4k	0.1k	0.3k	11.6k	30.9k
sn-m3c-seq	11.9k	0.4k	13.1k	42.7k	2.7k	0.3k	2.9k	6.9k	31.1k
Dip-C GM12878	43.9k	32.2k	43.4k	67.5k	3.9k	3.2k	3.8k	5.0k	31.1k

Table S4: Statistics of the contacts/non-zero non-diagonal entries per cell at 1Mb resolution of chr1. The Nagano et al. data were mapped to the mm9 assembly. The Ramani et al., sn-m3c-seq, and Dip-C GM12878 data were mapped to the hg19 assembly. Others were mapped to the hg38 assembly.

Operation	Average runtime
Training without cd-GNN	61.3s / epoch
Training with cd-GNN ($k = 0$)	95.2s / epoch
Training with cd-GNN ($k = 4$, fast mode)	109.6s / epoch
Training with cd-GNN ($k = 4$, memory saving mode)	221.4s / epoch
Imputation (1Mb resolution, hg38, autosomal chromosomes)	0.2s / cell
Imputation (500Kb resolution, hg38, autosomal chromosomes)	0.8s / cell
Imputation (100Kb resolution, hg38, autosomal chromosomes)	21.3s / cell
Imputation (50Kb resolution, hg38, autosomal chromosomes)	76.8s / cell

Table S5: Runtime of key operations in Higashi

C Supplementary Figures

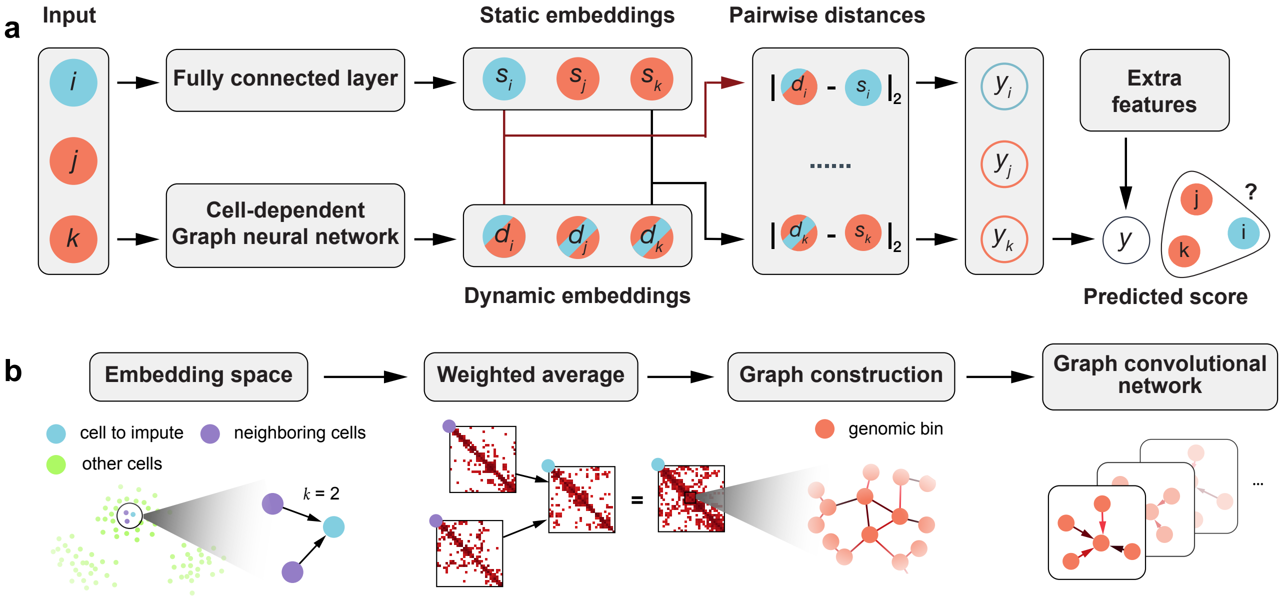

Figure S1: The structure of the hypergraph neural network used in Higashi. **a.** The input triplet consisting of one cell node and two bin nodes passes through two branches of the network to generate static embeddings and dynamic embeddings for each node, respectively. Then the pairwise distances between static and dynamic embedding pairs are calculated. These pairwise distances are combined with extra features such as genomic distance between the two bins to produce the final predicted score for the input triplet, which represents the probability of an entry in the single-cell contact map. **b.** The schematic of the cell-dependent graph neural network in Higashi. During training and imputation, k nearest neighboring cells in the embedding space are selected. The contact maps of the neighboring cells and the cell to impute are combined to construct a cell-dependent graph where the nodes are genomic bins. This graph as well as the attributes of the bin nodes are used as the input of a graph convolutional network.

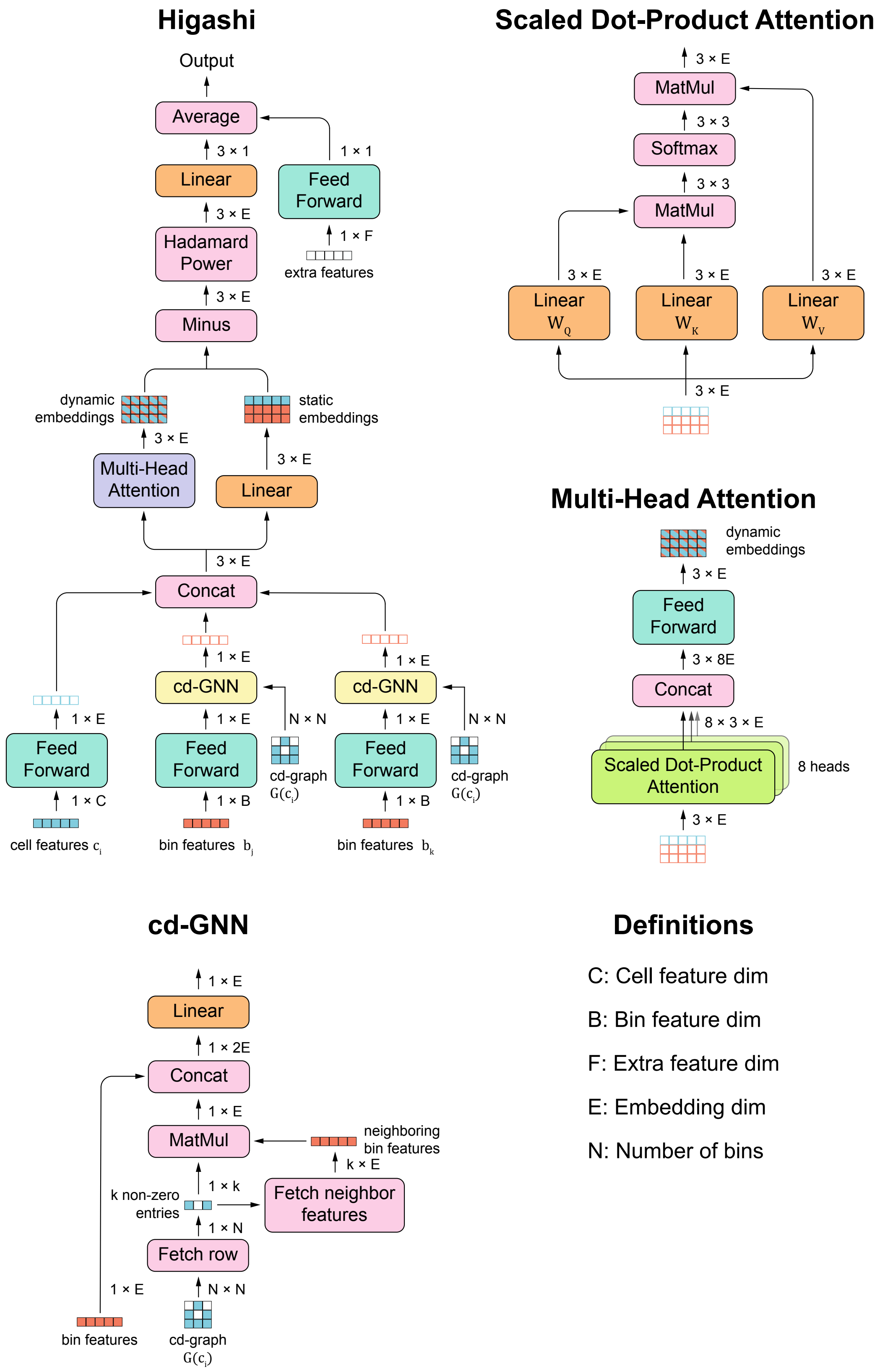

Figure S2: Overview of the detailed neural network architecture used in Higashi. Neural network structure abbreviations in the figure: cd-GNN: cell dependent graph neural network; Concat: concatenation; Linear: linear layer, also referred to as the fully-connected layer; Fetch row: slice the corresponding row in the adjacency matrix; Fetch neighbor features: collect the bin features of the neighboring nodes in the graph; MatMul: matrix multiplication.

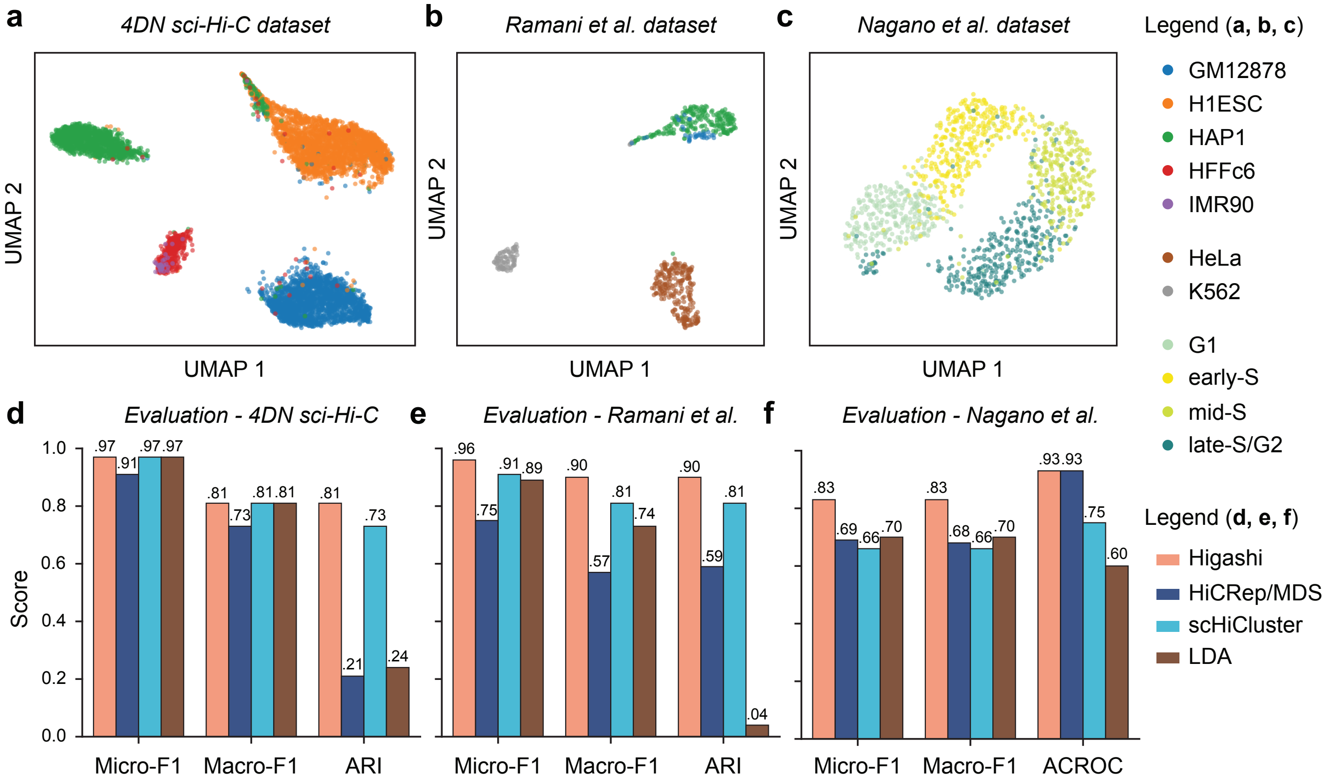

Figure S3: Evaluation of the embeddings generated by Higashi. **a, b, c.** The UMAP visualization of the embeddings produced by Higashi for three scHi-C datasets (4DN sci-Hi-C [3], Ramani et al. [33], and Nagano et al. [34] datasets). **d, e, f.** Quantitative evaluation of Higashi on the three scHi-C datasets by comparing to HiCRep/MDS [1], scHiCluster [2], and LDA [3]. The performances are measured by Micro-F1, Macro-F1, Adjusted Rand Index (ARI), and also ACROC scores from the supervised and unsupervised cell type identification tasks.

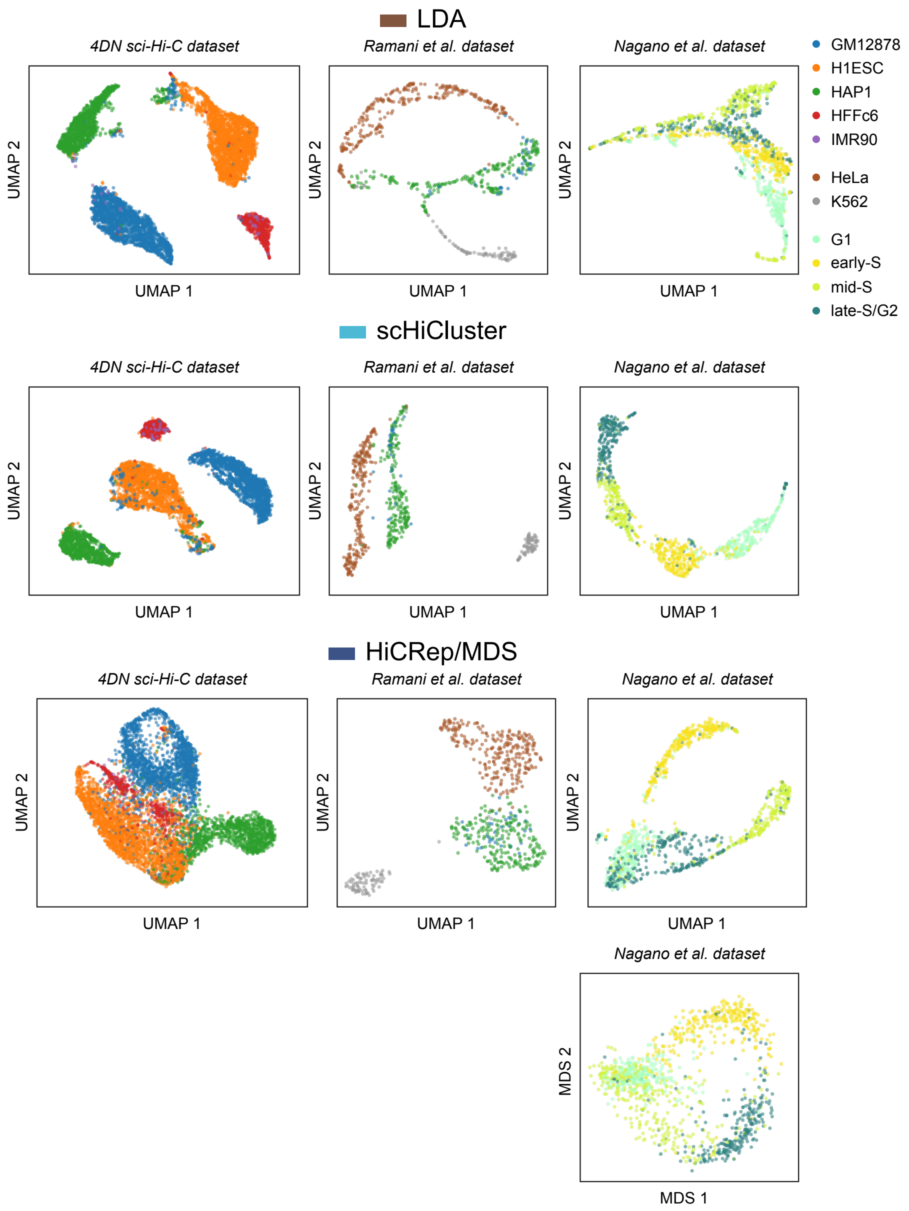

Figure S4: Visualization of the embeddings for three scHi-C datasets (4DN sci-Hi-C [3], Ramani et al. [33], and Nagano et al. [34] datasets) produced by three scHi-C embedding methods: HiCRep/MDS [1], scHiCluster [2], and LDA [3]. All visualizations are produced by UMAP except for HiCRep/MDS on the Nagano et al. dataset. In the original paper, the nascent MDS was preferred for visualizing cell cycle data by choosing the dimension of MDS as 2.

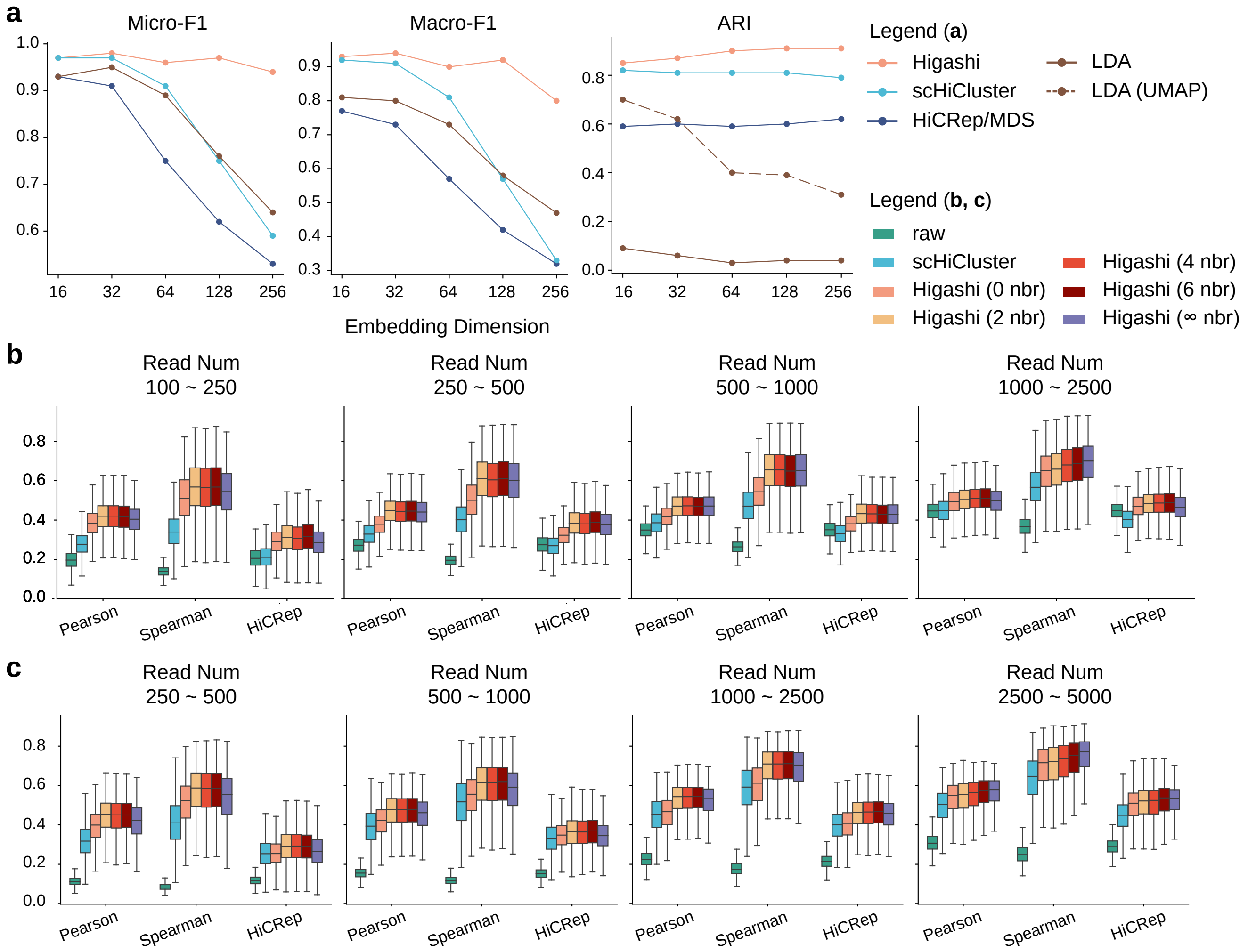

Figure S5: Higashi are robust to hyperparameter selections. **a.** The performance of the supervised and unsupervised cell type identification tasks measured by Micro-F1, Macro-F1, and Adjusted Rand Index (ARI) scores over different scHi-C analysis methods (Higashi, HiCRep/MDS [1], scHiCluster [2] and LDA [3]) and different embedding dimensions. The experiment was done on the Ramani et al. dataset. **b, c.** The performance of the contact map imputation measured by the Pearson correlation, Spearman correlation, and HiCRep [11] scores over the baseline methods and Higashi with different numbers of neighboring cells in the embedding space for imputation. The evaluation in **(b)** was done using the simulation data (n=11,631 contact maps) based on the imaging data from [10]. The experiment in **(c)** was done using the simulation data (n=3,029 contact maps) based on the imaging data from [12]. For the boxplots, the middle line is the median, the lower and upper lines correspond to the first and third quartile, and the upper and lower whiskers extend to values no further than 1.5 × IQR (inter-quartile range).

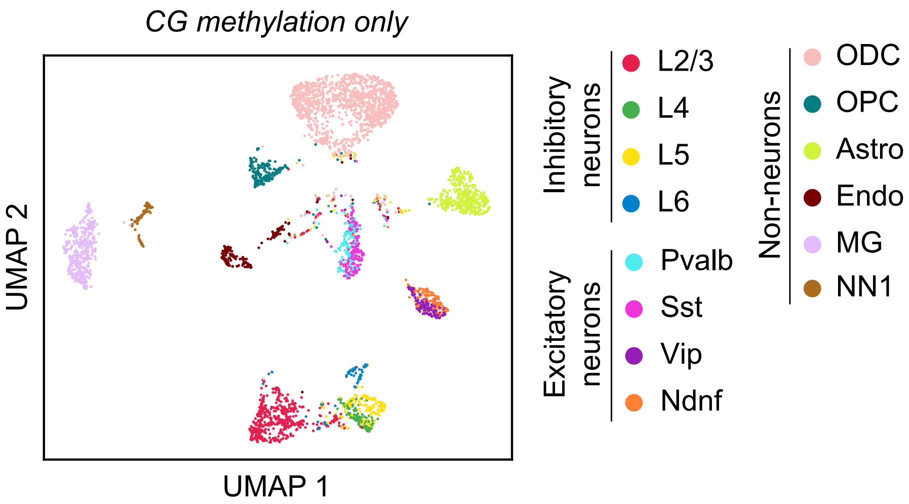

Figure S6: UMAP visualization of the embeddings using CG methylation only. The embeddings are generated by Scanorama [5]. The dimension size is chosen as 64.

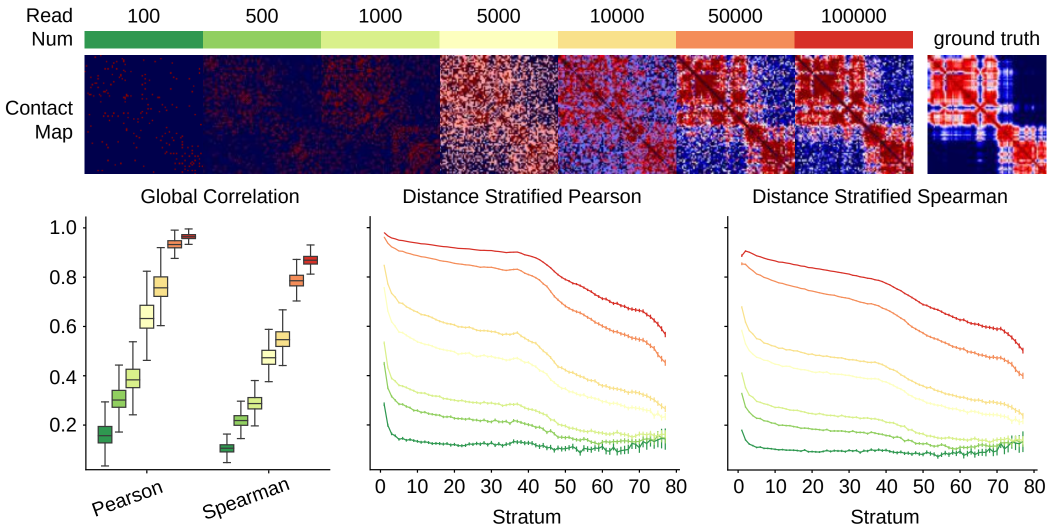

Figure S7: Similarities between the simulated contact frequency maps and the ground truth probability map over different read numbers. Simulation is based on the 3D chromosome imaging data from [10] (n=11,631 contact maps). The similarities are quantified by the global Pearson and Spearman correlation scores as well as the distance stratified Pearson and Spearman correlation scores. For the boxplots, the middle line is the median, the lower and upper lines correspond to the first and third quartile, and the upper and lower whiskers extend to values no further than 1.5 $\times$ IQR (inter-quartile range).

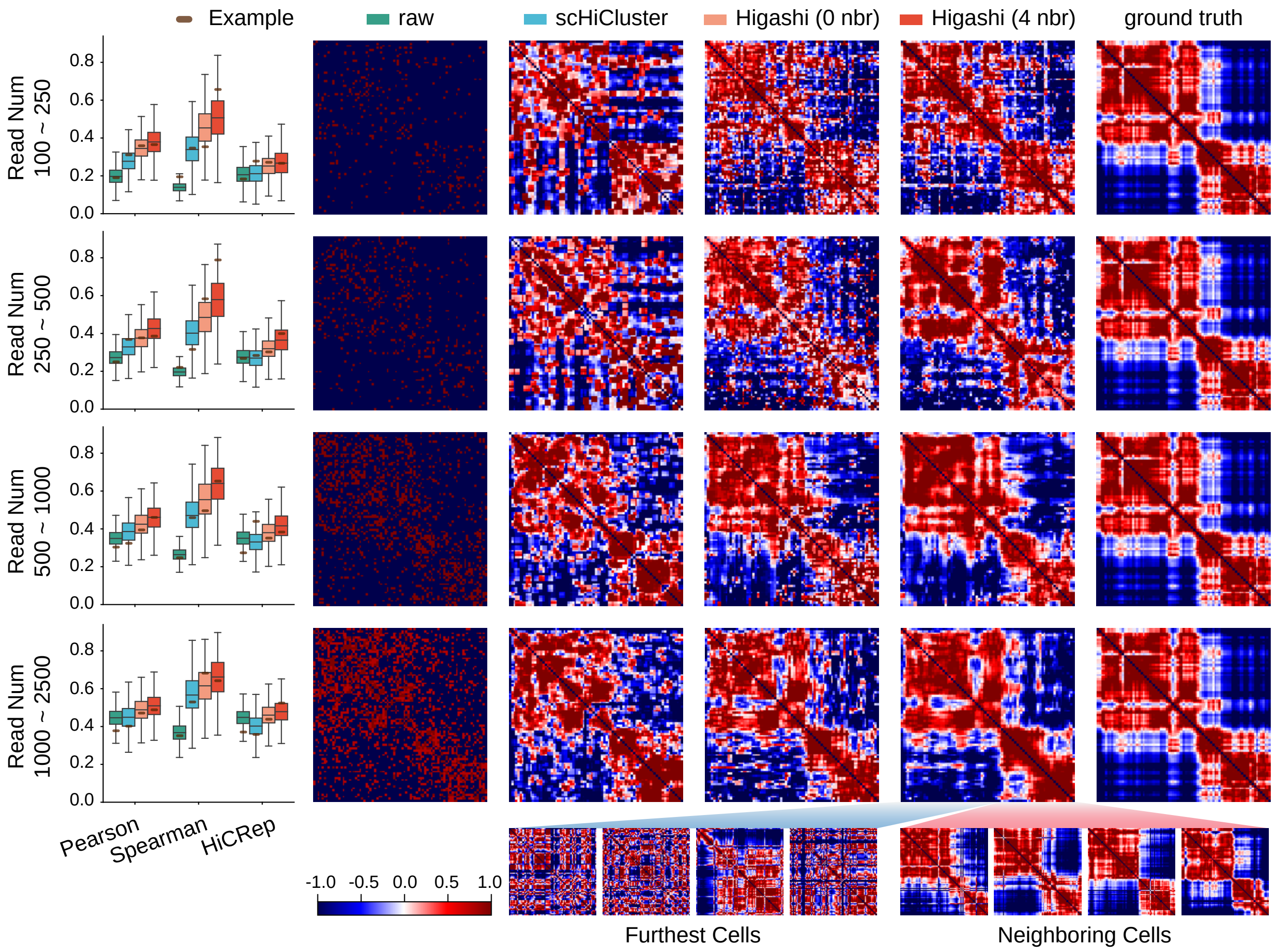

Figure S8: Evaluation and visualization of different imputation methods on the simulated scHi-C data based on the 3D chromosome imaging data from [10]. Each row corresponds to one set of simulation data with a chosen range of the read number. The boxplots (n=11,631 contact maps) illustrate the quantitative evaluation of the similarities between the raw, the scHiCluster enhanced, the Higashi enhanced contact maps, and the ground truth (inverse distance map). In the boxplots, the middle line is the median, the lower and upper lines correspond to the first and third quartile, and the upper and lower whiskers extend to values no further than 1.5 $\times$ IQR (inter-quartile range). The heatmaps visualize the contact map before and after imputation as well as the ground truth. The contact maps of the neighboring cells in the embedding space that help the imputation are included. As a reference, the contact maps of the cells that are furthest in the embedding space are also visualized. Related to Fig. 2e.

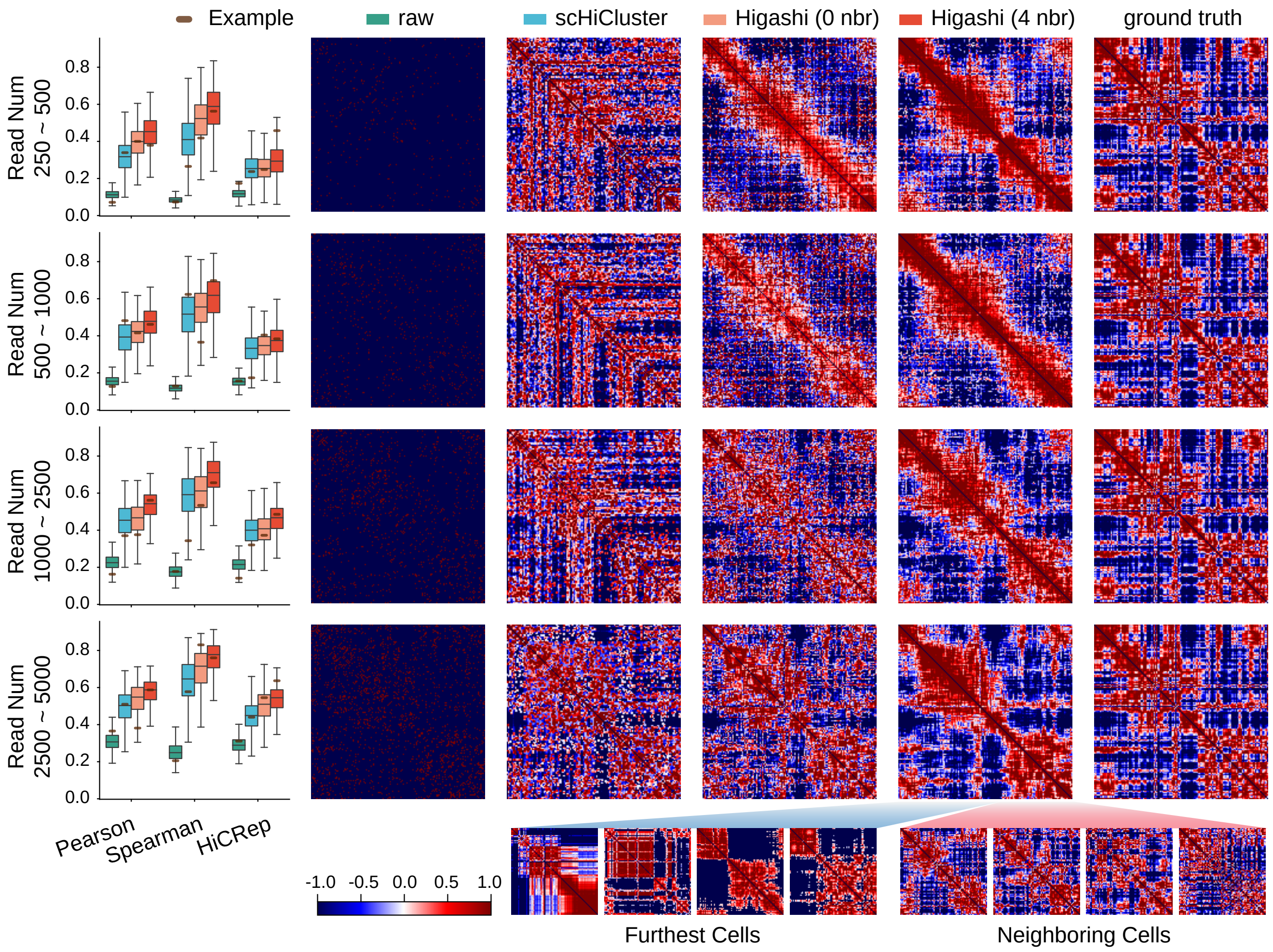

Figure S9: Evaluation and visualization of different imputation methods on the simulated scHi-C data based on the 3D genome imaging data from [12]. Each row corresponds to one set of simulation data with a chosen range of the read number. The boxplots (n=3,029 contact maps) illustrate the quantitative evaluation of the similarities between the raw, the scHiCluster enhanced, the Higashi enhanced contact maps, and the ground truth (inverse distance map). In the boxplots, the middle line is the median, the lower and upper lines correspond to the first and third quartile, and the upper and lower whiskers extend to values no further than 1.5 $\times$ IQR (inter-quartile range). The heatmaps visualize the contact map before and after imputation as well as the ground truth.

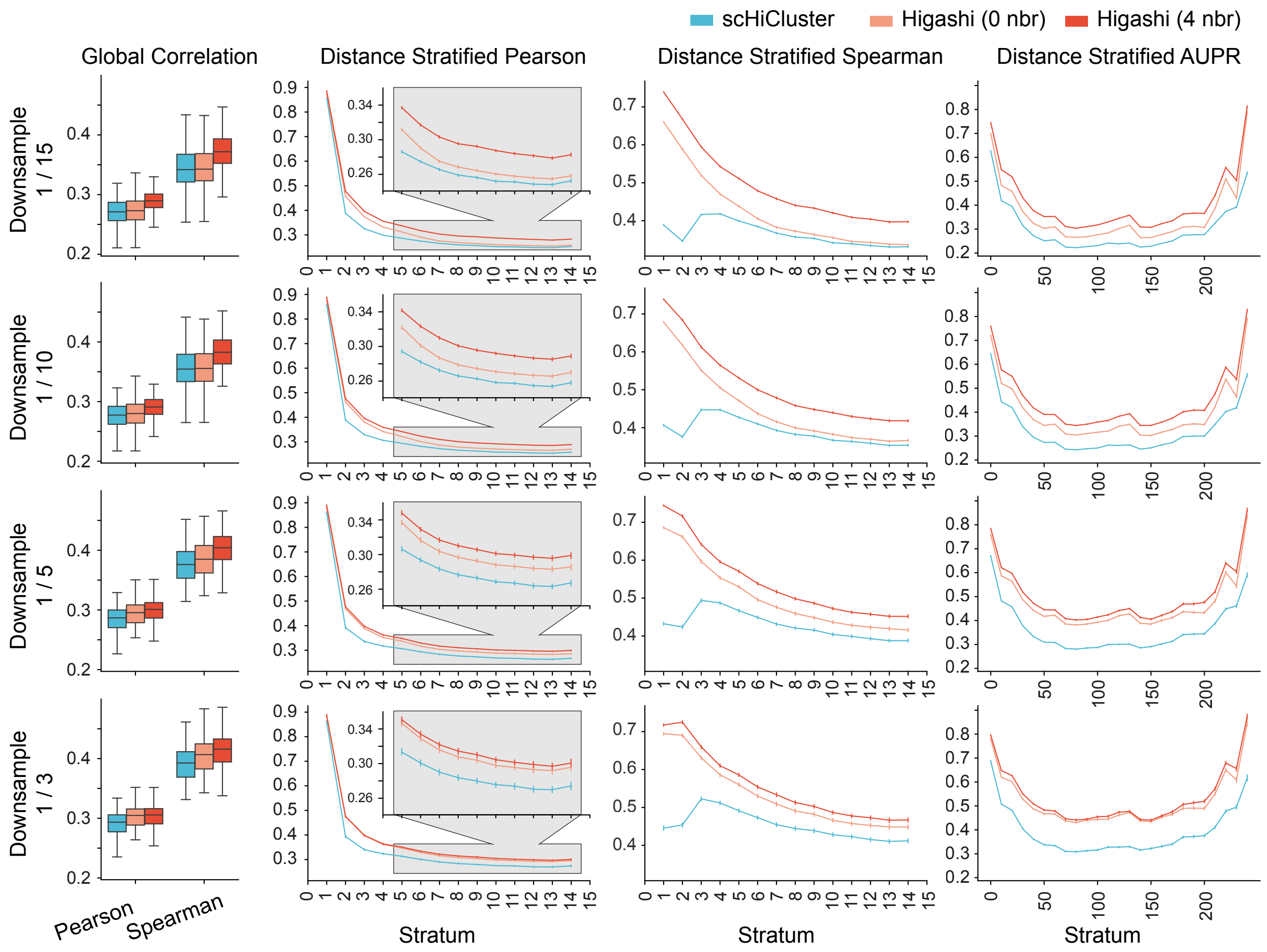

Figure S10: Evaluation of different imputation methods on the scHi-C data simulated by downsampling the WTC-11 scHi-C datasets. Each row corresponds to one set of downsampling rate. The boxplots (n=188 cells) illustrate the global correlation scores of contact maps before and after imputation versus the ground truth. In the boxplots, the middle line is the median, the lower and upper lines correspond to the first and third quartile, and the upper and lower whiskers extend to values no further than 1.5 $\times$ IQR (inter-quartile range). The line plots visualize the evaluation metrics versus each stratum. The evaluation metrics include the Pearson correlation and Spearman correlation for short-range interactions as well as AUPR (area under precision-recall) score for long-range interactions. The length of i-th stratum in the matrix is 249$-i$.

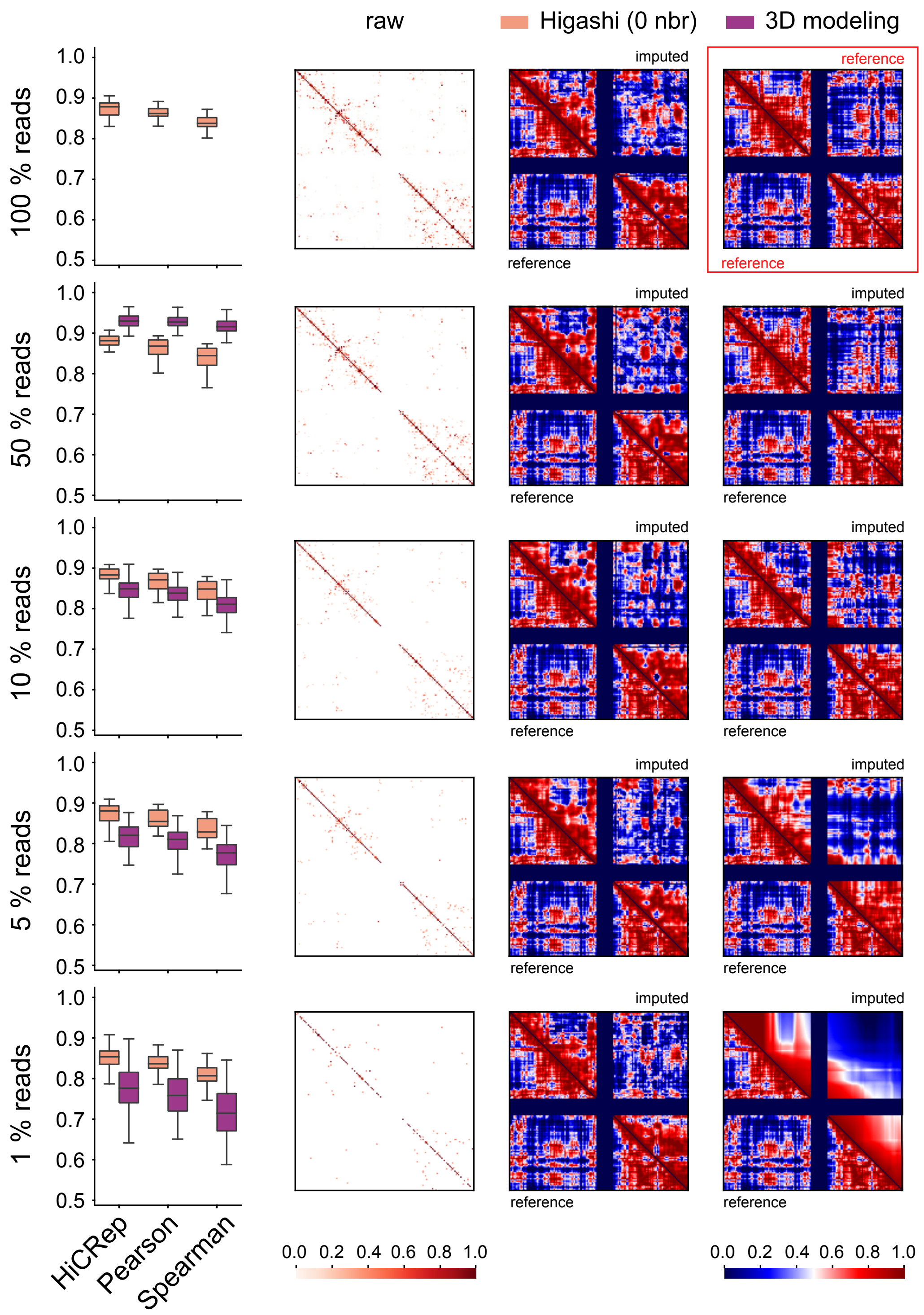

Figure S11: Comparisons between the Higashi imputations and the results from 3D structure modeling based on the GM12878 Dip-C dataset [28] (n=28 chromosomes). Each row corresponds to one set of downsampling percentage (1% to 100% of the original sequencing reads). The boxplot illustrates the similarity between the contact maps imputed by either Higashi or 3D structure modeling from the downsampled data and the contact maps imputed by 3D modeling from the full coverage dataset. In the boxplots, the middle line is the median, the lower and upper lines correspond to the first and third quartile, and the upper and lower whiskers extend to values no further than 1.5 × IQR (inter-quartile range). Examples of raw contact maps, imputed contact maps, and results from the 3D structure modeling on the full coverage datasets (denoted as "reference" in the lower left part of the contact matrices for Higashi and 3D modeling) are also shown.

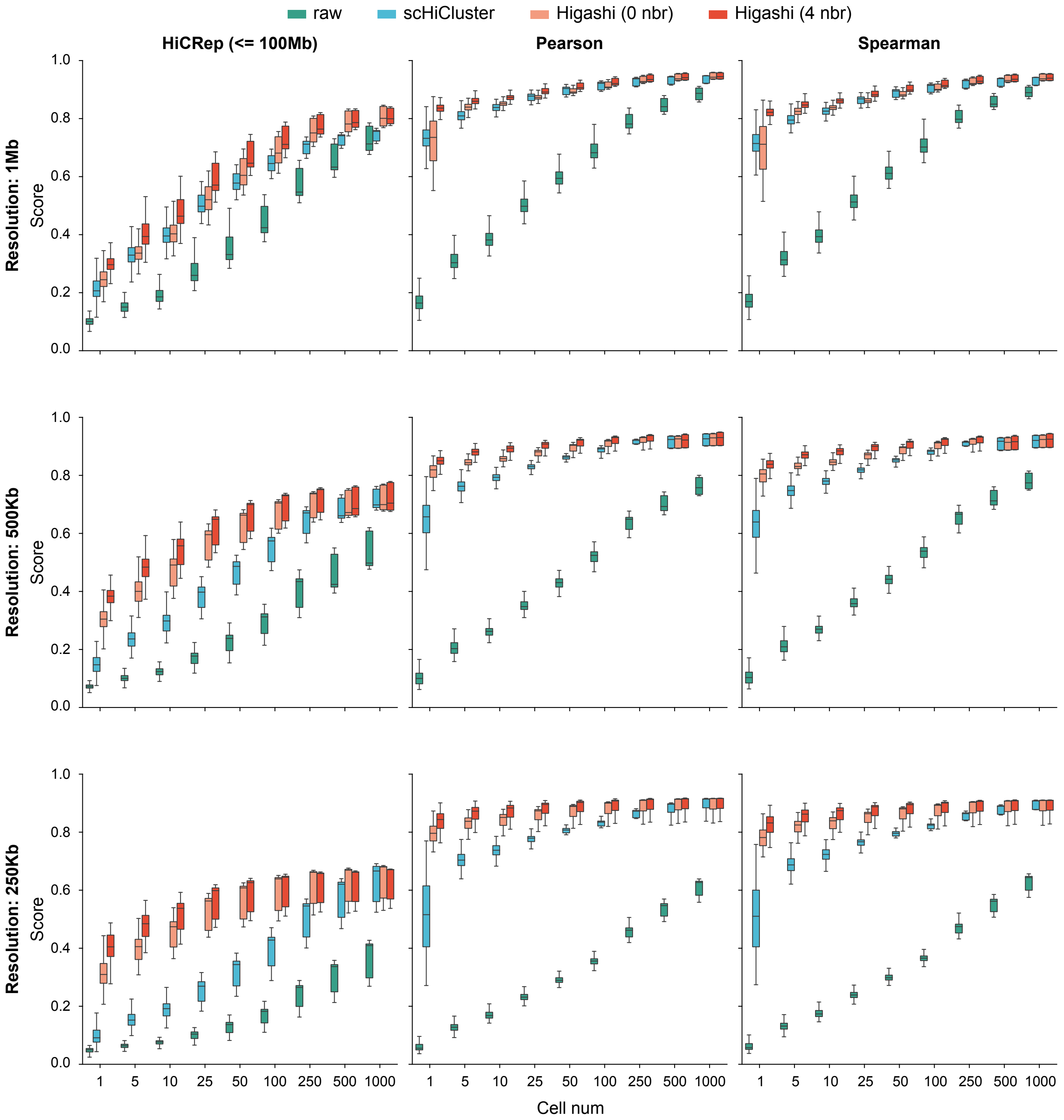

Figure S12: Comparisons between the pooled imputed scHi-C contact maps and the corresponding bulk Hi-C data. The results are based on GM12878, HAP1, and H1ESC cells in the 4DN sci-Hi-C dataset at 1Mb, 500Kb, and 250Kb resolutions. Each row corresponds to a specific resolution. The boxplot (n=30 independent experiments) illustrates the similarity between the pooled scHi-C contact maps imputed by different methods and the bulk Hi-C contact maps. In the boxplots, the middle line is the median, the lower and upper lines correspond to the first and third quartile, and the upper and lower whiskers extend to values no further than 1.5 $\times$ IQR (inter-quartile range). Results of three cell types share the same trend across different resolutions and cell numbers and are thus merged for visualizataion. Similarity scores are calculated for each chromosome individually and then weighted-averaged by the length of the chromosome (the same method used for calculating genome-wide HiCRep score [11]).

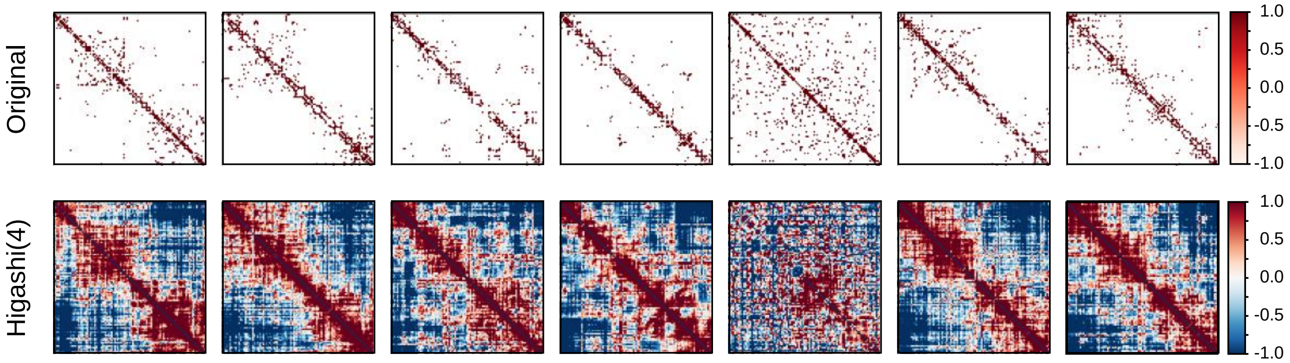

Figure S13: Examples of the Higashi imputation on WTC-11 scHi-C at 50Kb resolution (chr21:15Mb-20Mb)

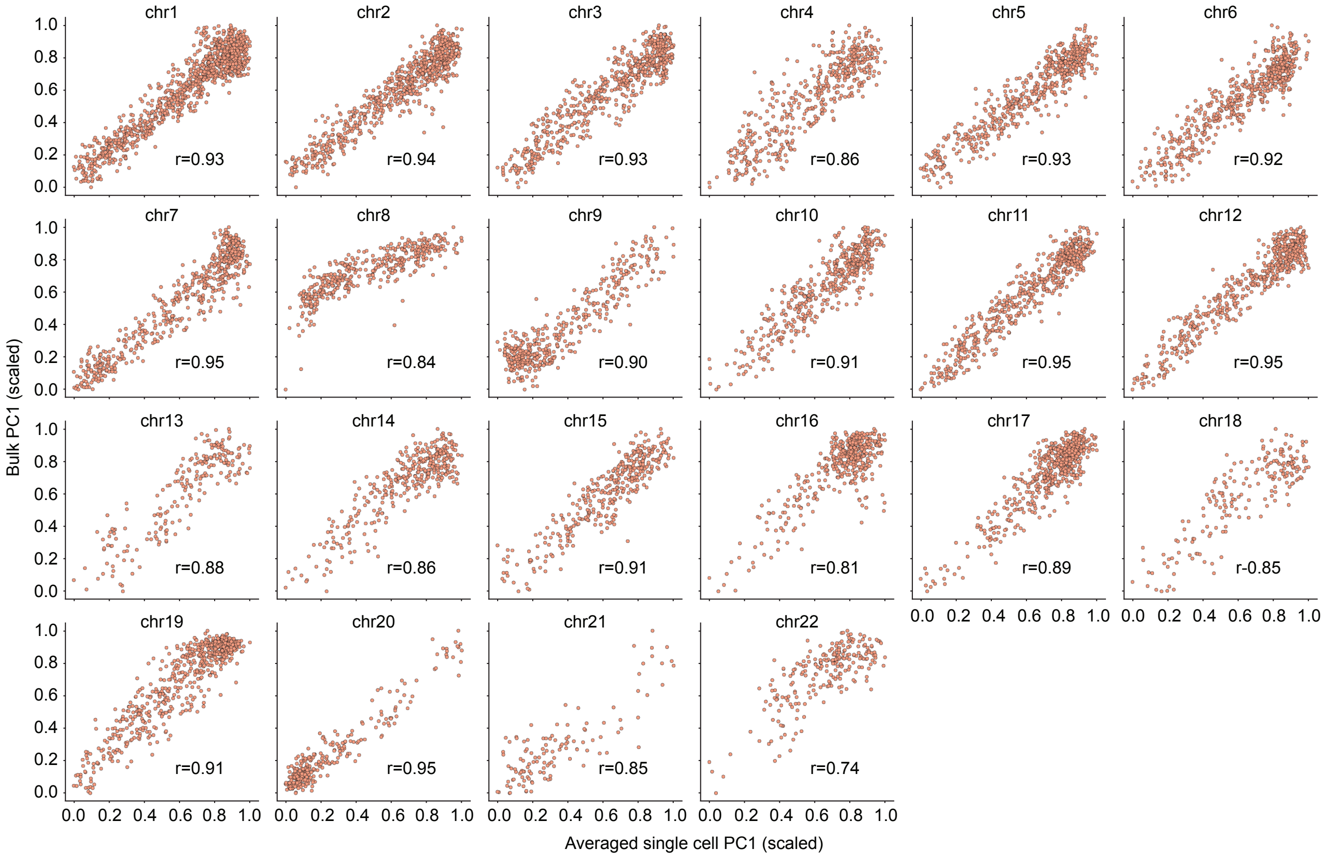

Figure S14: Scatter plot of the averaged single-cell A/B compartment score of WTC-11 scHi-C versus the A/B compartment score obtained from the bulk WTC-11 Hi-C. The Pearson correlation scores are also shown in the scatter plot.

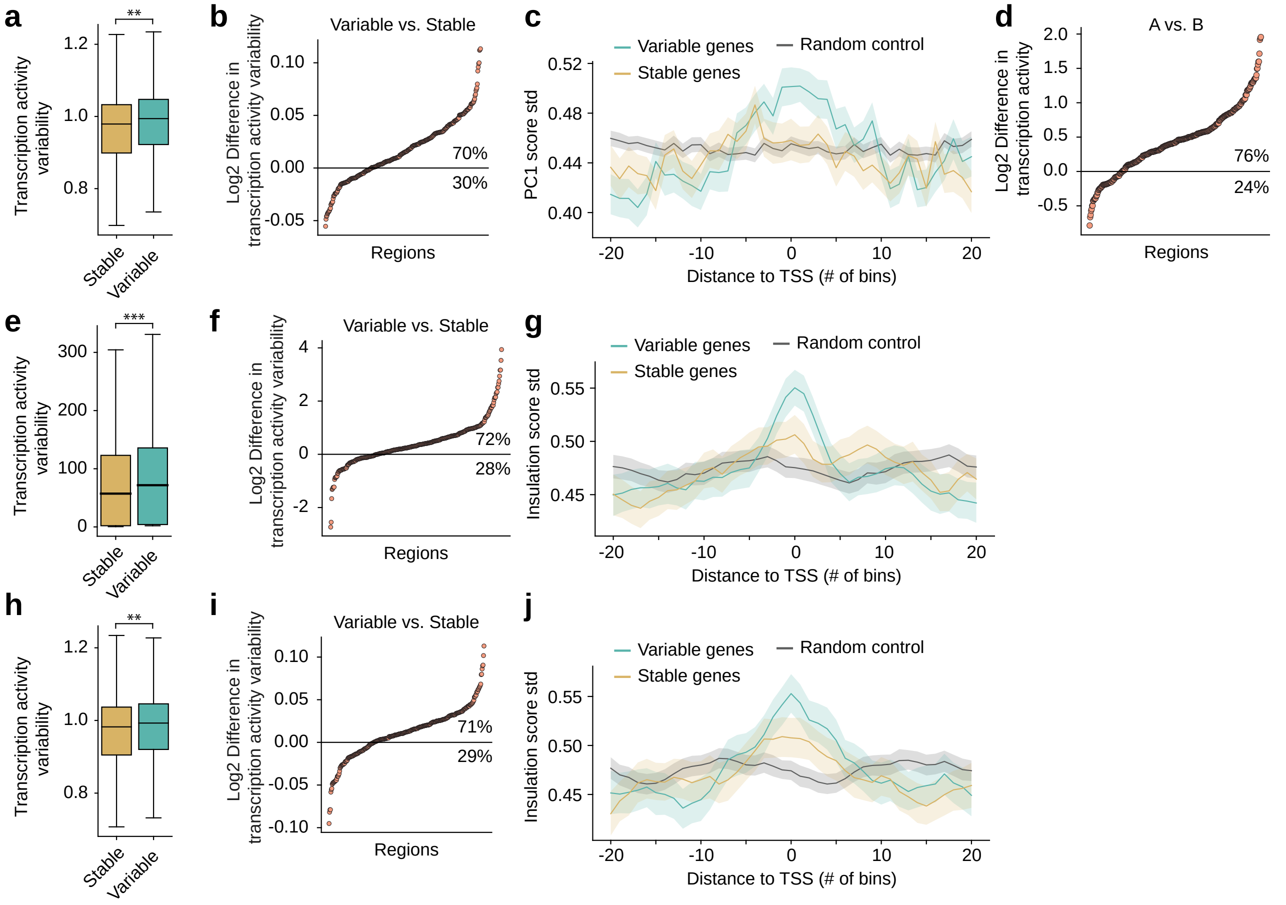

Figure S15: Correlation between the cell-to-cell variability of 3D genome features (A/B compartment scores and TAD-like domain boundaries) and transcriptional variability. **a.** Global comparisons of transcriptional variability on regions with variable and stable compartment annotations (n= 7,790 genes in total). There are 3,890 genes that have stable single-cell compartment scores with average transcription activity variability equal to 0.96. There are 3,900 genes that have dynamic single-cell compartment scores with average transcription activity variability equal to 0.97. The one sided t-test *P*-value = 9.0×10^{-3} **b.** Log2 difference of transcriptional variability of genes with variable versus stable compartment annotations within Mb scale windows. **c.** Visualization of standard deviation of the compartment scores around genes with variable or stable transcription level. The data are presented as mean values +/- 1.96 SEM (95% confidence interval). **d.** Log2 difference of the average transcription of genes in A compartments versus genes in B compartments within Mb scale windows. **e, h.** Global comparisons of transcriptional variability on regions with variable and stable insulation scores. In **(e)**, there are n=10,146 genes in total, including 5,071 genes that have stable single-cell compartment scores with average transcription activity variability equal to 77.1. There are 5,075 genes that have dynamic single-cell compartment scores with average transcription activity variability equal to 86.4. The one sided t-test *P*-value = 1.1×10^{-8}. In **(h)**, there are n=7,790 genes in total, including 3,890 genes that have stable single-cell compartment scores with average transcription activity variability equal to 0.96. There are 3,900 genes that have dynamic single-cell compartment scores with average transcription activity variability equal to 0.97. The one sided t-test *P*-value = 9.0×10^{-3}. **f, i.** Log2 difference of transcriptional variability of genes with variable versus stable insulation scores within a Mb scale window. **g, j.** Visualization of standard deviation of insulation scores around genes with variable or stable transcription level. The data are presented as mean values +/- 1.96 SEM (95% confidence interval). In panels **a,b,c,h,i,j**, the transcriptional variability is quantified as the residual variance calculated by a variance stabilization method developed for scRNA-seq data [18]. In panels **e,f,g**, the transcriptional variability is quantified as the coefficient of variation of the imputed scRNA-seq data [20]. In the boxplots of panels **a, e, h**, the middle line is the median, the lower and upper lines correspond to the first and third quartile, and the upper and lower whiskers extend to values no further than 1.5 $\times$ IQR (inter-quartile range). Related to Fig. 3b,c,d

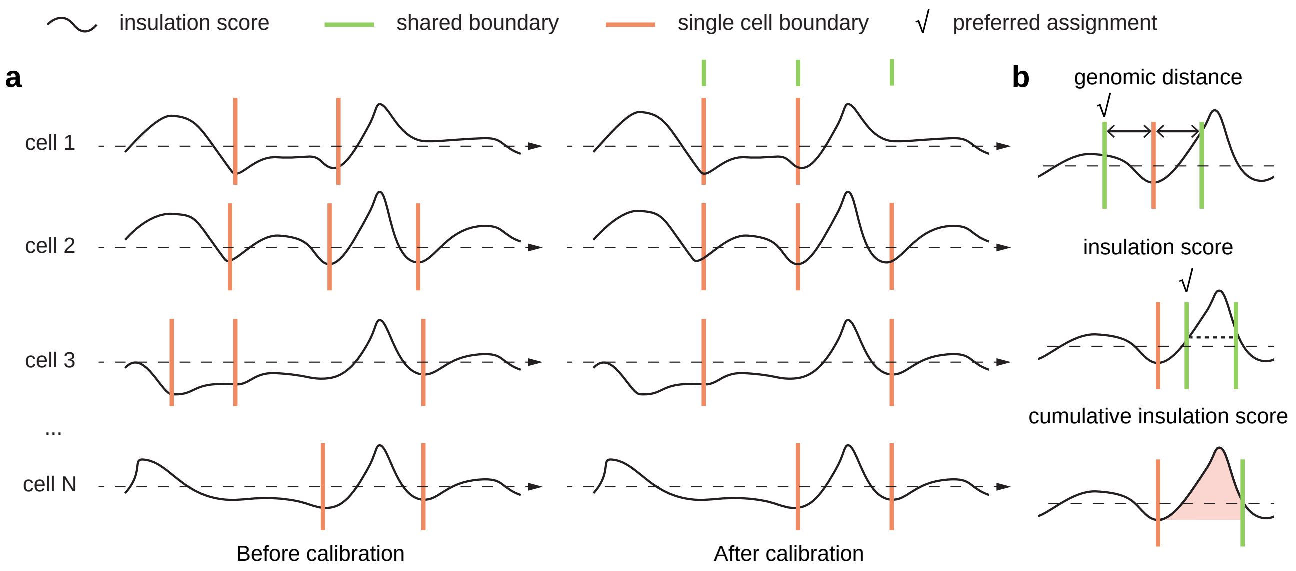

Figure S16: Illustration of TAD-like domain boundary calibration among single cells. **a.** Domain boundary calibration for boundaries called from scHi-C. **b.** Different measurements for comparing similarities of two domain boundaries with the given insulation score. Cases where the given metric cannot differentiate the preferred boundaries from the less appropriate ones are also visualized.

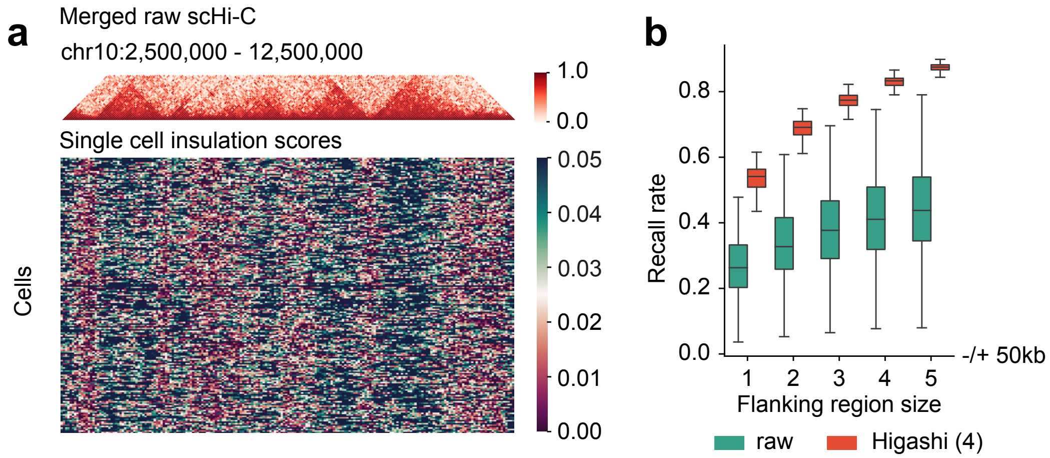

Figure S17: TAD-like domain boundary calling for the original WTC-11 scHi-C without imputation at 50Kb resolution. **a.** The merged scHi-C contact maps at chr10:2,500,000-12,500,000 and the calculated insulation scores are shown. Related to Fig. 3e. **b.** Evaluation for calling single-cell TAD-like boundaries using single-cell insulation scores calculated based on the raw or the Higashi (k=4) imputed contact maps. Specifically, we call bulk TAD boundaries using the insulation score based method on the pooled WTC-11 scHi-C data and record the bulk insulation score associated with each boundary. If the corresponding bin of a bulk TAD boundary has a lower single-cell insulation score in a specific cell, we consider that the TAD-like boundary is present in that specific cell. Due to different sparsity, the value-range of the insulation scores from the bulk contact map, the raw data, and the imputed single-cell insulation scores are very different. All these scores are z-score normalized before comparisons. We then check if the presence of this bulk TAD boundary in single cells can be validated by single-cell TAD-like boundaries called directly from the single-cell insulation score with flanking regions considered. The recall rate of the bulk TAD boundaries that are present in single cell is used as a metric for the accuracy of TAD calling. As shown by the boxplot (n=188 cells), Higashi significantly improves TAD-like boundary calling in individual cells after imputation. In the boxplot, the middle line is the median, the lower and upper lines correspond to the first and third quartile, and the upper and lower whiskers extend to values no further than 1.5 $\times$ IQR (inter-quartile range).

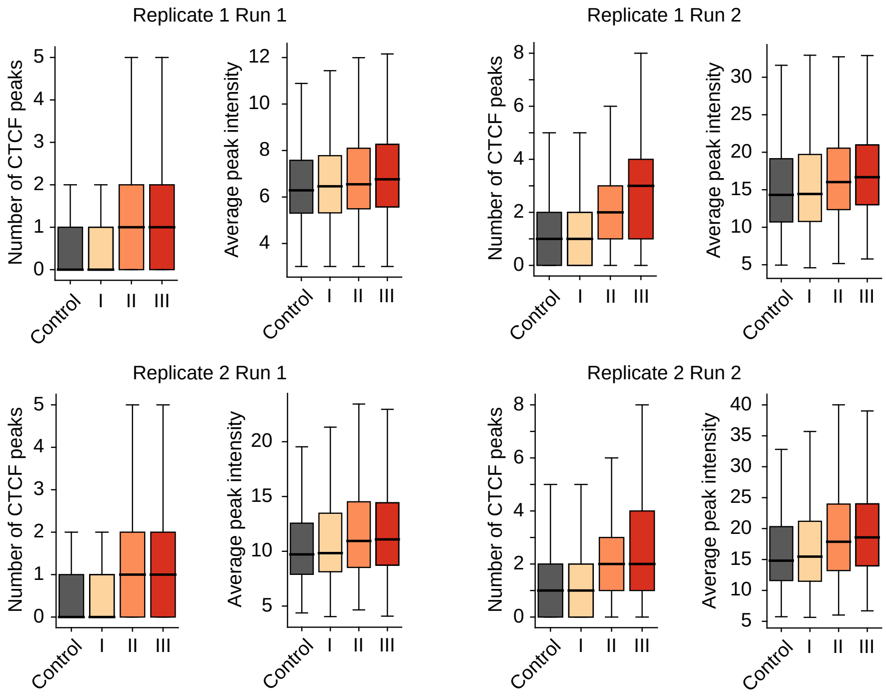

Figure S18: CTCF binding at domain boundaries from different occurrence frequency groups across the cell population. CTCF binding peaks were obtained on each batch from each replicate individually. Related to Fig. 3f,g. For the boxplots on the number of CTCF peaks at boundaries: n=8,004 boundaries in total, including 1,577 in control group, 2,137 in group I, 2,127 in group II, 2,163 in group III. For the boxplots on the average peak intensity, we have the following. Replicate1 Run1: n=4,320 boundaries with at least one CTCF binding, including 604 in control group, 873 in group I, 1,326 in group II, 1,517 in group III. Replicate1 Run2: n=6,032 boundaries with at least one CTCF binding, including 953 in control group, 1,378 in group I, 1,802 in group II, 1,899 in group III. Replicate2 Run1: n=4,616 boundaries with at least one CTCF binding, including 643 in control group, 932 in group I, 1,420 in group II, 1,621 in group III. Replicate2 Run2: n=5,959 boundaries with at least one CTCF binding, including 952 in control group, 1,354 in group I, 1,764 in group II, 1,889 in group III. In the boxplots, the middle line is the median, the lower and upper lines correspond to the first and third quartile, and the upper and lower whiskers extend to values no further than 1.5 $\times$ IQR (inter-quartile range).

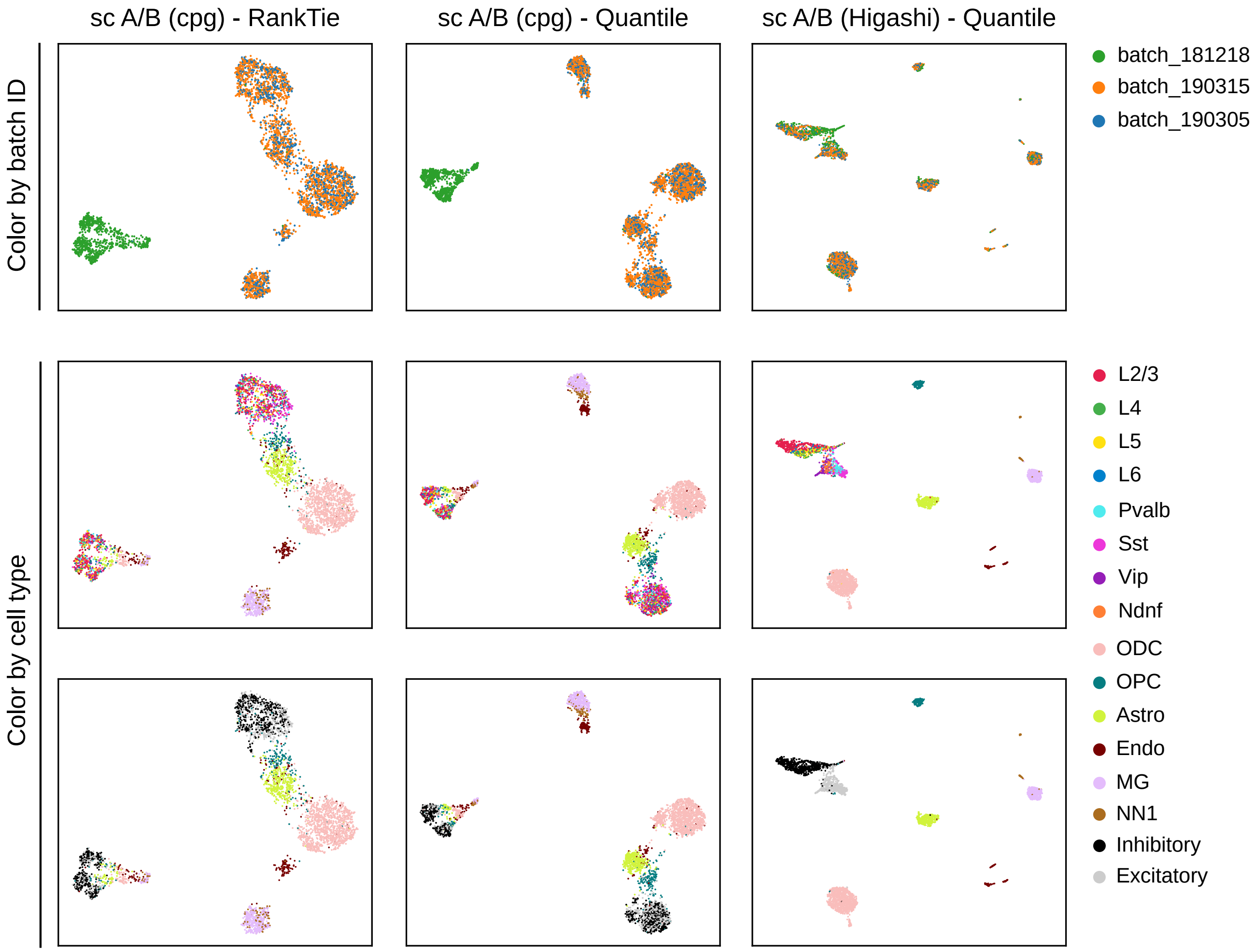

Figure S19: UMAP visualization of the single-cell A/B compartment scores based on the sn-m3c-seq dataset from human PFC. The rows are organized by different color schemes (i.e., batch ID or cell type). Each column corresponds to a specific combination of calculation method and normalization method for single-cell A/B compartment scores. The calculation method includes the approach using CpG density as the approximation (used in [29]) and the method in Higashi developed in this work. The A/B compartment values are further normalized with either RankTie (used in [29]) or quantile transformation.

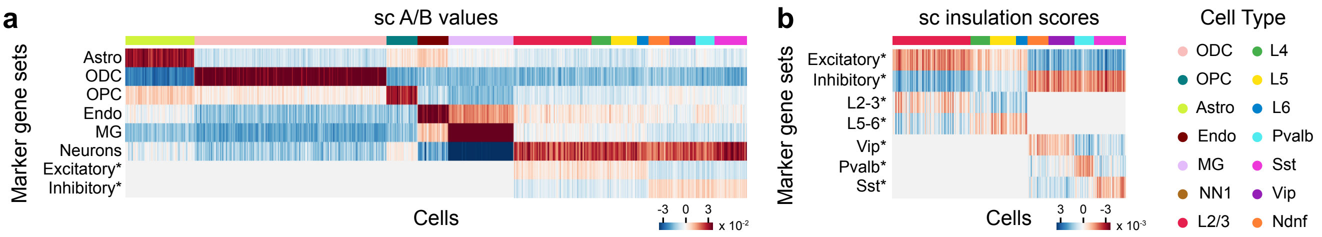

Figure S20: 3D genome features of cell type-specific marker genes among single cells. **a.** Average scA/B values (centered) of cell type-specific marker gene sets. **b.** Average single-cell insulation scores (centered) of cell type-specific marker gene sets. For each cell type, the top 200 marker genes were identified based on scRNA-seq. For marker genes without the "*" symbol, the background when identifying the marker genes is the rest of the cell population. For marker genes with the "*" symbol, the background is the remaining neurons, excitatory neurons, or inhibitory neurons, respectively.

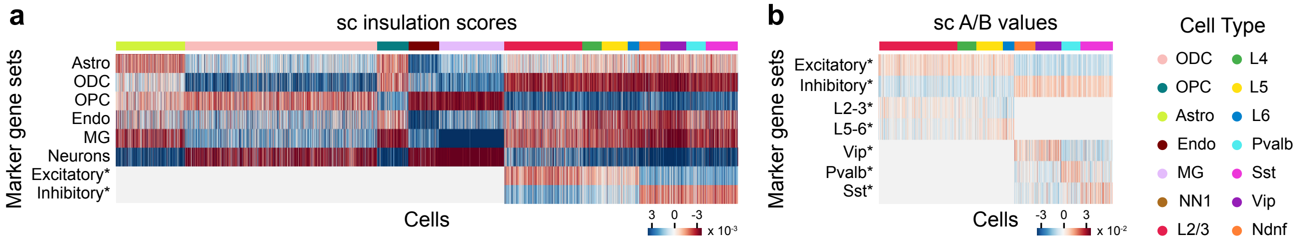

Figure S21: 3D genome features of cell type-specific marker genes among single cells. **a.** Average single-cell insulation scores (centered) of cell type-specific marker gene sets. **b.** Average scA/B values (centered) of cell type-specific marker gene sets. For each cell type, the top 200 marker genes were identified based on scRNA-seq. For marker genes without the "*" symbol, the background when identifying the marker genes is the rest of the cell population. For marker genes with the "*" symbol, the background is the remaining neurons, excitatory neurons, and inhibitory neurons, respectively. Related to Fig. S20

.

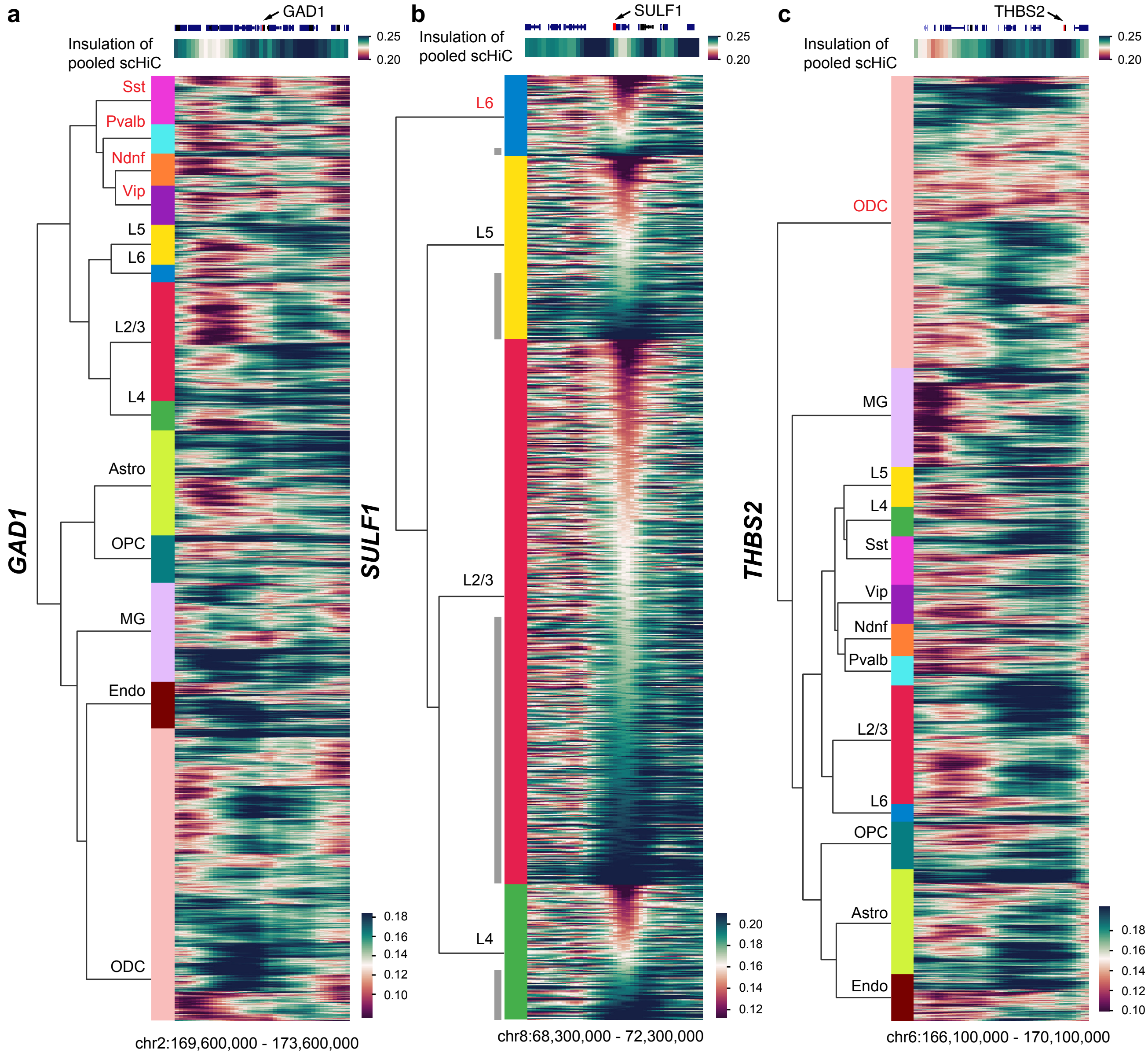

Figure S22: Single-cell insulation score heatmap of the flanking regions surrounding the genes in different cell types with distinct TAD-like boundary patterns specific to the highlighted cell type (in red font). **a.** Marker gene GAD1 for inhibitory neuron subtypes Sst, Pvalb, Ndnf, and Vip. Cells within each cell type are sorted with hierarchical clustering based on single-cell insulation scores. **b.** Marker gene SULF1 to distinguish L6 subtypes from the rest excitatory neuron subtypes (L2/3, L4, L5). Cells within each cell type are sorted according to the average insulation scores around the TAD-like domain boundary of interest. Cells without the presence of such boundary are indicated by the grey vertical bar. **c.** THBS2 for ODC. THBS2 has ODC-specific high expression. Cells within each cell type are sorted with hierarchical clustering based on single-cell insulation scores. Insulation scores that are calculated based on the pooled scHi-C contact maps of the whole population are also visualized as a reference at the top in each panel.

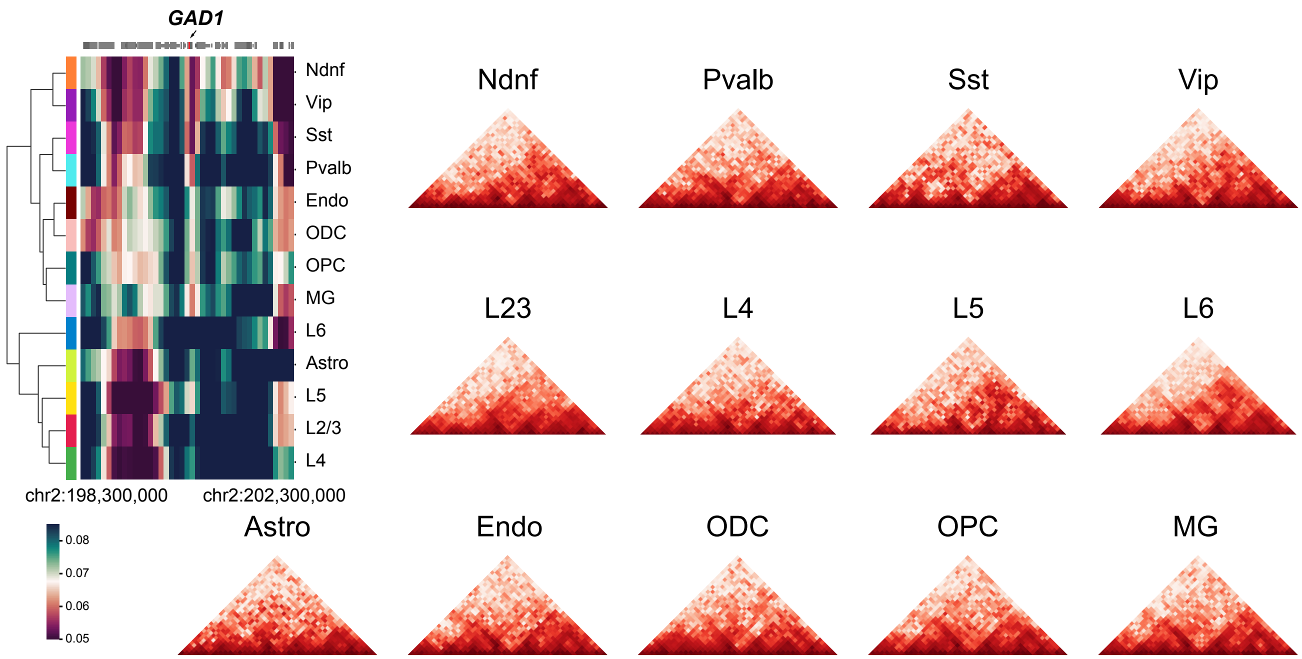

Figure S23: Hierarchical clustering based on the average single-cell insulation scores of the flanking regions (+/- 2Mbp) of the marker gene GAD1 for inhibitory neuron subtypes Sst, Pvalb, Ndnf, and Vip. Note that the single-cell insulation scores are calculated based on the raw scHi-C contact maps. The pooled raw scHi-C contact maps of the same region are also visualized for comparison in each cell subtype. Related to Fig. 4c,d.

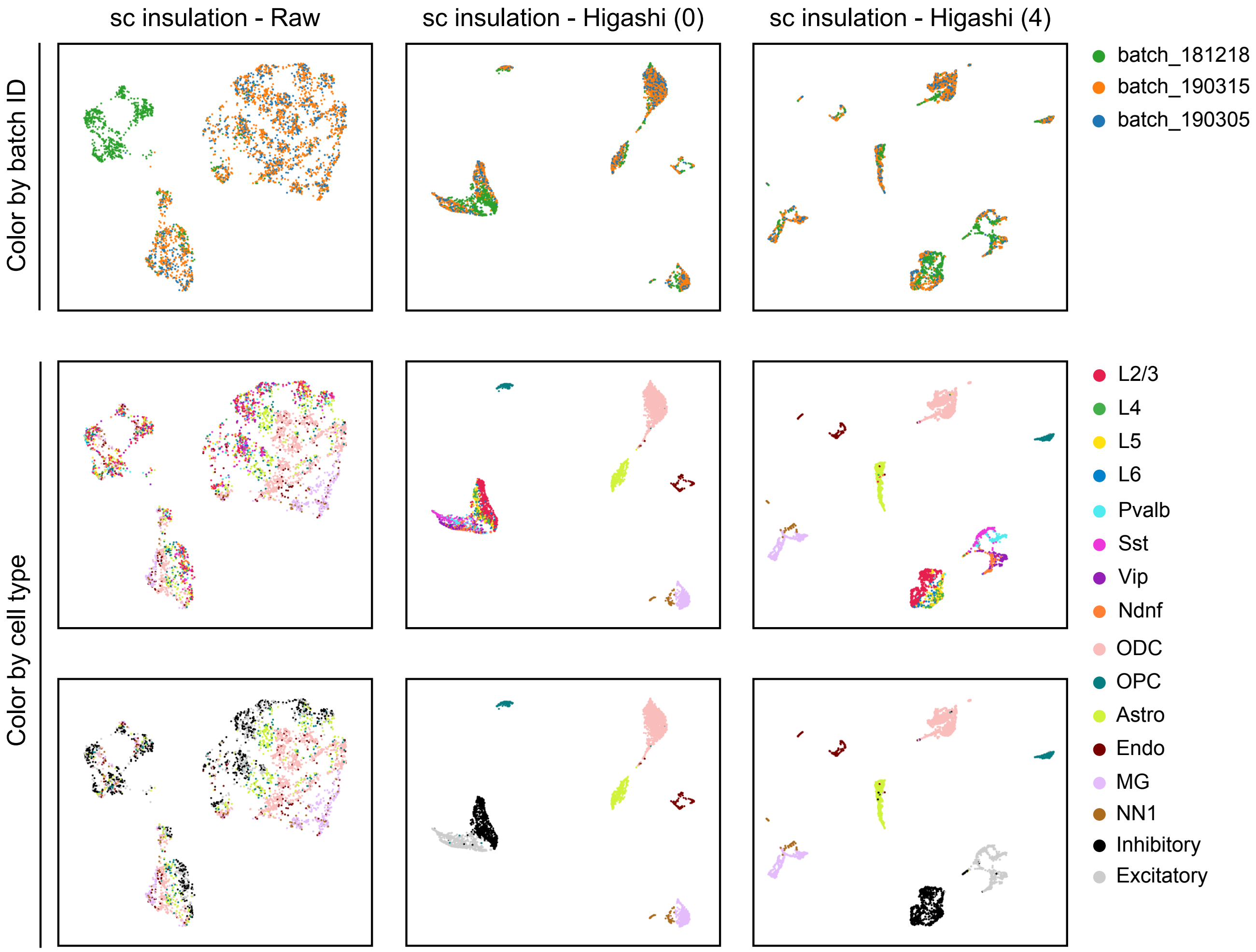

Figure S24: UMAP visualization of single-cell insulation scores based on the sn-m3c-seq dataset from human PFC. All single-cell insulation scores are reduced to 32-dimensional vectors before passing to UMAP. The rows are organized by different color schemes (i.e., batch ID or cell type). The columns correspond to the single cell insulation scores calculated from the raw contact maps or the Higashi imputed contact maps.

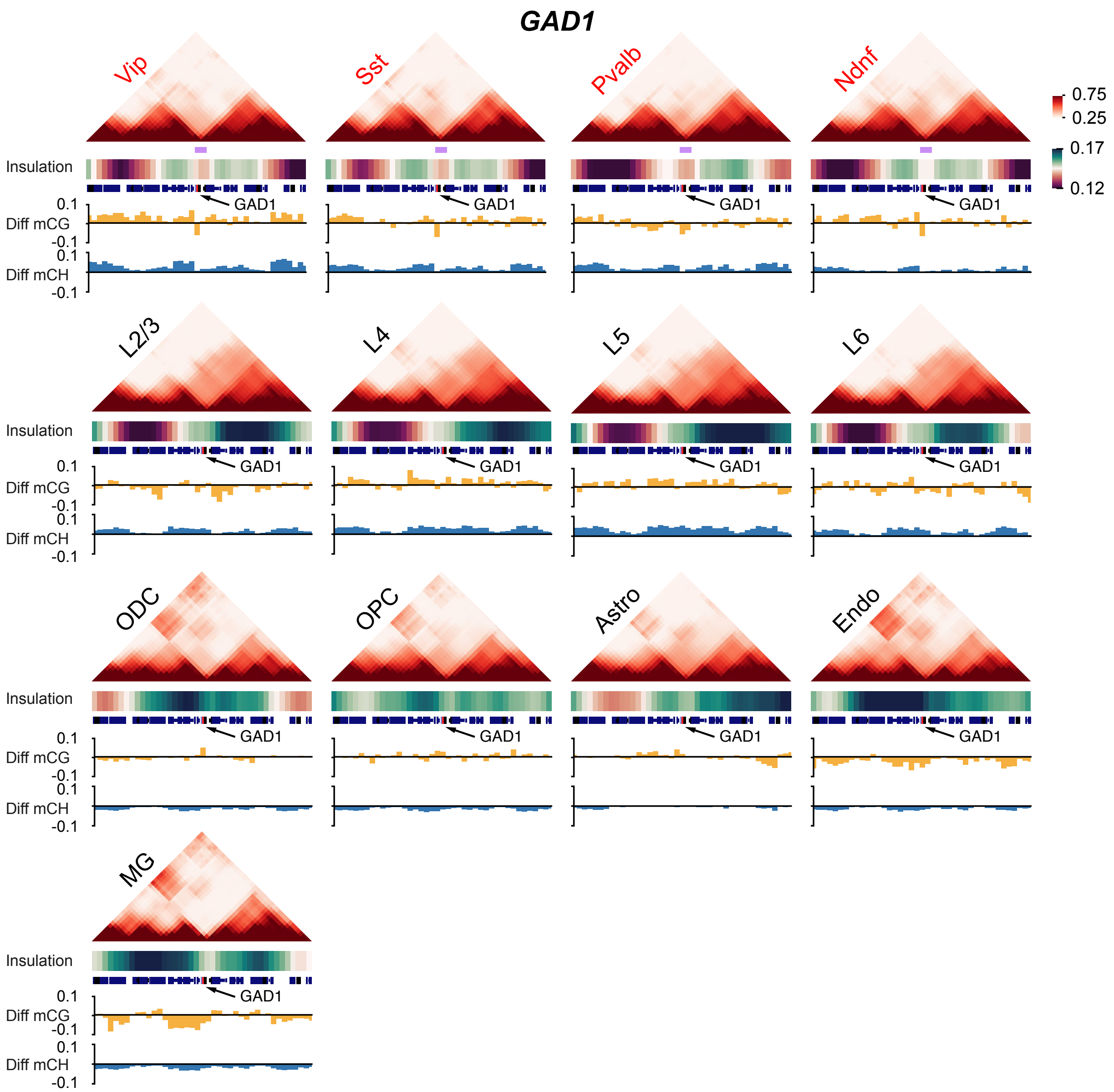

Figure S25: Pooled imputed contact maps, insulation scores, and methylation profiles of the flanking regions (+/- 2Mbp) of the marker gene GAD1 for inhibitory neuron subtypes Sst, Pvalb, Ndnf, and Vip. Light purple bar shows a TAD-like domain boundary specific to inhibitory neuron subtypes. Related to Fig. 4d.

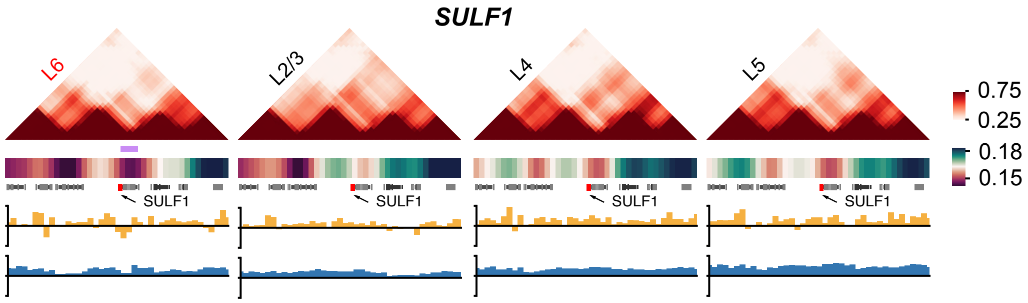

Figure S26: Pooled imputed contact maps, insulation scores, and methylation profiles of the flanking regions (+/- 2Mbp) of the marker gene SULF1 to distinguish L6 subtypes from the rest excitatory neuron subtypes (L2/3, L4, L5). Light purple bar shows a stronger TAD-like domain boundary specific to L6.

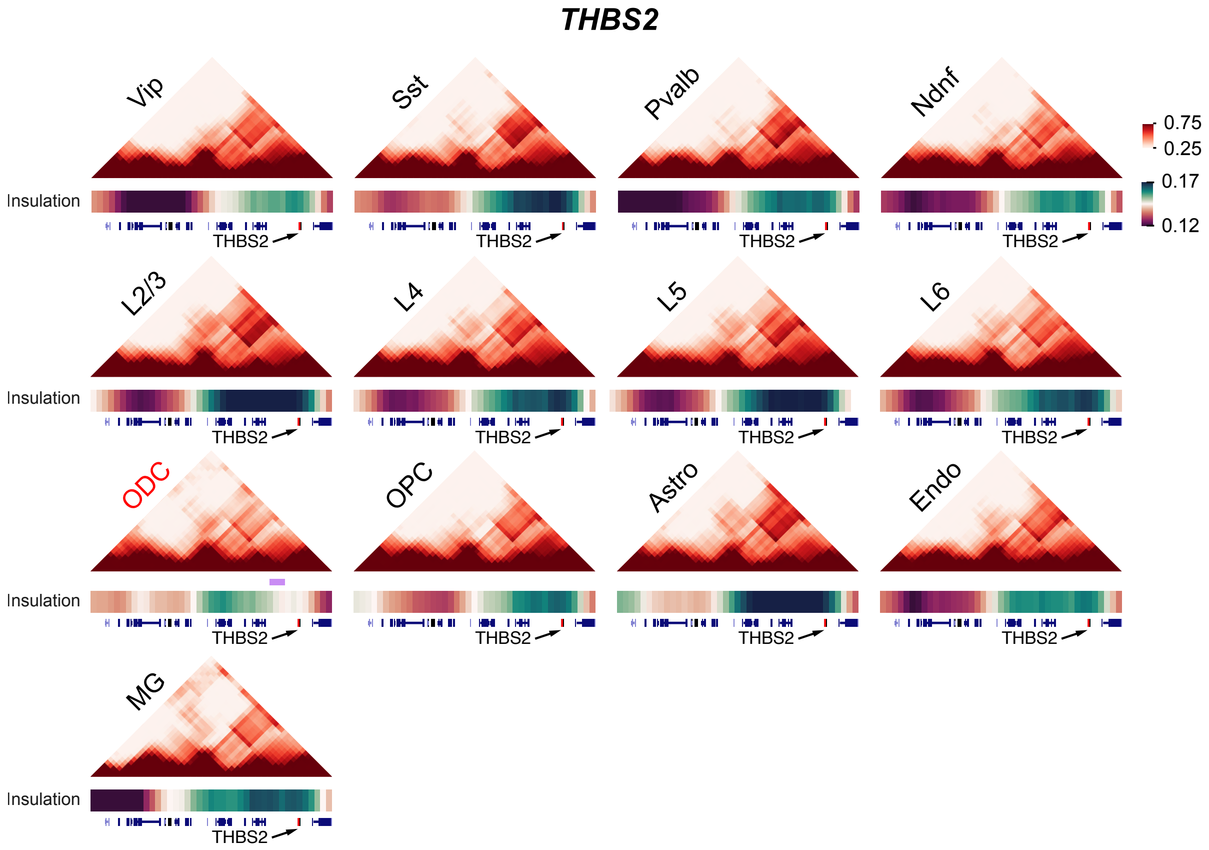

Figure S27: Pooled imputed contact maps, insulation scores, and methylation profiles of the surrounding regions (+0.5Mbp/-3.5Mbp) of the gene THBS2 that is near a cell type-specific TAD-like boundary in ODC and has cell type-specific high expression in ODC. Light purple bar shows an ODC-specific TAD-like domain boundary. Related to Fig. 4f.

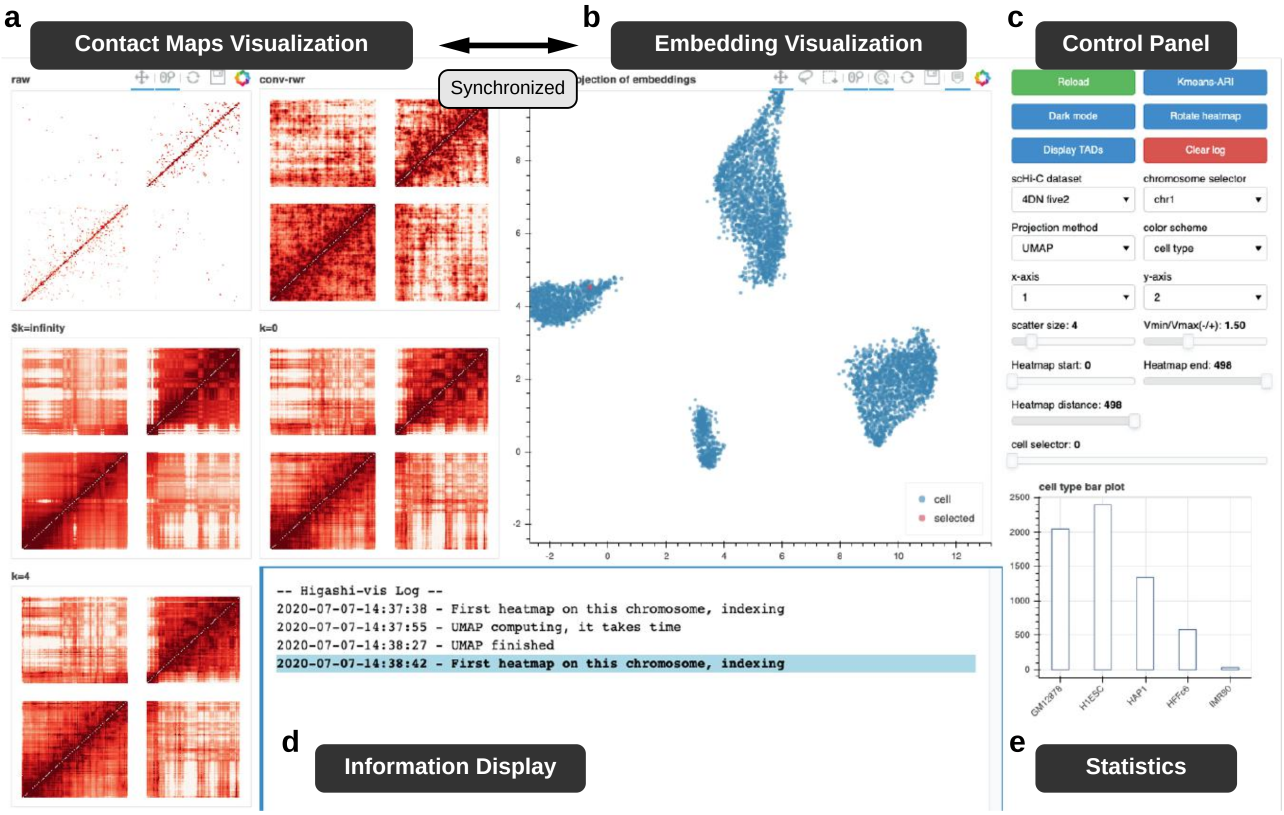

Figure S28: Screenshot of the visualization tool in Higashi. **a.** Visualization of the single cell (pooled) contact maps before and after imputation with different methods. This panel is synchronized for visualization with panel **b**. **b.** Visualization of the scHi-C embeddings where each dot corresponds to one cell. Clicking or selecting cell(s) of interest will update the panel **a** interactively. **c.** Control panel for the visualization such as the color scheme of the embedding scatter plot in panel **a,b**. **d.** Display messages from the server. **e.** Visualization of statistics of the cell population as well as the selected cells in panel **b**. Note that this panel will also update interactively.
