# Supplementary material for: Multiscale and integrative single-cell Hi-C analysis with Higashi
Source: Nat Biotechnol. 2021 Oct 11;40(2):254–61. doi: 10.1038/s41587-021-01034-y (PMC8843812; doi:10.1038/s41587-021-01034-y)
Supplement: Supplementary file 2 — Reporting Summary [file 41587_2021_1034_MOESM2_ESM.pdf]

nature research

Corresponding author(s): Jian Ma

Last updated by author(s): Jul 20, 2021

Reporting Summary

Nature Research wishes to improve the reproducibility of the work that we publish. This form provides structure for consistency and transparency in reporting. For further information on Nature Research policies, see our Editorial Policies and the Editorial Policy Checklist.

Statistics

For all statistical analyses, confirm that the following items are present in the figure legend, table legend, main text, or Methods section.

n/a	Confirmed	
☐	☒	The exact sample size (n) for each experimental group/condition, given as a discrete number and unit of measurement
☐	☒	A statement on whether measurements were taken from distinct samples or whether the same sample was measured repeatedly
☐	☒	The statistical test(s) used AND whether they are one- or two-sided<br>*Only common tests should be described solely by name; describe more complex techniques in the Methods section.*
☐	☒	A description of all covariates tested
☐	☒	A description of any assumptions or corrections, such as tests of normality and adjustment for multiple comparisons
☐	☒	A full description of the statistical parameters including central tendency (e.g. means) or other basic estimates (e.g. regression coefficient) AND variation (e.g. standard deviation) or associated estimates of uncertainty (e.g. confidence intervals)
☐	☒	For null hypothesis testing, the test statistic (e.g. F, t, r) with confidence intervals, effect sizes, degrees of freedom and P value noted<br>*Give P values as exact values whenever suitable.*
☒	☐	For Bayesian analysis, information on the choice of priors and Markov chain Monte Carlo settings
☒	☐	For hierarchical and complex designs, identification of the appropriate level for tests and full reporting of outcomes
☐	☒	Estimates of effect sizes (e.g. Cohen's d, Pearson's r), indicating how they were calculated

Our web collection on statistics for biologists contains articles on many of the points above.

Software and code

Policy information about availability of computer code

Data collection	No software was used for data collection.
Data analysis	All analysis was done on CentOS 7. The source code of Higashi (software reported in this manuscript) is publicly available at https://github.com/ma-compbio/Higashi. We used the following open source Python 3.7.9 packages: h5py (2.10.0), numpy (1.19.2), pandas (1.1.3), pytorch (1.4.0), fbpca (1.0.0), scikit-learn (0.23.2), tqdm (4.50.2), seaborn (0.11.0), matplotlib(3.3.2), umap-learn(0.4.6), bokeh (2.1.1), PIL (7.2.0), cachetools (4.1.1). The adam algorithm we used for optimizing the neural network is implemented in pytorch (1.4.0). We benchmarked against scHiCluster (commit version c9c1fd0), HiCRep/MDS (hicreppy 0.0.6, scikit-learn 0.23.2), and LDA (https://github.com/khj3017/schic-topic-model commit version 7c1ea3e) with citations in the manuscript. We used Seurat v3.0.0 for scRNA-seq analysis. We used Scanorama v1.6 for generating embeddings of single-cell CG methylation profiles. All source code was obtained from the latest GitHub repositories of these methods.

For manuscripts utilizing custom algorithms or software that are central to the research but not yet described in published literature, software must be made available to editors and reviewers. We strongly encourage code deposition in a community repository (e.g. GitHub). See the Nature Research guidelines for submitting code & software for further information.

Data

Policy information about availability of data

All manuscripts must include a data availability statement. This statement should provide the following information, where applicable:

- Accession codes, unique identifiers, or web links for publicly available datasets
- A list of figures that have associated raw data
- A description of any restrictions on data availability

The scHi-C datasets that support the findings of this study were obtained through publicly available repositories (citations in the manuscript).

• The Ramani et al. dataset from the NCBI Gene Expression Omnibus (GEO) (http://www.ncbi.nlm.nih.gov/geo/) under accession number GSE84920.
• The Nagano et al. dataset from https://bitbucket.org/tanaylab/schic2/src/default/.
• The 4DN sci-Hi-C dataset from the 4DN Data Portal (https://data.4dnucleome.org/) under accession number 4DNES4D5MWEZ, 4DNESUE2NSGS, 4DNESIKGI39T, 4DNES1BK1RMQ, and 4DNESTVIP977.
• The WTC-11 scHi-C dataset from the 4DN Data Portal (https://data.4dnucleome.org/) under accession number 4DNESF829JOW, 4DNESJQ4RXY5.
• The sn-m3c-seq dataset from GEO accession GSE130711.

Other epigenomic datasets that support the findings of this study were obtained through publicly available repositories as well (citations in the manuscript).
• The scRNA-seq of WTC-11 from the European Bioinformatics Institute (EMBL-EBI) (https://www.ebi.ac.uk/) under accession number E-MTAB-6268 and E-MTAB-6687.
• The CTCF ChIA-PET data of WTC-11 from the 4DN Data Portal (https://data.4dnucleome.org/) under accession number 4DNES8MZ76GP.
• The scRNA-seq of human prefrontal cortex from Allen Brain Map (https://portal.brain-map.org/).

Field-specific reporting

Please select the one below that is the best fit for your research. If you are not sure, read the appropriate sections before making your selection.

☒ Life sciences ☐ Behavioural & social sciences ☐ Ecological, evolutionary & environmental sciences

For a reference copy of the document with all sections, see nature.com/documents/nr-reporting-summary-flat.pdf

Life sciences study design

All studies must disclose on these points even when the disclosure is negative.

Sample size	We evaluated our method by using five available scHi-C datasets (Supplementary Table S1) and an additionally Dip-C dataset. These datasets represent scHi-C datasets with different number of cells, different technologies, and different sequencing depths. Therefore, the datasets are sufficient to demonstrate the effectiveness and robustness of our method.
Data exclusions	Cells of each dataset were filtered following a procedure described in Methods. For all scHi-C datasets, we only kept the cells with more than 2,000 read pairs with genomic span greater than 500Kb.
Replication	Replication of results was performed when possible. For example, in the analysis on the CTCF binding near the single cell TAD-like domain boundaries are replicated on individual CTCF ChIA-PET sequencing library as well as merged library.
Randomization	The training/validation set was randomly partitioned during the training of our method and during the evaluation of the cell embeddings on supervised cell type classification tasks.
Blinding	In our evaluations and analysis, the cell type label was blinded from the model when learning embeddings and during imputation.

Reporting for specific materials, systems and methods

We require information from authors about some types of materials, experimental systems and methods used in many studies. Here, indicate whether each material, system or method listed is relevant to your study. If you are not sure if a list item applies to your research, read the appropriate section before selecting a response.

Materials & experimental systems

n/a	Involved in the study	
☒	☐	Antibodies
☒	☐	Eukaryotic cell lines
☒	☐	Palaeontology and archaeology
☒	☐	Animals and other organisms
☒	☐	Human research participants
☒	☐	Clinical data
☒	☐	Dual use research of concern

Methods

n/a	Involved in the study	
☒	☐	ChIP-seq
☒	☐	Flow cytometry
☒	☐	MRI-based neuroimaging
